# Design, Synthesis and Antifungal Activities of Novel Pyrazole Analogues Containing the Aryl Trifluoromethoxy Group

**DOI:** 10.3390/molecules28176279

**Published:** 2023-08-28

**Authors:** Tongchao Zhao, Yuyao Sun, Yufei Meng, Lifang Liu, Jingwen Dai, Guoan Yan, Xiaohong Pan, Xiong Guan, Liyan Song, Ran Lin

**Affiliations:** Key Laboratory of Biopesticide and Chemical Biology, Ministry of Education, College of Plant Protection, Fujian Agriculture and Forestry University, Fuzhou 350002, Chinayanguoan01@163.com (G.Y.); panxiaohong@163.com (X.P.); guanxfafu@126.com (X.G.)

**Keywords:** pyrazole analogues, pathogenic fungi, antifungal activity, structure–activity relationship

## Abstract

On the basis of the three-component synthetic methodology developed by us, a total of twenty-six pyrazole compounds bearing aryl OCF_3_ were designed and synthesized. Their chemical structures were characterized by ^1^H and ^13^C nuclear magnetic resonance and high-resolution mass spectrometry. These compounds were evaluated systematically for antifungal activities in vitro against six plant pathogenic fungi by the mycelium growth rate method. Most of the compounds showed some activity against each of the fungi at 100 μg/mL. Compounds **1t** and **1v** exhibited higher activity against all the tested fungi, and **1v** displayed the highest activity against *F. graminearum* with an EC_50_ value of 0.0530 μM, which was comparable with commercial pyraclostrobin. Structure–activity relationship analysis showed that, with respect to the R_1_ substituent, the straight chain or cycloalkyl ring moiety was a key structural moiety for the activity, and the R_2_ substituent on the pyrazole ring could have significant effects on the activity. Simple and readily available pyrazoles with potent antifungal activity were obtained, which are ready for further elaboration to serve as a pharmacophore in new potential antifungal agents.

## 1. Introduction

Phytopathogenic fungus is a serious problem worldwide and is highly detrimental to agricultural production, causing severe yield losses and quality decreases [1,2]. The fungal infections even produce mycotoxins that might result in serious damage to human health [3]. Currently, chemical control is still the most effective measure to prevent these harmful diseases, and several classes of effective agrochemicals have been produced to arrest the damaging activities of phytopathogenic fungi against food crops [4,5,6]. However, the long-term and large-scale application of antifungal agents with single target sites has led to the growing resistance of plant pathogens to fungicides [7,8]. Therefore, there is an urgent need to develop new fungicides with novel chemical structures and action mechanisms that are efficient, low-toxicity, and environmentally benign.

**Figure 1:**
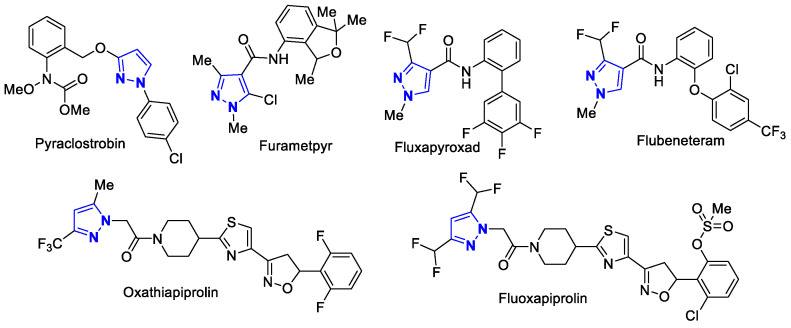
Selected commercialized fungicides with a pyrazole moiety.

The pyrazole derivatives are an important class of nitrogen-containing five-membered heterocyclic compounds that have attracted much attention from the synthetic community recently [9,10] due to their unique performance in the field of drug and agrochemical research. They exhibit a wide variety of biological activities, including antibacterial, antifungal [11,12,13], anti-inflammatory, anticancer, anti-tubercular, antiviral, and anti-leishmanial activities [14,15,16,17]. To date, many commercial fungicides with a pyrazole moiety have been approved for plant protection (Figure 1). For example, the classical fungicide pyraclostrobin could protect various crops from a wide spectrum of phytopathogenic fungi by disrupting the energy cycle within the fungus [18]. Furametpyr, Fluxapyroxad, and Flubeneteram, as commercial fungicides, are respiration inhibitors, blocking mitochondrial complex II [19]. Oxathiapiprolin and fluoxapiprolin exhibited great control efficacies against pathogenic oomycetes and acted at the oxysterol binding protein, which is a novel binding site [20]. The fungicides with a pyrazole moiety have been applied to manage a variety of diseases in crops, fruits, and vegetables.

**Figure 2:**
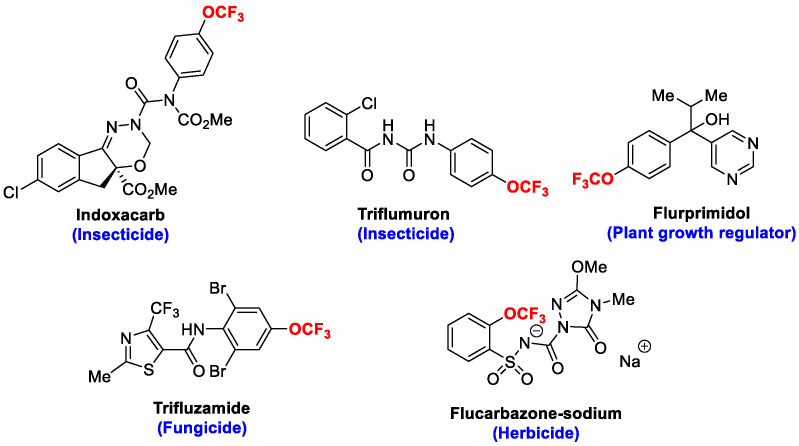
Representative OCF_3_-bearing compounds in agrochemicals.

The installation of OCF_3_ into the proper positions of agrochemicals would lead to efficiently enhanced chemical and metabolic stability as well as improved lipophilicity and bioavailability [21]. Moreover, as the OCF_3_ group is attached to an aromatic ring, a unique and stable conformation where the dihedral angle of the C=C-O-CF_3_ bond is close to 90° would be set [22]. As a result, organic compounds containing aryl trifluoromethyl ether may possess additional binding affinity with the active sites of the target [23], which was applied in the discovery of agricultural chemicals to furnish commercially available OCF_3_-containing agrochemicals (Figure 2) [24,25,26].

**Figure 3:**
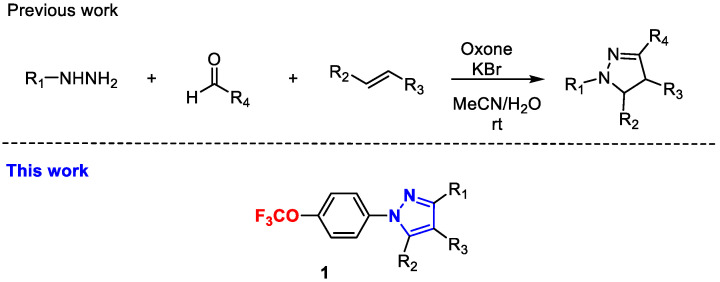
Design of the chemical structure containing pyrazole and aryl OCF_3_.

In 2022, we reported the first green three-component oxidative cyclization for a one-pot synthesis of 2-pyrazolines in good-to-high yield and excellent regioselectivity from commercially available arylhydrazines (ArNHNH_2_), aldehydes, and alkenes (Figure 3) [27]. Prompted by the advantages of pyrazole and aryl trifluoromethyl ether derivatives in combination with our powerful synthetic methodology, we aim to design a novel chemical structure containing pyrazole and aryl OCF_3_ moieties, synthesize a series of pyrazole analogues (**1**, Figure 3) by introducing the active moiety of aryl trifluoromethyl ether, and further investigate the antifungal activities to seek more potent lead compounds for promoting the development of pyrazole agrochemicals. Herein, we report the synthesis of a new series of twenty-six pyrazole compounds for screening in vitro antifungal activities against six strains of phytopathogenic fungi (*Botrytis cinerea*, *Magnaporthe oryzae*, *Pythium aphanidermatum*, *Fusarium graminearum*, *Colletotrichum micotianae*, and *Valsa mali*). The structure–activity relationship was deduced based on the experimental results of antifungal activities.

## 2. Results

### 2.1. Chemistry

**Scheme 1:**
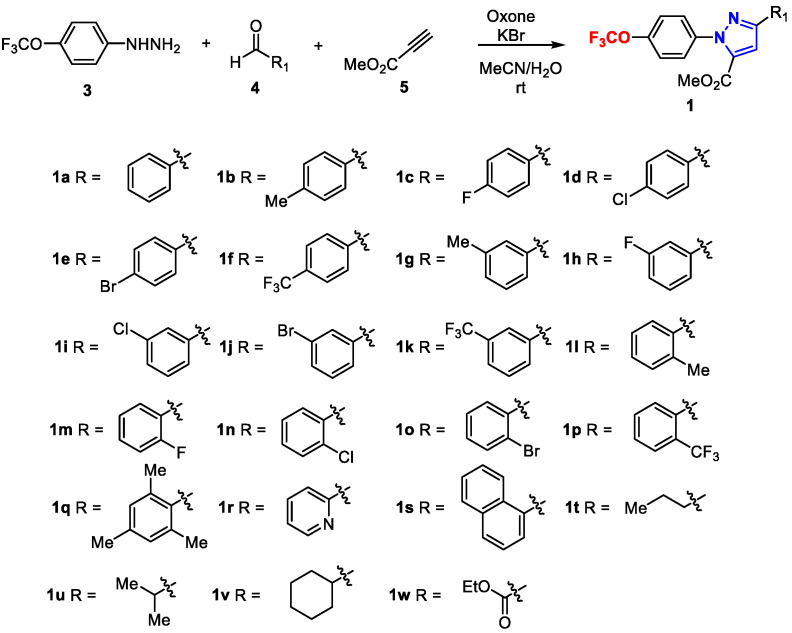
Synthesis of pyrazole analogues where R_3_ = H (**1a**–**1w**).

**Scheme 2:**
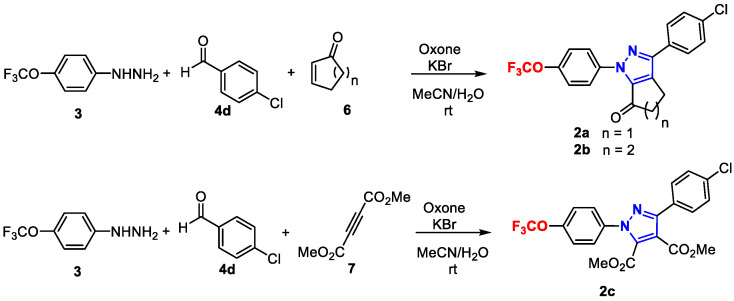
Synthesis of pyrazole analogues where R_3_ ≠ H (**2a**–**2c**).

The general synthetic routes of target compounds were depicted in Scheme 1 and Scheme 2. Similar green three-component oxidative cyclization was applied to afford 2-pyrazoles (**1**) in good-to-high yield from commercially available arylhydrazine (**3**. ArNHNH_2_), aldehydes (**4**), and methyl propiolate (**5**) (Scheme 1). Gratifyingly, the methyl propiolate was quite competent in the reaction system, and the desired products were secured in a one-pot manner. Noteworthy is that excellent regioselectivity was observed and little regioisomer was detected. The structures of the synthesized compounds were confirmed by spectroscopic analysis, including ^1^H NMR, ^13^C NMR, and HRMS. **1d** was a known compound, the spectra of which were identical to our previous report [27]. The substitution pattern of the pyrazole ring (**1a**–**1c**, **1e**–**1s**) was set according to **1d** with similar aryl substitution and reaction conditions. In addition, the NMR spectra could also provide a lot of information. **1u** was selected to interpret the NMR spectra. We could obviously see the *para*-substituted phenyl ring from the two doublets at 7.47 and 7.29 ppm. The singlet at 6.87 ppm represents the sole hydrogen in the pyrazole ring. The OMe group falls in the singlet at 3.80 ppm, while the isopropyl group was observed in the multiplet at 3.06 ppm and the doublet at 1.31 ppm. In addition, the regio-configuration of **1u** was established by 1D nOe experiments, for we could observe the nOe between the pyrazolic hydrogen and isopropyl group (see Supporting Information). The ^13^C NMR spectra closely paralleled the structure deduced above. Furthermore, the OCF_3_ was observed as a quartet with a large coupling constant (C–F, 256 Hz).

Then we turn to pyrazoles bearing R_3_ substitutions. Bicyclic compounds (**2a**–**2b**) and diester (**2c**) are known compounds and were attained according to our previous work [27].

### 2.2. In Vitro Antifungal Activity

Using the method of mycelium linear growth rate reported previously by us [28,29], the in vitro fungicidal activities of all candidates (**1a**–**1w**, **2a**–**2c**) were screened at a concentration of 100 μg/mL against six strains of phytopathogenic fungi (*Botrytis cinerea*, *Magnaporthe oryzae*, *Pythium aphanidermatum*, *Fusarium graminearum*, *Colletotrichum micotianae*, and *Valsa mali*). The commercial fungicide pyraclostrobin was selected as a positive control. The results of the preliminary antifungal activities of compounds are listed in Table 1 and show that most of the compounds exhibited some antifungal activity in different degrees against each of the test fungi. In general, compounds **1t**, **1v**, and **2a**–**2c** exhibited higher activity than other tested compounds. To our surprise, compounds **1a**–**1s** possessing the conjugated aryl structure failed to exhibit potent antifungal activity. Notably, for the fungus *Fusarium graminearum*, compound **1v** showed good activity with an inhibition rate of 81.22%, which was slightly higher than that of pyraclostrobin, a commercial fungicide. Furthermore, the compound **1v** exhibited good activity against *Colletotrichum micotianae* with an inhibition rate of 56.03%, which was not far from that of the positive control pyraclostrobin. Moreover, compound **1v** was active against all tested plant pathogenic fungi, exhibiting a broader antifungal spectrum. Two strains of fungi, including *F. graminearum* and *C. micotianae,* were relatively sensitive to **1p**, **1t**, and **2a**–**2c**, among which **1t** performed best over others.

In order to further explore the antifungal potential, the compounds with inhibition rates higher or close to positive control (in red) were further examined to determine their median effective concentrations (EC_50_) against *F. graminearum* and/or *C. micotianae*. The calculated results are shown in Table 2.

From Table 2, it was clearly seen that **1v** and **1t** showed medium-to-good activity, with EC_50_ values of 0.0530–0.1430 μM. For *F. graminearum*, target compounds **1v** and **1t** showed good activity, with an EC_50_ value of 0.0530–0.0735 μM, which was comparable with commercial pyraclostrobin. As for *C. micotianae*, only compound **1v** showed medium activity with an EC_50_ value of 0.1430 μM, which is not far from that of pyraclostrobin.

## 3. Discussion

From the comparisons of the inhibition rates at 100 μg/mL in Table 1, in combination with the EC_50_ values in Table 2, it could be concluded that, with regard to the R_1_ substituent, the presence of a phenyl group would lead to a significant decrease in the activity against all test fungi in most cases, regardless of electronic property and steric hindrance in a phenyl ring. However, the presence of the *ortho* or *meta* trifluoromethyl (CF_3_) group would slightly increase activity (**1k**, **1p**). The introduction of pyridine (**1r**), naphthalene (**1s**), or ester (**1w**) groups to the R_1_ position led to a decrease in the antifungal activity. It should be noted that the introduction of a straight-chain substituent (**1t**) or cycloalkyl group (**1v**) would significantly increase activity against almost all tested fungi. Most noteworthy was that the antifungal activity of compound **1v** bearing a cycloalkyl substituent against *F. graminearum* prevailed over that of commercial pyraclostrobin at 100 μg/mL. Moreover, we observed that the secondary acyclic alkyl substituent (**1u**) failed to exhibit potent activity in sharp contrast to the secondary cyclic alkyl substituent (**1v**), which should be considered in our further design and elaboration of the antifungal pyrazole structure. Then we turned to R_2_ and R_3_ substituents and found that substituted R_3_ would significantly improve the antifungal activity against all tested fungi by comparing **1d** with **2a**–**2c**.

## 4. Materials and Methods

### 4.1. General Information

All reagents and chemicals were purchased from Energy Chemical Co. Ltd. (Shanghai, China) and used without further purification. All solvents were dried and redistilled prior to use. The melting points were measured on a digital apparatus (M560 Buchi) and uncorrected. Nuclear magnetic resonance (NMR) spectra were performed on a Bruker AVIII 400 MHz instrument. Chemical shifts were measured relative to residual solvent peaks of chloroform-d as internal standards (7.26 ppm for ^1^H and 77.16 ppm for ^13^C). The following abbreviations were used to designate chemical shift multiplicities: s, singlet; d, doublet; t, triplet; m, multiplet. High-resolution mass spectra (HRMS) were recorded with a Thermo Scientific LTQ Orbitrap XL system or a Bruker solariX System.

### 4.2. Synthetic Procedures

The synthetic route of desired compounds **1a**–**1w** and **2a**–**2c** was outlined in Scheme 1 and Scheme 2. Additionally, the title compounds were synthesized according to our previously reported procedure [27]. To a stirred solution of hydrazine **3** (0.3 mmol), aldehyde **4** (0.33 mol), and corresponding alkyne or alkene **5**, **6**, or **7** (0.6 mmol) in MeCN/H_2_O (10/1, 1.1 mL) at 0 °C, KBr (43 mg, 1.2 equiv.), K_2_CO_3_ (103 mg, 2.5 equiv.), and Oxone (276 mg, 3 equiv.) were added. After completion of the addition, the resulting mixture was stirred for 10 min before being warmed to room temperature and stirred for an additional 9 h. The reaction’s progress was monitored by TLC. The reaction was quenched by the addition of saturated aqueous Na_2_SO_3_ (5 mL). The organic layer was collected, and the aqueous layer was extracted with EA (3 × 10 mL). The combined organic fractions were washed with brine, dried over anhydrous Na_2_SO_4_, and concentrated under reduced pressure. The residue was purified by flash column chromatography on silica gel using eluents (petroleum ether/ethyl acetate = 10/1) to furnish the desired compound.

**1a**. 84 mg, 77% yield; White solid. m.p. = 134–136 °C. **^1^H NMR** (400 MHz, CDCl_3_) δ: 7.88–7.85 (m, 2H), 7.56 (d, *J* = 8.8 Hz, 2H), 7.46–7.42 (m, 2H), 7.39–7.32 (m, 4H), 3.85 (s, 3H). **^13^C NMR** (100 MHz, CDCl_3_) δ: 159.6, 152.0, 149.2 (q, *J* = 1.9 Hz), 138.7, 134.3, 131.9, 129.0 (2×C), 128.8, 127.7 (2×C), 125.9 (2×C), 121.2 (2×C), 120.5 (q, *J* = 256 Hz), 110.0, 52.4. **^19^F NMR** (376 MHz, CDCl_3_) δ: −57.8 (3×F). **HRMS** (ESI) *m*/*z* calculated for C_18_H_14_O_3_N_2_F_3_^+^ [M+H]^+^ 363.0951, found 363.0956.

**1b**. 89 mg, 79% yield; White solid. m.p. = 124–126 °C. **^1^H NMR** (400 MHz, CDCl_3_) δ: 7.75 (d, *J* = 8.0 Hz, 2H), 7.55 (d, *J* = 8.8 Hz, 2H), 7.32 (d, *J* = 8.8 Hz, 2H), 7.31 (s, 1H) 7.24 (d, *J* = 8.0 Hz, 2H), 3.84 (s, 3H), 2.39 (s, 3H). **^13^C NMR** (100 MHz, CDCl_3_) δ: 159.6, 152.1, 149.1, 138.7, 138.6, 134.2, 129.6 (2×C), 129.1, 127.7 (2×C), 125.8 (2×C), 121.2 (2×C), 120.5 (q, *J* = 256 Hz), 109.9, 52.3, 21.5. **^19^F NMR** (376 MHz, CDCl_3_) δ: −57.8 (3×F). **HRMS** (ESI) *m*/*z* calculated for C_19_H_16_O_3_N_2_F_3_^+^ [M+H]^+^ 377.1108, found 377.1114.

**1c**. 90 mg, 79% yield; White solid. m.p. = 109–111 °C. **^1^H NMR** (400 MHz, CDCl_3_) δ: 7.83 (dd, *J* = 8.8, 5.2 Hz, 2H), 7.54 (d, *J* = 8.8 Hz, 2H), 7.33 (d, *J* = 8.8 Hz, 2H), 7.29 (s, 1H), 7.12 (t, *J* = 8.8 Hz, 2H), 3.84 (s, 3H). **^13^C NMR** (100 MHz, CDCl_3_) δ: 163.2 (d, *J* = 246 Hz), 159.5, 151.1, 149.2 (q, *J* = 1.9 Hz), 138.6, 134.5, 128.2 (d, *J* = 3.2 Hz), 127.7 (2×C), 127.6 (d, *J* = 7.4 Hz, 2×C), 121.2 (2×C), 120.5 (q, *J* = 256 Hz), 115.9 (d, *J* = 21.7 Hz, 2×C), 109.8, 52.4. **^19^F NMR** (376 MHz, CDCl_3_) δ: −113.0, −57.8 (3×F). **HRMS** (ESI) *m*/*z* calculated for C_18_H_13_O_3_N_2_F_4_^+^ [M+H]^+^ 381.0857, found 381.0860.

**1d [27]**. 96 mg, 81% yield; White solid. **^1^H NMR** (400 MHz, CDCl_3_) δ: 7.79 (d, *J* = 8.8 Hz, 2H), 7.54 (d, *J* = 9.2 Hz, 2H), 7.40 (d, *J* = 8.8 Hz, 2H), 7.33 (d, *J* = 8.8 Hz, 2H), 7.31 (s, 1H), 3.84 (s, 3H). **^13^C NMR** (100 MHz, CDCl_3_) δ: 159.5, 150.9, 149.2, 138.5, 134.6, 134.5, 130.5, 129.2 (2×C), 127.7 (2×C), 127.2 (2×C), 121.2 (2×C), 120.5 (q, *J* = 256 Hz), 109.9, 52.4.

**1e**. 110 mg, 83% yield; White solid. m.p. = 149–151 °C. **^1^H NMR** (400 MHz, CDCl_3_) δ: 7.73 (d, *J* = 8.8 Hz, 2H), 7.56 (d, *J* = 8.8 Hz, 2H), 7.54 (d, *J* = 8.8 Hz, 2H), 7.33 (d, *J* = 8.8 Hz, 2H), 7.32 (s, 1H), 3.84 (s, 3H). **^13^C NMR** (100 MHz, CDCl_3_) δ: 159.4, 150.9, 149.2 (q, *J* = 1.9 Hz), 138.5, 134.5, 132.1 (2×C), 130.9, 127.7 (2×C), 127.4 (2×C), 122.8, 121.2 (2×C), 120.5 (q, *J* = 256 Hz), 109.9, 52.4. **^19^F NMR** (376 MHz, CDCl_3_) δ: −57.8 (3×F). **HRMS** (ESI) *m*/*z* calculated for C_18_H_13_O_3_N_2_F_3_Br^+^ [M+H]^+^ 441.0056, found 441.0057.

**1f**. 90 mg, 70% yield; White solid. m.p. = 107–109 °C. **^1^H NMR** (400 MHz, CDCl_3_) δ: 7.98 (d, *J* = 8.0 Hz, 2H), 7.69 (d, *J* = 8.0 Hz, 2H), 7.56 (d, *J* = 8.8 Hz, 2H), 7.39 (s, 1H), 7.35 (d, *J* = 8.8 Hz, 2H), 3.86 (s, 3H). **^13^C NMR** (100 MHz, CDCl_3_) δ: 159.4, 150.6, 149.3, 138.5, 135.4, 134.7, 130.6 (q, *J* = 32.3 Hz), 127.7 (2×C), 126.1 (2×C), 125.9 (q, *J* = 3.8 Hz, 2×C), 124.2 (q, *J* = 270 Hz), 121.2 (2×C), 120.5 (q, *J* = 256 Hz), 110.3, 52.5. **^19^F NMR** (376 MHz, CDCl_3_) δ: −62.6 (3×F), −57.8 (3×F). **HRMS** (ESI) *m*/*z* calculated for C_19_H_13_O_3_N_2_F_6_^+^ [M+H]^+^ 431.0825, found 431.0834.

**1g**. 79 mg, 70% yield; White solid. m.p. = 123–125 °C. **^1^H NMR** (400 MHz, CDCl_3_) δ: 7.71 (s, 1H), 7.64 (d, *J* = 7.6 Hz, 1H), 7.56 (d, *J* = 8.8 Hz, 2H), 7.34–7.30 (m, 4H), 7.19 (d, *J* = 7.6 Hz, 1H), 3.84 (s, 3H), 2.41 (s, 3H). **^13^C NMR** (100 MHz, CDCl_3_) δ: 159.6, 152.2, 149.2, 138.7, 138.6, 134.3, 131.8, 129.6, 128.8, 127.8 (2×C), 126.5, 123.1, 121.2 (2×C), 120.5 (q, *J* = 256 Hz), 110.1, 52.4, 21.6. **^19^F NMR** (376 MHz, CDCl_3_) δ: −57.8 (3×F). **HRMS** (ESI) *m*/*z* calculated for C_19_H_16_O_3_N_2_F_3_^+^ [M+H]^+^ 377.1108, found 377.1101.

**1h**. 82 mg, 72% yield; White solid. m.p. = 118–120 °C. **^1^H NMR** (400 MHz, CDCl_3_) δ: 7.63 (dt, *J* = 7.6, 1.2 Hz, 1H), 7.59–7.53 (m, 1H), 7.55 (d, *J* = 8.8 Hz, 2H), 7.40 (dt, *J* = 6.0, 8.0 Hz, 1H), 7.34 (d, *J* = 8.8 Hz, 2H), 7.33 (s, 1H), 7.06 (tdd, *J* = 8.8, 2.4, 0.8 Hz, 1H), 3.85 (s, 3H). **^13^C NMR** (100 MHz, CDCl_3_) δ: 163.3 (d, *J* = 244 Hz), 159.4, 150.9 (d, *J* = 2.7 Hz), 149.2 (q, *J* = 1.9 Hz), 138.5, 134.5, 134.1 (d, *J* = 8.4 Hz), 130.5 (d, *J* = 8.2 Hz), 127.7 (2×C), 121.5 (d, *J* = 2.8 Hz), 121.2 (2×C), 120.5 (q, *J* = 256 Hz), 115.6 (d, *J* = 21 Hz), 112.8 (d, *J* = 23 Hz), 110.1, 52.4. **^19^F NMR** (376 MHz, CDCl_3_) δ: −112.7, −57.8 (3×F). **HRMS** (ESI) *m*/*z* calculated for C_18_H_13_O_3_N_2_F_4_^+^ [M+H]^+^ 381.0857, found 381.0859.

**1i**. 101 mg, 85% yield; White solid. m.p. = 153–155 °C. **^1^H NMR** (400 MHz, CDCl_3_) δ: 7.87 (s, 1H), 7.74–7.72 (m, 1H), 7.55 (d, *J* = 8.8 Hz, 2H), 7.38–7.33 (m, 5H), 3.85 (s, 3H). **^13^C NMR** (100 MHz, CDCl_3_) δ: 159.4, 150.7, 149.2 (q, *J* = 1.5 Hz), 138.5, 135.0, 134.5, 133.7, 130.2, 128.7, 127.7 (2×C), 126.0, 124.0, 121.2 (2×C), 120.5 (q, *J* = 256 Hz), 110.1, 52.4. **^19^F NMR** (376 MHz, CDCl_3_) δ: −57.8 (3×F). **HRMS** (ESI) *m*/*z* calculated for C_18_H_13_O_3_N_2_F_3_Cl^+^ [M+H]^+^ 397.0561, found 397.0562.

**1j**. 103 mg, 78% yield; White solid. m.p. = 165–167 °C. **^1^H NMR** (400 MHz, CDCl_3_) δ: 8.03 (t, *J* = 1.6 Hz, 1H), 7.77 (dt, *J* = 7.6, 1.2 Hz, 1H), 7.56 (d, *J* = 8.8 Hz, 2H), 7.49 (ddd, *J* = 8.0, 2.0, 1.2 Hz, 1H), 7.34 (d, *J* = 8.8 Hz, 2H), 7.33 (s, 1H), 7.30 (t, *J* = 8.0 Hz, 1H), 3.85 (s, 3H). **^13^C NMR** (100 MHz, CDCl_3_) δ: 159.4, 150.5, 149.3 (q, *J* = 1.9 Hz), 138.5, 134.5, 133.9, 131.6, 130.5, 128.9, 127.7 (2×C), 124.4, 123.1, 121.2 (2×C), 120.5 (q, *J* = 256 Hz), 110.1, 52.4. **^19^F NMR** (376 MHz, CDCl_3_) δ: −57.8 (3×F). **HRMS** (ESI) *m*/*z* calculated for C_18_H_13_O_3_N_2_F_3_Br^+^ [M+H]^+^ 441.0056, found 441.0061.

**1k**. 85 mg, 66% yield; White solid. m.p. = 174–176 °C. **^1^H NMR** (400 MHz, CDCl_3_) δ: 8.13 (s, 1H), 8.04 (d, *J* = 7.6 Hz, 1H), 7.62 (d, *J* = 7.6 Hz, 1H), 7.58–7.53 (m, 3H), 7.39 (s, 1H), 7.35 (d, *J* = 8.4 Hz, 2H), 3.86 (s, 3H). **^13^C NMR** (100 MHz, CDCl_3_) δ: 159.4, 150.6, 149.3, 138.4, 134.7, 132.8, 131.4 (q, *J* = 32.2 Hz), 129.5, 129.0, 127.8 (2×C), 125.3 (q, *J* = 3.8 Hz), 124.2 (q, *J* = 271 Hz), 122.7 (q, *J* = 3.7 Hz), 121.2 (2×C), 120.5 (q, *J* = 256 Hz), 110.1, 52.5. **^19^F NMR** (376 MHz, CDCl_3_) δ: −62.7 (3×F), −57.8 (3×F). **HRMS** (ESI) *m*/*z* calculated for C_19_H_13_O_3_N_2_F_6_^+^ [M+H]^+^ 431.0825, found 431.0827.

**1l**. 86 mg, 76% yield; White solid. m.p. = 87–89 °C. **^1^H NMR** (400 MHz, CDCl_3_) δ: 7.63–7.61 (m, 1H), 7.57 (d, *J* = 8.8 Hz, 2H), 7.33 (d, *J* = 8.8 Hz, 2H), 7.31–7.24 (m, 3H), 7.21 (s, 1H), 3.85 (s, 3H), 2.53 (s, 3H). **^13^C NMR** (100 MHz, CDCl_3_) δ: 159.7, 152.4, 149.0 (q, *J* = 2.0 Hz), 138.6, 136.3, 133.4, 131.6, 131.1, 129.4, 128.6, 127.6 (2×C), 126.2, 121.1 (2×C), 120.5 (q, *J* = 256 Hz), 113.1, 52.3, 21.4. **^19^F NMR** (376 MHz, CDCl_3_) δ: −57.8 (3×F). **HRMS** (ESI) *m*/*z* calculated for C_19_H_16_O_3_N_2_F_3_^+^ [M+H]^+^ 377.1108, found 377.1109.

**1m**. 101 mg, 88% yield; White solid. m.p. = 149–151 °C. **^1^H NMR** (400 MHz, CDCl_3_) δ: 8.06 (td, *J* = 7.6, 1.6 Hz, 1H), 7.56 (d, *J* = 8.8 Hz, 2H), 7.49 (d, *J* = 3.6 Hz, 1H), 7.37–7.31 (m, 3H), 7.23–7.15 (m, 2H), 3.85 (s, 3H). **^13^C NMR** (100 MHz, CDCl_3_) δ: 160.4 (d, *J* = 248 Hz), 159.6, 149.2 (q, *J* = 1.9 Hz), 146.7, 138.6, 134.1 (d, *J* = 2.0 Hz), 130.1 (d, *J* = 8.3 Hz), 128.5 (d, *J* = 3.3 Hz), 127.8 (2×C), 124.5 (d, *J* = 3.5 Hz), 121.2 (2×C), 120.5 (q, *J* = 256 Hz), 119.8 (d, *J* = 11.6 Hz), 116.3 (d, *J* = 21.9 Hz), 113.5 (d, *J* = 10.8 Hz), 52.4. **^19^F NMR** (376 MHz, CDCl_3_) δ: −115.8, −57.8 (3×F). **HRMS** (ESI) *m*/*z* calculated for C_18_H_13_O_3_N_2_F_4_^+^ [M+H]^+^ 381.0857, found 381.0859.

**1n**. 91 mg, 76% yield; White solid. m.p. = 79–81 °C. **^1^H NMR** (400 MHz, CDCl_3_) δ: 7.89–7.85 (m, 1H), 7.58 (s, 1H), 7.57 (d, *J* = 8.8 Hz, 2H), 7.50–7.47 (m, 1H), 7.36–7.29 (m, 4H), 3.85 (s, 3H). **^13^C NMR** (100 MHz, CDCl_3_) δ: 159.6, 149.7, 149.2 (q, *J* = 1.9 Hz), 138.5, 133.5, 132.5, 130.8, 130.7, 130.6, 129.8, 127.8 (2×C), 127.2, 121.2 (2×C), 120.5 (q, *J* = 256 Hz), 113.9, 52.4. **^19^F NMR** (376 MHz, CDCl_3_) δ: −57.8 (3×F). **HRMS** (ESI) *m*/*z* calculated for C_18_H_13_O_3_N_2_F_3_Cl^+^ [M+H]^+^ 397.0561, found 397.0565.

**1o**. 115 mg, 87% yield; White solid. m.p. = 136–138 °C. **^1^H NMR** (400 MHz, CDCl_3_) δ: 7.77 (dd, *J* = 7.6, 1.6 Hz, 1H), 7.69 (dd, *J* = 7.6, 1.2 Hz, 1H), 7.59–7.55 (m, 3H), 7.38 (td, *J* = 7.6, 1.2 Hz, 1H), 7.33 (d, *J* = 8.8 Hz, 2H), 7.24 (td, *J* = 7.6, 1.6 Hz, 1H), 3.85 (s, 3H). **^13^C NMR** (100 MHz, CDCl_3_) δ: 159.6, 151.1, 149.2 (q, *J* = 1.9 Hz), 138.5, 133.8, 133.3, 133.0, 131.2, 130.0, 127.8 (2×C), 127.7, 122.1, 121.1 (2×C), 120.5 (q, *J* = 256 Hz), 113.9, 52.4. **^19^F NMR** (376 MHz, CDCl_3_) δ: −57.8 (3×F). **HRMS** (ESI) *m*/*z* calculated for C_18_H_13_O_3_N_2_F_3_Br^+^ [M+H]^+^ 441.0056, found 441.0061.

**1p**. 93 mg, 72% yield; White solid. m.p. = 70–72 °C. **^1^H NMR** (400 MHz, CDCl_3_) δ: 7.79 (d, *J* = 7.6 Hz, 1H), 7.73 (d, *J* = 7.6 Hz, 1H), 7.63–7.50 (m, 4H), 7.33 (d, *J* = 8.8 Hz, 2H), 7.25 (d, *J* = 0.8 Hz, 1H), 3.85 (s, 3H). **^13^C NMR** (100 MHz, CDCl_3_) δ: 159.6, 150.2, 149.2 (q, *J* = 1.9 Hz), 138.4, 133.6, 132.0, 131.9, 131.5, 128.8, 128.6 (q, *J* = 30.5 Hz), 127.7 (2×C), 126.5 (q, *J* = 5.6 Hz), 124.2 (q, *J* = 272 Hz), 121.1 (2×C), 120.5 (q, *J* = 256 Hz), 113.6 (q, *J* = 3.6 Hz), 52.4. **^19^F NMR** (376 MHz, CDCl_3_) δ: −57.9 (3×F), −57.8 (3×F). **HRMS** (ESI) *m*/*z* calculated for C_19_H_13_O_3_N_2_F_6_^+^ [M+H]^+^ 431.0825, found 431.0828.

**1q**. 86 mg, 71% yield; Yellow oil. **^1^H NMR** (400 MHz, CDCl_3_) δ: 7.55 (d, *J* = 8.8 Hz, 2H), 7.31 (d, *J* = 8.8 Hz, 2H), 6.95 (s, 1H), 6.94 (s, 2H), 3.85 (s, 3H), 2.32 (s, 3H), 2.17 (s, 6H). **^13^C NMR** (100 MHz, CDCl_3_) δ: 159.8, 151.6, 149.0, 138.6, 138.2, 137.5 (2×C), 133.3, 129.2, 128.4 (2×C), 127.5 (2×C), 121.1 (2×C), 120.5 (q, *J* = 256 Hz), 114.2, 52.3, 21.3, 20.8 (2×C). **^19^F NMR** (376 MHz, CDCl_3_) δ: −57.9 (3×F). **HRMS** (ESI) *m*/*z* calculated for C_21_H_20_O_3_N_2_F_3_^+^ [M+H]^+^ 405.1421, found 405.1428.

**1r**. 73 mg, 67% yield; White solid. m.p. = 149–151 °C. **^1^H NMR** (400 MHz, CDCl_3_) δ: 8.66 (dd, *J* = 4.8, 0.8 Hz, 1H), 8.02 (d, *J* = 8.0 Hz, 1H), 7.75 (td, *J* = 7.6, 1.6 Hz, 1H), 7.68 (s, 1H), 7.56 (d, *J* = 8.8 Hz, 2H), 7.33 (d, *J* = 8.8 Hz, 2H), 7.27 (ddd, *J* = 7.6, 4.8, 1.2 Hz, 1H), 3.84 (s, 3H). **^13^C NMR** (100 MHz, CDCl_3_) δ: 159.6, 152.3, 150.9, 149.8, 149.2 (q, *J* = 1.9 Hz), 138.6, 136.9, 134.6, 127.8 (2×C), 123.4, 121.2 (2×C), 120.5 (q, *J* = 256 Hz), 120.4, 111.5, 52.4. **^19^F NMR** (376 MHz, CDCl_3_) δ: −57.8 (3×F). **HRMS** (ESI) *m*/*z* calculated for C_17_H_13_O_3_N_3_F_3_^+^ [M+H]^+^ 364.0904, found 364.0911.

**1s**. 101 mg, 81% yield; White solid. m.p. = 72–74 °C. **^1^H NMR** (400 MHz, CDCl_3_) δ: 8.50 (d, *J* = 7.6 Hz, 1H), 7.93–7.90 (m, 2H), 7.76 (d, *J* = 7.2 Hz, 1H), 7.64 (d, *J* = 8.8 Hz, 2H), 7.58–7.51 (m, 3H), 7.38 (s, 1H), 7.36 (d, *J* = 8.8 Hz, 2H), 3.88 (s, 3H). **^13^C NMR** (100 MHz, CDCl_3_) δ: 159.7, 152.0, 149.2, 138.7, 134.1, 133.7, 131.2, 129.8, 129.3, 128.6, 127.7 (2×C), 127.6, 126.9, 126.1, 125.7, 125.5, 121.1 (2×C), 120.5 (q, *J* = 256 Hz), 113.9, 52.4. **^19^F NMR** (376 MHz, CDCl_3_) δ: −57.8 (3×F). **HRMS** (ESI) *m*/*z* calculated for C_22_H_16_O_3_N_2_F_3_^+^ [M+H]^+^ 413.1108, found 413.1106.

**1t**. 78 mg, 79% yield; Yellow oil. **^1^H NMR** (400 MHz, CDCl_3_) δ: 7.47 (d, *J* = 8.8 Hz, 2H), 7.29 (d, *J* = 8.8 Hz, 2H), 6.84 (s, 1H), 3.80 (s, 3H), 2.67 (t, *J* = 7.6 Hz, 2H), 1.77–1.67 (m, 2H), 0.99 (t, *J* = 7.6 Hz, 3H). **^13^C NMR** (100 MHz, CDCl_3_) δ: 159.8, 154.1, 148.9 (q, *J* = 1.9 Hz), 138.7, 133.4, 127.6 (2×C), 121.1 (2×C), 120.5 (q, *J* = 256 Hz), 111.9, 52.2, 30.1, 22.8, 14.0. **^19^F NMR** (376 MHz, CDCl_3_) δ: −57.9 (3×F). **HRMS** (ESI) *m*/*z* calculated for C_15_H_16_O_3_N_2_F_3_^+^ [M+H]^+^ 329.1108, found 329.1110.

**1u**. 54 mg, 55% yield; Yellow oil. **^1^H NMR** (400 MHz, CDCl_3_) δ: 7.47 (d, *J* = 8.8 Hz, 2H), 7.29 (d, *J* = 8.8 Hz, 2H), 6.87 (s, 1H), 3.80 (s, 3H), 3.11–3.00 (m, 1H), 1.31 (d, *J* = 6.8 Hz, 6H). **^13^C NMR** (100 MHz, CDCl_3_) δ: 159.9, 159.8, 148.9, 138.8, 133.3, 127.6 (2×C), 121.1 (2×C), 120.5 (q, *J* = 256 Hz), 110.1, 52.2, 27.9, 22.8 (2×C). **^19^F NMR** (376 MHz, CDCl_3_) δ: −57.9 (3×F). **HRMS** (ESI) *m*/*z* calculated for C_15_H_16_O_3_N_2_F_3_^+^ [M+H]^+^ 329.1108, found 329.1113.

**1v**. 92 mg, 83% yield; Yellow solid. m.p. = 89–91 °C. **^1^H NMR** (400 MHz, CDCl_3_) δ: 7.47 (d, *J* = 8.8 Hz, 2H), 7.28 (d, *J* = 8.8 Hz, 2H), 6.85 (s, 1H), 3.80 (s, 3H), 2.74–2.67 (m, 1H), 2.03–2.00 (m, 2H), 1.84–1.80 (m, 2H), 1.75–1.71 (m, 1H), 1.50–1.33 (m, 4H), 1.31–1.21 (m, 1H). **^13^C NMR** (100 MHz, CDCl_3_) δ: 159.8, 159.0, 148.8, 138.8, 133.2, 127.6 (2×C), 121.1 (2×C), 120.5 (q, *J* = 256 Hz), 110.3, 52.2, 37.4, 33.2 (2×C), 26.3 (2×C), 26.1. **^19^F NMR** (376 MHz, CDCl_3_) δ: −57.9 (3×F). **HRMS** (ESI) *m*/*z* calculated for C_18_H_20_O_3_N_2_F_3_^+^ [M+H]^+^ 369.1421, found 369.1425.

**1w**. 60 mg, 56% yield; Light yellow solid. m.p. = 73–75 °C. **^1^H NMR** (400 MHz, CDCl_3_) δ: 7.52 (s, 1H), 7.50 (d, *J* = 8.8 Hz, 2H), 7.32 (d, *J* = 8.8 Hz, 2H), 4.44 (q, *J* = 7.2 Hz, 2H), 3.83 (s, 3H), 1.41 (t, *J* = 7.2 Hz, 3H). **^13^C NMR** (100 MHz, CDCl_3_) δ: 161.5, 158.9, 149.7, 144.3, 138.0, 134.6, 128.0 (2×C), 121.1 (2×C), 120.5 (q, *J* = 256 Hz), 115.1, 61.7, 52.6, 14.5. **^19^F NMR** (376 MHz, CDCl_3_) δ: −57.9 (3×F). **HRMS** (ESI) *m*/*z* calculated for C_15_H_14_O_5_N_2_F_3_^+^ [M+H]^+^ 359.0849, found 359.0855.

**2a [27]**. 61mg, 52% yield; Yellow solid. **^1^H NMR** (400 MHz, CDCl_3_) δ: 8.22 (d, *J* = 9.2 Hz, 2H), 7.80 (d, *J* = 8.8 Hz, 2H), 7.44 (d, *J* = 8.8 Hz, 2H), 7.32 (d, *J* = 8.4 Hz, 2H), 3.24–3.22 (m, 2H), 3.16–3.13 (m, 2H). **^13^C NMR** (100 MHz, CDCl_3_) δ: 189.2, 147.7, 146.7, 145.4, 144.8, 137.7, 134.6, 130.4, 129.3 (2×C), 127.3 (2×C), 121.9 (2×C), 121.4 (2×C), 120.6 (q, *J* = 256 Hz), 43.9, 19.8.

**2b [27]**. 65 mg, 53% yield; White solid. **^1^H NMR** (400 MHz, CDCl_3_) δ: 7.71 (d, *J* = 8.8 Hz, 2H), 7.60 (d, *J* = 9.2 Hz, 2H), 7.43 (d, *J* = 8.8 Hz, 2H), 7.31 (d, *J* = 8.8 Hz, 2H), 3.03 (t, *J* = 6.0 Hz, 2H), 2.65 (dd, *J* = 7.2, 6.0 Hz, 2H), 2.26–2.20 (m, 2H). **^13^C NMR** (100 MHz, CDCl_3_) δ: 188.4, 148.8, 148.3, 138.4, 136.0, 134.4, 130.8, 129.3, 129.1 (2×C), 128.5 (2×C), 126.9 (2×C), 121.1 (2×C), 120.5 (q, *J* = 256 Hz), 39.6, 24.7, 22.8.

**2c [27]**. 93 mg, 68% yield; Yellow solid. **^1^H NMR** (400 MHz, CDCl_3_) δ: 7.69 (d, *J* = 8.8 Hz, 2H), 7.58 (d, *J* = 9.2 Hz, 2H), 7.41 (d, *J* = 8.8 Hz, 2H), 7.37 (d, *J* = 8.8 Hz, 2H), 3.88 (s, 3H), 3.84 (s, 3H). **^13^C NMR** (100 MHz, CDCl_3_) δ: 163.3, 160.4, 151.2, 149.5, 137.3, 137.0, 135.3, 130.2 (2×C), 129.7, 128.7 (2×C), 126.4 (2×C), 121.7 (2×C), 120.5 (q, *J* = 257 Hz), 114.7, 53.5, 52.5.

### 4.3. Bioassays

The antifungal activity of all synthesized compounds **1a**–**1w** and **2a**–**2c** was tested against six plant pathogenic fungi, namely *Botrytis cinerea*, *Magnaporthe oryzae*, *Pythium aphanidermatum*, *Fusarium graminearum*, *Colletotrichum micotianae,* and *Valsa mali*, by the poison plate technique according to our previous report [28,29].

Compounds were dissolved in DMSO (0.5 mL) before mixing with potato dextrose agar (PDA 99.5 mL) medium. The final concentrations of compounds **1**–**2** in the medium were fixed at 100 µg/mL. Three kinds of fungi were incubated in PDA at 25 °C for five days to produce new mycelium for the antifungal assay, then a mycelia disk of approximately 0.45 cm diameter cut from the culture medium was picked up with a sterilized inoculation needle and inoculated in the center of the PDA plate. The inoculated plates were incubated at 25 °C for five days. DMSO in sterilized distilled water served as the control, while pyraclostrobin was used as a positive control for each treatment, with three replicates carried out. The radial growth of the fungal colonies was measured on the sixth day, and the data were statistically analyzed. The relative control efficacy of compounds compared to the blank assay was calculated using the following equation: I (%) = [(CK – PT/CK)] × 100%, where I is the relative control efficacy, CK is the average disease index during the blank assay, and PT is the average disease index after treatment during testing. The in vitro inhibiting effects of the test compounds on the fungi were calculated by the formula CV = (A − B)/A, where A represents the diameter of fungi growth on untreated PDA, B represents the diameter of fungi on treated PDA, and CV represents the rate of inhibition. All of the strains were conserved in the Key Laboratory of Biopesticide and Chemical Biology, Ministry of Education, Fujian Agriculture and Forestry University (Fuzhou, China).

Based on the results of in vitro antifungal activity, the more active compounds were selected to determine their median effective concentration (EC_50_) according to the same method described above. The stock solution was mixed with the autoclaved PDA medium to prepare a set of mediums containing 100, 50, 25, 12.5, 6.25, and 3.125 μg/mL of the tested compound. Similarly, 0.5% DMSO in the culture medium was used as a blank control. Each test was performed in triplicate. EC_50_ values and their confidence intervals at 95% probability (95% CI) were calculated by using the basic EC_50_ program in SPSS version 22.0.

## 5. Conclusions

In conclusion, aiming to discover and develop low-cost and versatile antifungal agents, a series of pyrazole compounds bearing aryl OCF_3_ (26 examples) were well-designed and prepared by one-pot three-component reactions, and their structures were characterized by ^1^H NMR, ^13^C NMR, and HRMS. The approach was efficient for implementing structural modification by simply changing reactants. Their in vitro antifungal activity against six plant pathogenic fungi was evaluated systematically. Most of the synthesized compounds showed growth inhibition activity against all the tested fungi, and some of them were comparable with pyraclostrobin. Among all the tested compounds, **1t** and **1v** exhibited higher activity against all the tested fungi, and **1v** displayed the highest activity against *F. graminearum* with an EC_50_ value of 0.0530 μM. **1t** and **1v** possessed great potential to be developed as new antifungal agents for plant pathogenic fungi, and the presence of ester groups offered a great chemical space for further elaborations. Structure–activity relationship analysis showed that, with respect to the R_1_ substituent, the straight chain or cycloalkyl ring moiety was a key structural moiety for the activity. Additionally, the R_3_ substituent on the pyrazole ring could have significant effects on the activity. We obtained simple and easy-to-access pyrazoles with potent antifungal activity, which are ready for further elaboration to serve as a pharmacophore. Furthermore, the work presented here was the first step for our unique compounds combining pyrazole and aryl OCF_3_, and further structure expeditions and bioactivity evaluations are ongoing and will be reported in due course.

## Figures and Tables

**Table 1:** Preliminary in vitro antifungal activity of compounds against six fungi at 100 μg/mL.

Compounds	Average Inhibition Rate ± SD (%) (*n* = 3)					
No.	*B. cinerea*	*M. oryzae*	*P. aphanidermatum*	*F. graminearum*	*C. micotianae*	*V. mali*
**1a**	5.74 ± 1.13	2.78 ± 1.04	5.81 ± 2.83	9.65 ± 0.93	2.45 ± 1.52	9.59 ± 0.96
**1b**	7.23 ± 0.68	1.44 ± 1.20	10.47 ± 2.64	17.05 ± 3.38	8.48 ± 0.80	3.99 ± 2.94
**1c**	4.80 ± 0.97	0.97 ± 1.32	5.65 ± 1.05	25.61 ± 1.20	0.61 ± 2.38	0.97 ± 1.60
**1d**	4.80 ± 0.58	4.34 ± 0.95	9.84 ± 0.93	1.71 ± 0.59	0.41 ± 0.65	2.42 ± 0.84
**1e**	13.89 ± 0.76	8.73 ± 1.26	13.27 ± 1.06	17.71 ± 2.06	13.34 ± 5.08	16.64 ± 1.24
**1f**	22.32 ± 1.31	4.27 ± 1.30	5.39 ± 3.35	3.05 ± 0.71	7.51 ± 0.80	6.06 ± 0.68
**1g**	1.16 ± 0.61	12.00 ± 3.52	8.56 ± 0.84	20.41 ± 0.68	10.60 ± 1.20	3.79 ± 0.62
**1h**	5.25 ± 3.38	5.56 ± 1.55	3.63 ± 0.84	17.95 ± 7.95	0	4.92 ± 3.34
**1i**	11.52 ± 0.88	5.27 ± 1.29	4.80 ± 1.01	0.44 ± 0.72	0	6.08 ± 2.82
**1j**	9.59 ± 2.97	2.15 ± 0.94	1.25 ± 1.27	0.96 ± 1.02	0.41 ± 3.64	9.27 ± 1.60
**1k**	30.30 ± 0.67	24.24 ± 1.27	26.33 ± 0.59	18.43 ± 4.92	34.4 ± 0.82	24.92 ± 0.60
**1l**	3.78 ± 0.47	6.37 ± 1.04	11.42 ± 0.79	9.14 ± 1.28	7.70 ± 0.91	3.03 ± 1.21
**1m**	8.20 ± 1.21	4.64 ± 0.68	5.77 ± 1.10	21.36 ± 0.86	5.67 ± 1.65	4.59 ± 0.59
**1n**	2.88 ± 0.45	0	8.55 ± 1.17	6.08 ± 3.38	1.79 ± 1.21	4.88 ± 0.94
**1o**	5.25 ± 0.80	0	9.07 ± 3.73	0	0	9.35 ± 1.27
**1p**	31.29 ± 3.01	23.09 ± 0.77	27.24 ± 1.47	39.75 ± 0.92	33.05 ± 0.64	21.81 ± 0.98
**1q**	13.86 ± 0.59	9.24 ± 0.58	13.72 ± 8.08	33.19 ± 2.83	14.28 ± 0.64	3.39 ± 0.47
**1r**	4.15 ± 0.99	3.54 ± 2.85	3.83 ± 4.20	20.85 ± 1.18	15.24 ± 1.30	8.26 ± 1.32
**1s**	6.32 ± 3.95	6.37 ± 4.76	2.70 ± 0.82	16.38 ± 0.71	10.8 ± 0.72	0
**1t**	29.18 ± 1.34	40.06 ± 0.68	29.82 ± 0.74	65.11 ± 0.61	47.56 ± 4.43	28.14 ± 0.62
**1u**	7.91 ± 1.35	5.96 ± 0.71	4.48 ± 0.57	18.83 ± 0.53	18.54 ± 2.54	8.80 ± 2.28
**1v**	37.61 ± 3.33	35.33 ± 0.62	38.51 ± 1.31	81.22 ± 0.60	56.03 ± 0.80	41.22 ± 0.65
**1w**	13.70 ± 0.83	11.59 ± 0.77	15.23 ± 0.59	7.79 ± 0.78	12.93 ± 4.26	6.73 ± 0.96
**2a**	26.55 ± 2.07	23.27 ± 0.96	22.42 ± 1.08	45.54 ± 4.62	36.81 ± 5.90	23.57 ± 0.44
**2b**	13.57 ± 0.87	14.88 ± 2.41	14.03 ± 1.13	43.83 ± 3.90	40.39 ± 2.46	20.39 ± 1.61
**2c**	16.92 ± 0.88	16.64 ± 0.45	10.63 ± 1.10	32.04 ± 0.86	46.01 ± 1.08	15.41 ± 3.21
Pyraclostrobin	77.57 ± 0.93	77.53 ± 0.62	75.57 ± 0.59	80.14 ± 1.16	69.62 ± 0.61	74.38 ± 0.72

**Table 2:** EC_50_ values of **1v** and **1t** against corresponding strains of fungi (µM).

**Compounds**		EC_50_ (95% CI *)	EC_50_ (95% CI *)
**No.**	**R**	*F. graminearum*	*C. micotianae*
**1v**	cyclohexyl	0.0530 (0.0409–0.0669)	0.1430 (0.1152–0.1953)
**1t**	*n*-propyl	0.0735 (0.0506–0.1053)	-
Pyraclostrobin		0.0112 (0.0026–0.0211)	0.0352 (0.0165–0.0551)

* 95% CI, confidence intervals at 95% probability.

## Data Availability

Data are contained within the article and Supporting Information.

## Supplementary Materials

The following supporting information can be downloaded at: https://www.mdpi.com/article/10.3390/molecules28176279/s1, Copies of the ^1^H and ^13^C spectra of new compounds.

## Sample Availability

Samples of compounds **1** and **2** are available from the authors.
