# Supplementary material for: Design, Synthesis and Antifungal Activities of Novel Pyrazole Analogues Containing the Aryl Trifluoromethoxy Group
Source: Molecules. 2023 Aug 28;28(17):6279. doi: 10.3390/molecules28176279 (PMC10488855; doi:10.3390/molecules28176279)
Supplement: Supplementary file 1 [file molecules-28-06279-s001.zip › molecules-2570809-supplementary.pdf]

Supporting Information

Design, Synthesis and Antifungal Activities of Novel Pyrazole Analogues Containing the Aryl Trifluoromethoxy Group

Tongchao Zhao, Yuyao Sun, Yufei Meng, Lifang Liu, Jingwen Dai, Guoan Yan, Xiaohong Pan, Xiong Guan, Liyan Song * and Ran Lin *

Key Laboratory of Biopesticide and Chemical Biology, Ministry of Education, College of Plant Protection, Fujian Agriculture and Forestry University, Fuzhou 350002, China

songliyan@fafu.edu.cn (L.S.); linran@fafu.edu.cn (R.L.)

Table of Content

^{1}H and ^{13}C NMR Spectra of New Compounds **S-2**

Citation: Zhao, T.; Sun, Y.; Meng, Y.; Liu, L.; Dai, J.; Yan, G.; Pan, X.; Guan, X.; Song, L.; Lin, R. Design, synthesis and antifungal activities of novel pyrazole analogues containing the aryl trifluoromethoxy group. *Molecules* **2023**, *28*, 6279. https://doi.org/10.3390/molecules28176279

Academic Editor: Josef Jampilek

Received: 5 August 2023
Revised: 18 August 2023
Accepted: 22 August 2023
Published: 28 August 2023

Copyright: © 2023 by the authors. Submitted for possible open access publication under the terms and conditions of the Creative Commons Attribution (CC BY) license (https://creativecommons.org/licenses/by/4.0/).

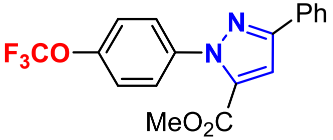

1a

^1H NMR (400 MHz, $CDCl_3$)

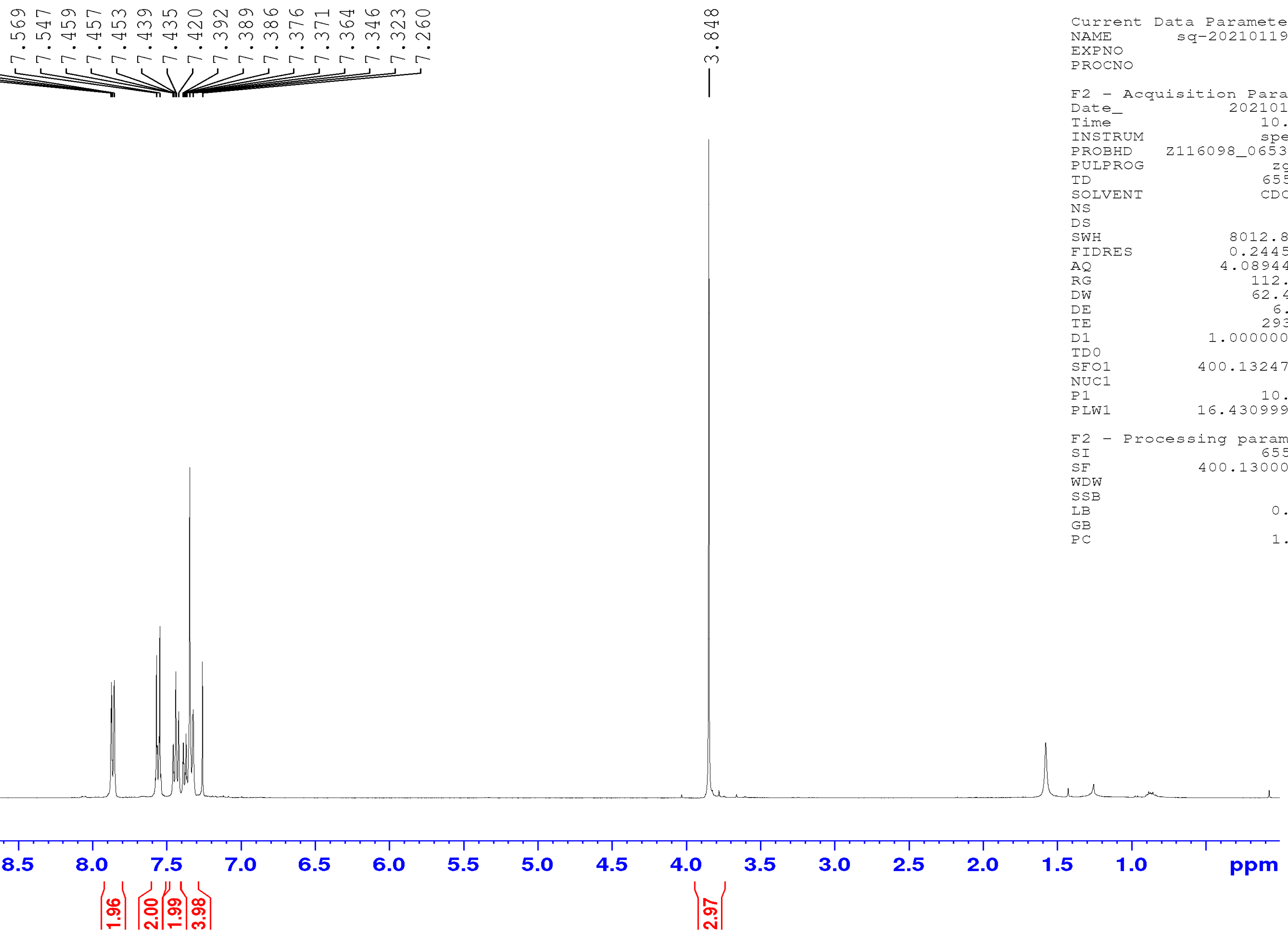


```
Current Data Parameters
NAME        sq-20210119-3
EXPNO                   1
PROCNO                  1

F2 - Acquisition Parameters
Date_            20210119
Time                10.49 h
INSTRUM             spect
PROBHD    Z116098_0653 (
PULPROG              zg30
TD                  65536
SOLVENT             CDCl3
NS                      8
DS                      2
SWH              8012.820 Hz
FIDRES           0.244532 Hz
AQ              4.0894465 sec
RG                 112.15
DW                 62.400 usec
DE                   6.50 usec
TE                  293.8 K
D1             1.00000000 sec
TD0                     1
SFO1          400.1324708 MHz
NUC1                   1H
P1                  10.00 usec
PLW1          16.43099976 W

F2 - Processing parameters
SI                  65536
SF            400.1300098 MHz
WDW                    EM
SSB                     0
LB                   0.30 Hz
GB                      0
PC                   1.00
```

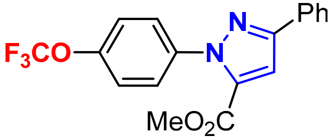

1a

^{13}C NMR (100 MHz, $CDCl_3$)

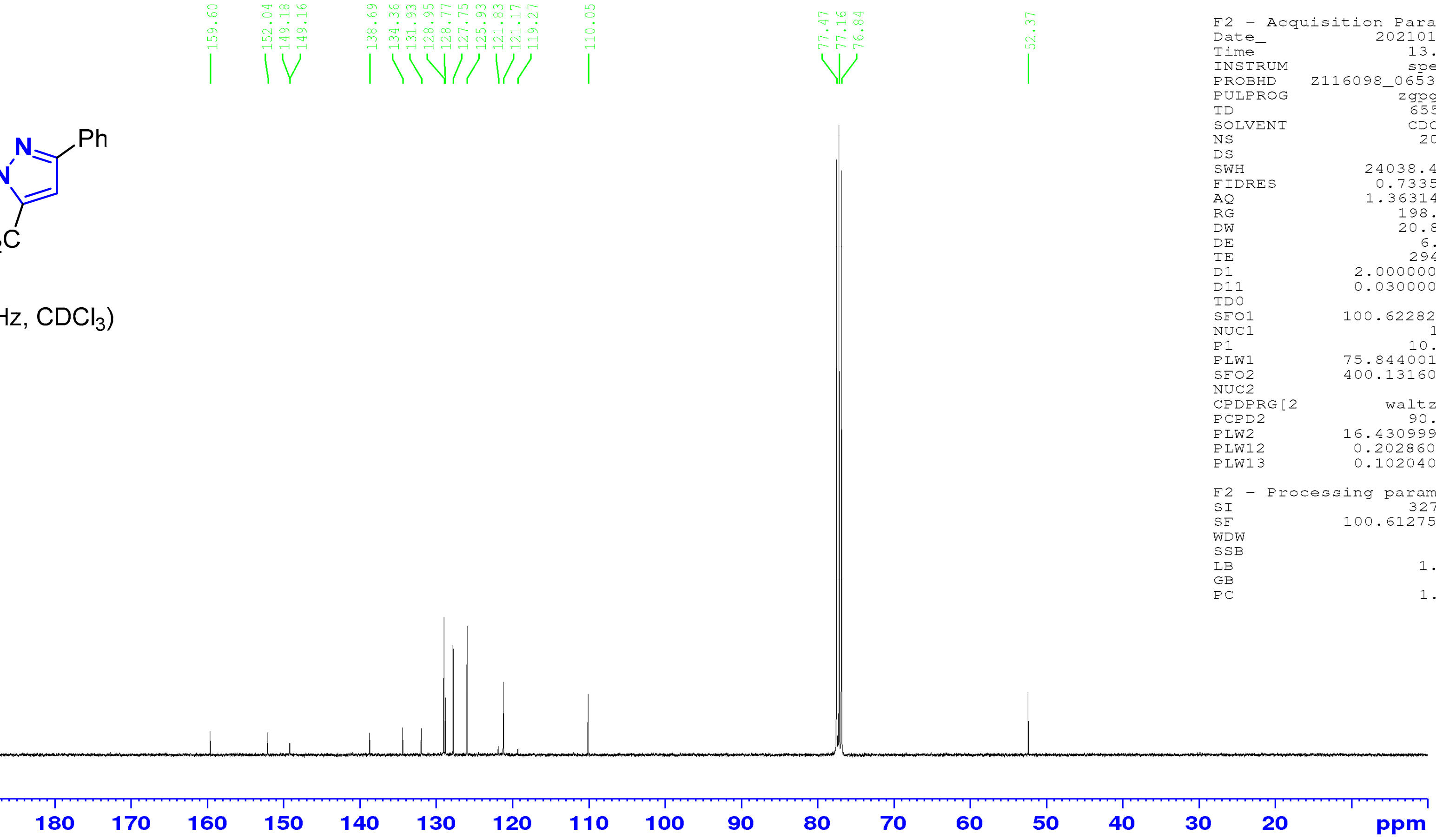


```
Current Data Parameters
NAME        sq-20210119-3
EXPNO                   2
PROCNO                  1

F2 - Acquisition Parameters
Date_            20210120
Time                13.31 h
INSTRUM             spect
PROBHD    Z116098_0653 (
PULPROG           zgpg30
TD                 65536
SOLVENT            CDCl3
NS                  2048
DS                     4
SWH            24038.461 Hz
FIDRES          0.733596 Hz
AQ             1.3631488 sec
RG                198.36
DW                20.800 usec
DE                  6.50 usec
TE                 294.5 K
D1            2.00000000 sec
D11           0.03000000 sec
TD0                    1
SFO1         100.6228298 MHz
NUC1                 13C
P1                 10.00 usec
PLW1         75.84400177 W
SFO2         400.1316005 MHz
NUC2                  1H
CPDPRG[2         waltz16
PCPD2              90.00 usec
PLW2         16.43099976 W
PLW12         0.20286000 W
PLW13         0.10204000 W

F2 - Processing parameters
SI                 32768
SF           100.6127564 MHz
WDW                   EM
SSB                    0
LB                  1.00 Hz
GB                     0
PC                  1.40
```

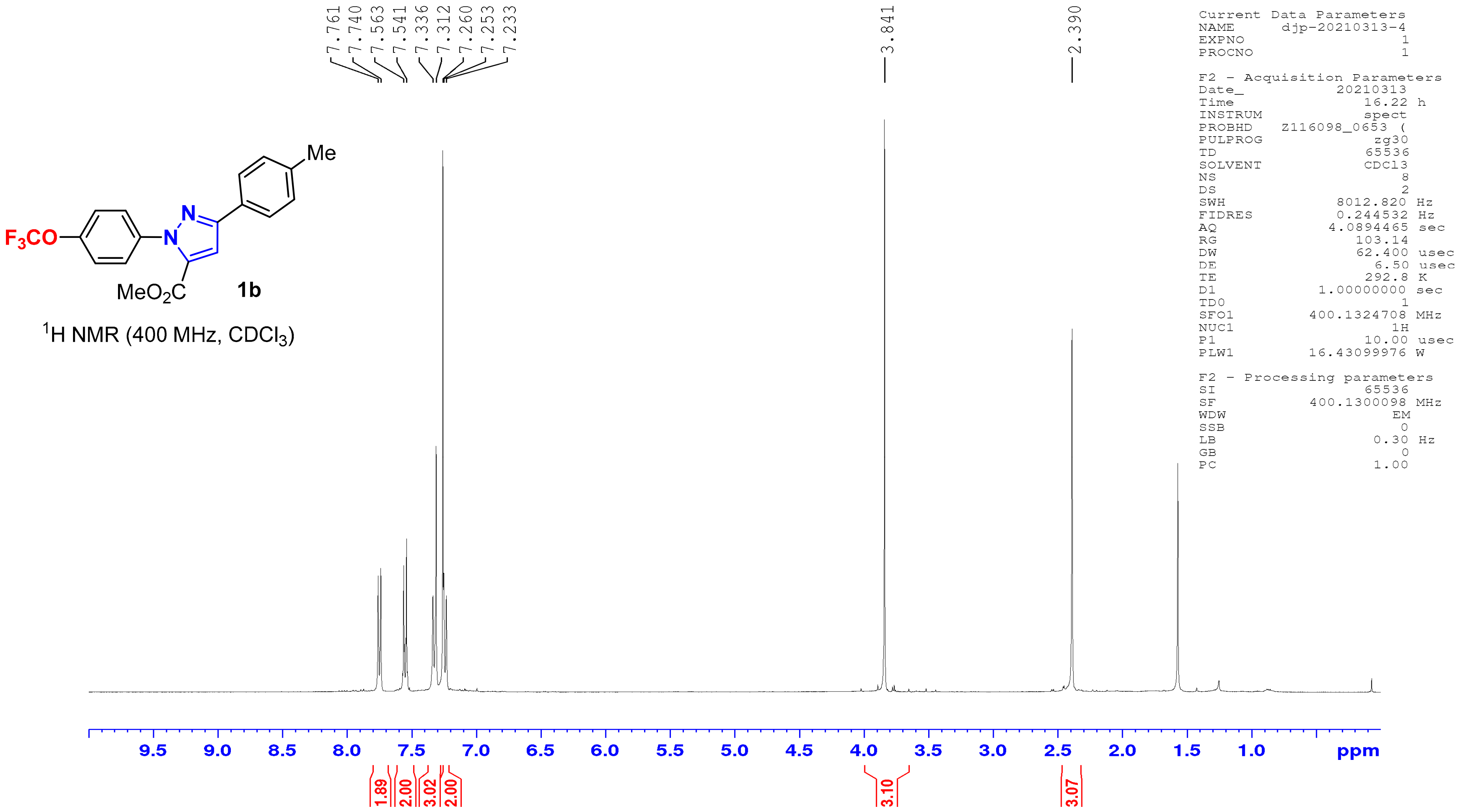
7.761
7.740
7.563
7.541
7.336
7.312
7.260
7.253
7.233
3.841
2.390
Me
N
N
F3CO
MeO2C
1b
1H NMR (400 MHz, CDCl3)
Current Data Parameters
NAME djp-20210313-4
EXPNO 1
PROCNO 1
F2 - Acquisition Parameters
Date_ 20210313
Time 16.22 h
INSTRUM spect
PROBHD Z116098_0653 (
PULPROG zg30
TD 65536
SOLVENT CDCl3
NS 8
DS 2
SWH 8012.820 Hz
FIDRES 0.244532 Hz
AQ 4.0894465 sec
RG 103.14
DW 62.400 usec
DE 6.50 usec
TE 292.8 K
D1 1.00000000 sec
TD0 1
SFO1 400.1324708 MHz
NUC1 1H
P1 10.00 usec
PLW1 16.43099976 W
F2 - Processing parameters
SI 65536
SF 400.1300098 MHz
WDW EM
SSB 0
LB 0.30 Hz
GB 0
PC 1.00
9.5
9.0
8.5
8.0
7.5
7.0
6.5
6.0
5.5
5.0
4.5
4.0
3.5
3.0
2.5
2.0
1.5
1.0
ppm
1.89
2.00
3.02
2.00
3.10
3.07

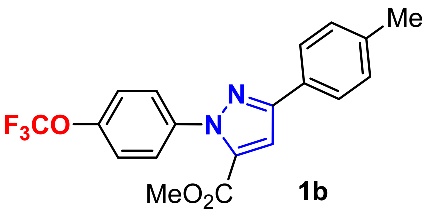

1b

^{13}C NMR (100 MHz, $CDCl_3$)

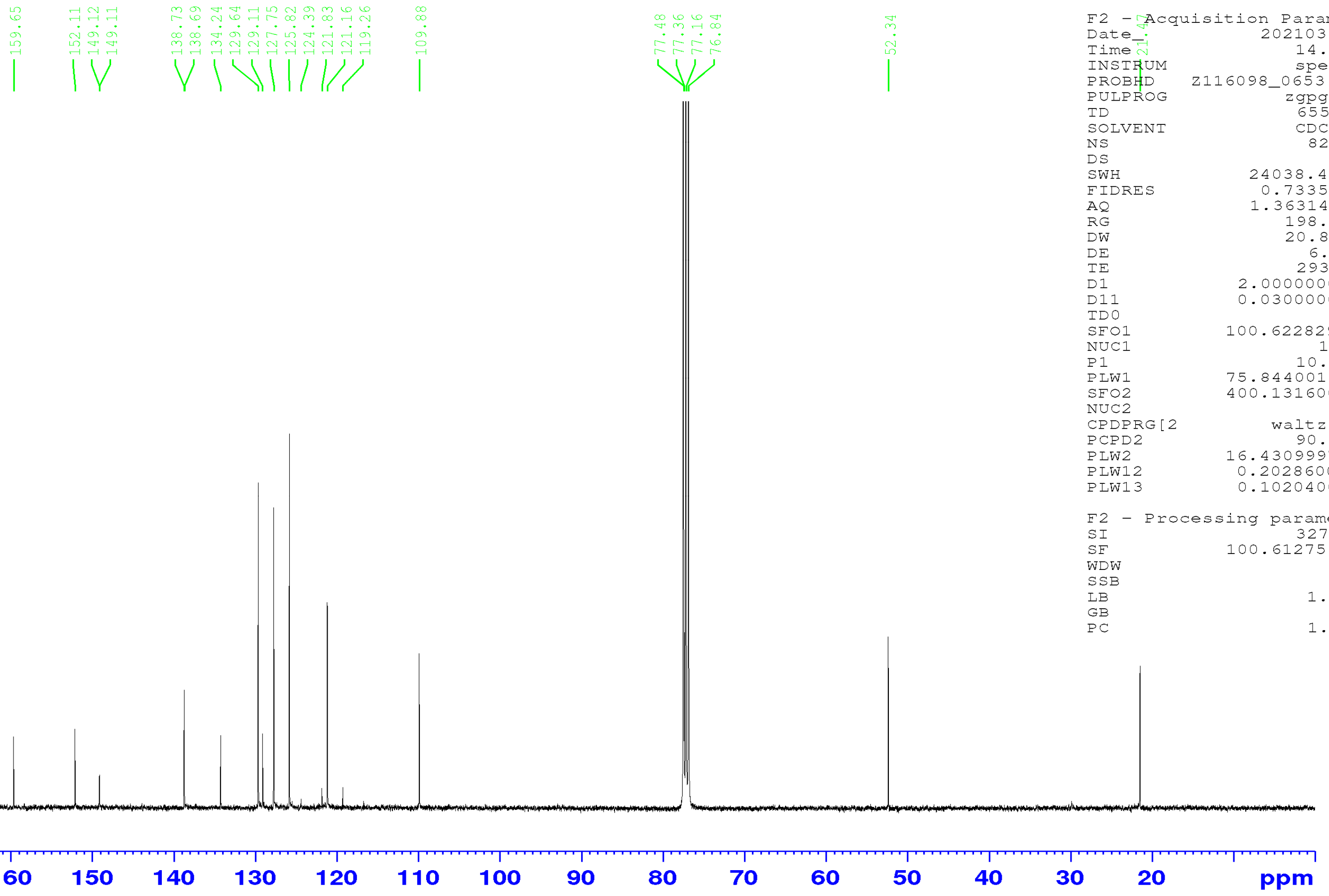


```
Current Data Parameters
NAME      djp-20210313-4
EXPNO                  2
PROCNO                 1

F2 - Acquisition Parameters
Date_           20210314
Time               14.50 h
INSTRUM            spect
PROBHD   Z116098_0653 (
PULPROG           zgpg30
TD                 65536
SOLVENT            CDCl3
NS                  8288
DS                     4
SWH            24038.461 Hz
FIDRES          0.733596 Hz
AQ             1.3631488 sec
RG                198.36
DW                20.800 usec
DE                  6.50 usec
TE                 293.5 K
D1            2.00000000 sec
D11           0.03000000 sec
TD0                    1
SFO1         100.6228298 MHz
NUC1                 13C
P1                 10.00 usec
PLW1         75.84400177 W
SFO2         400.1316005 MHz
NUC2                  1H
CPDPRG[2         waltz16
PCPD2              90.00 usec
PLW2         16.43099976 W
PLW12         0.20286000 W
PLW13         0.10204000 W

F2 - Processing parameters
SI                 32768
SF           100.6127565 MHz
WDW                   EM
SSB                    0
LB                  1.00 Hz
GB                     0
PC                  1.40
```

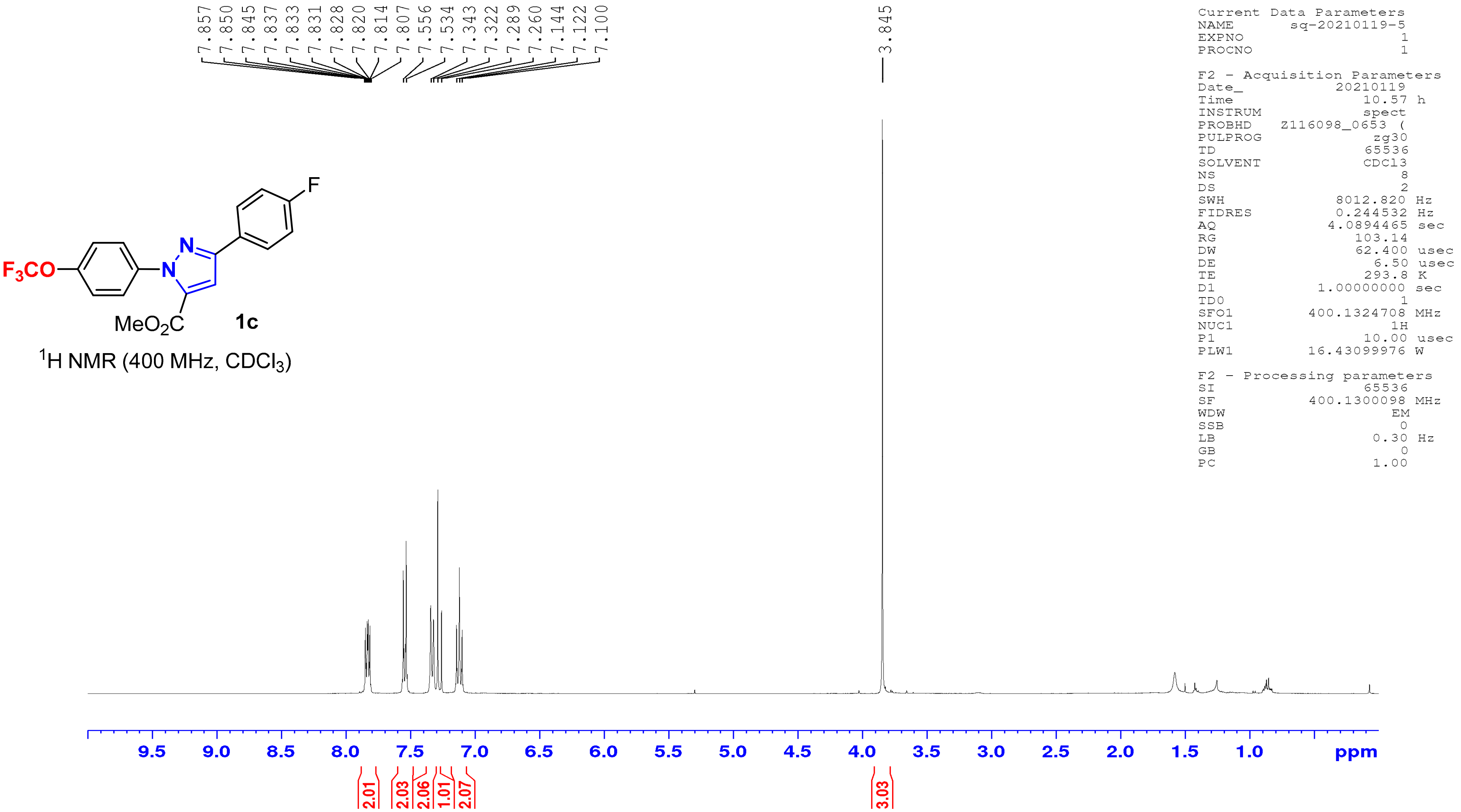

1c

^{1}H NMR (400 MHz, $CDCl_3$)

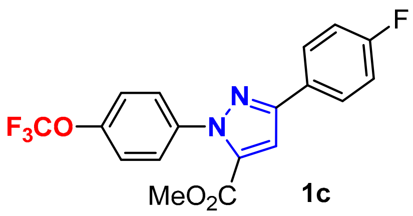

1c

^{13}C NMR (100 MHz, $CDCl_3$)

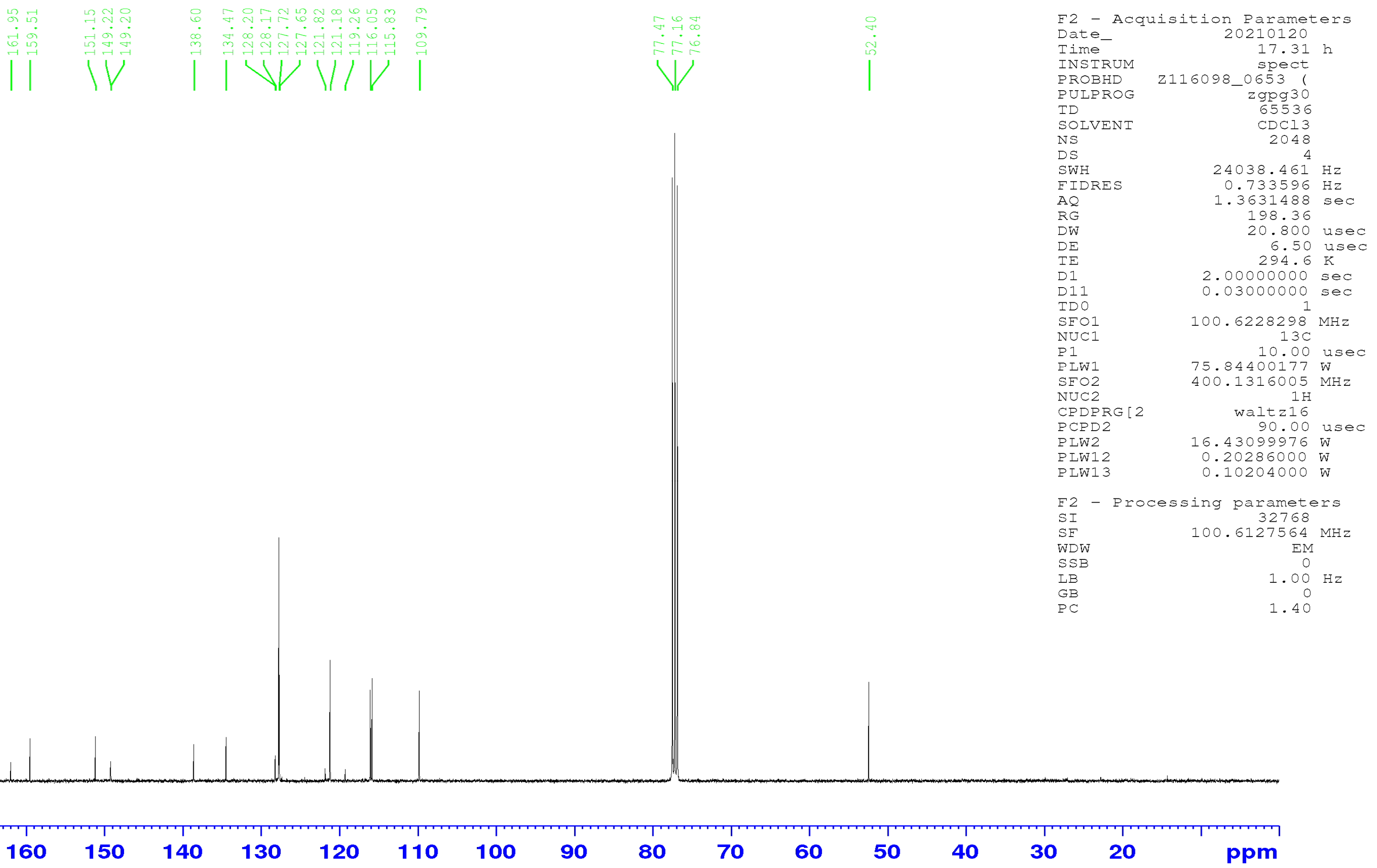

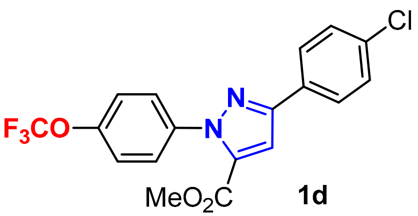

1d

^{1}H NMR (400 MHz, $CDCl_3$)

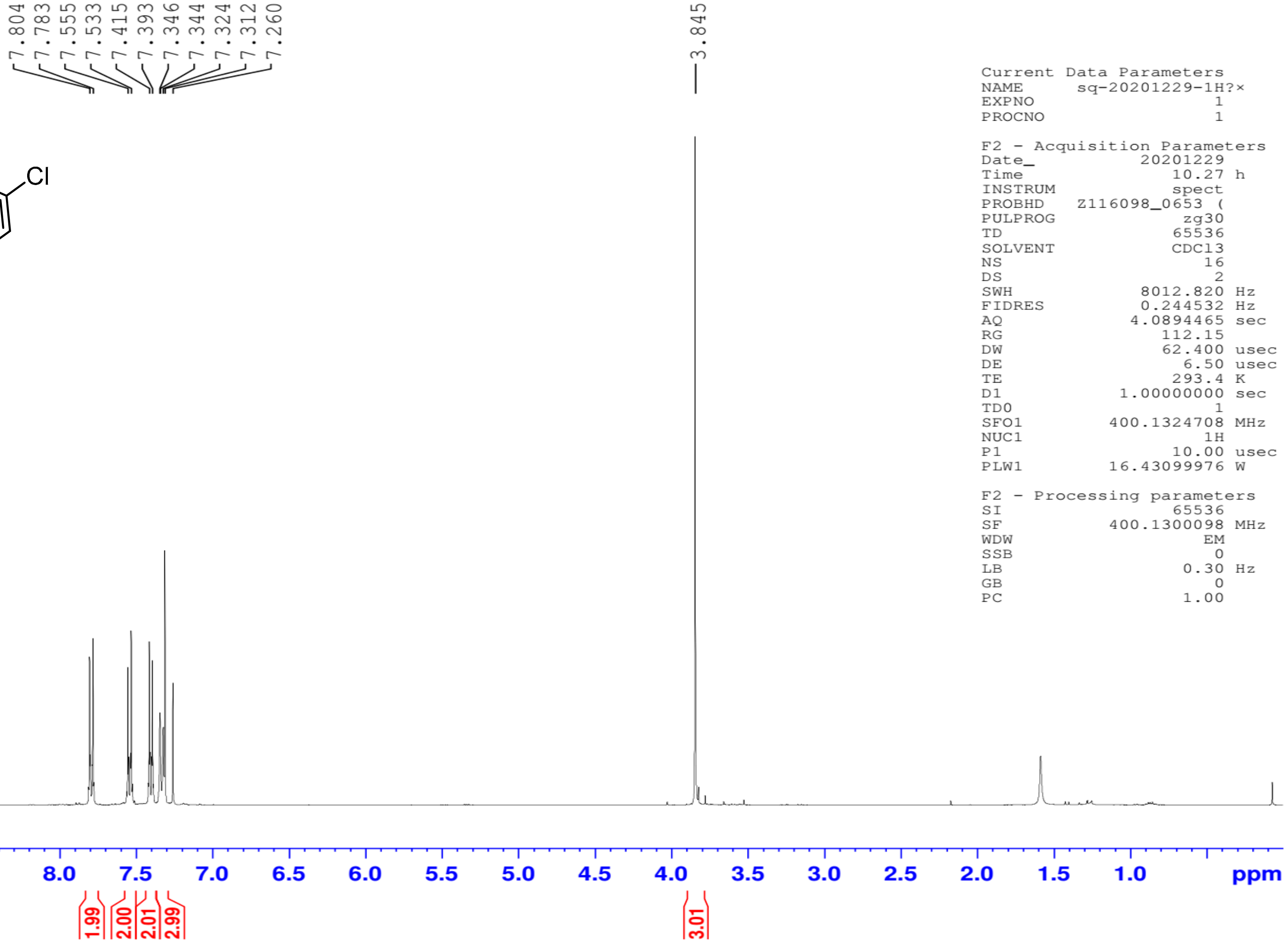

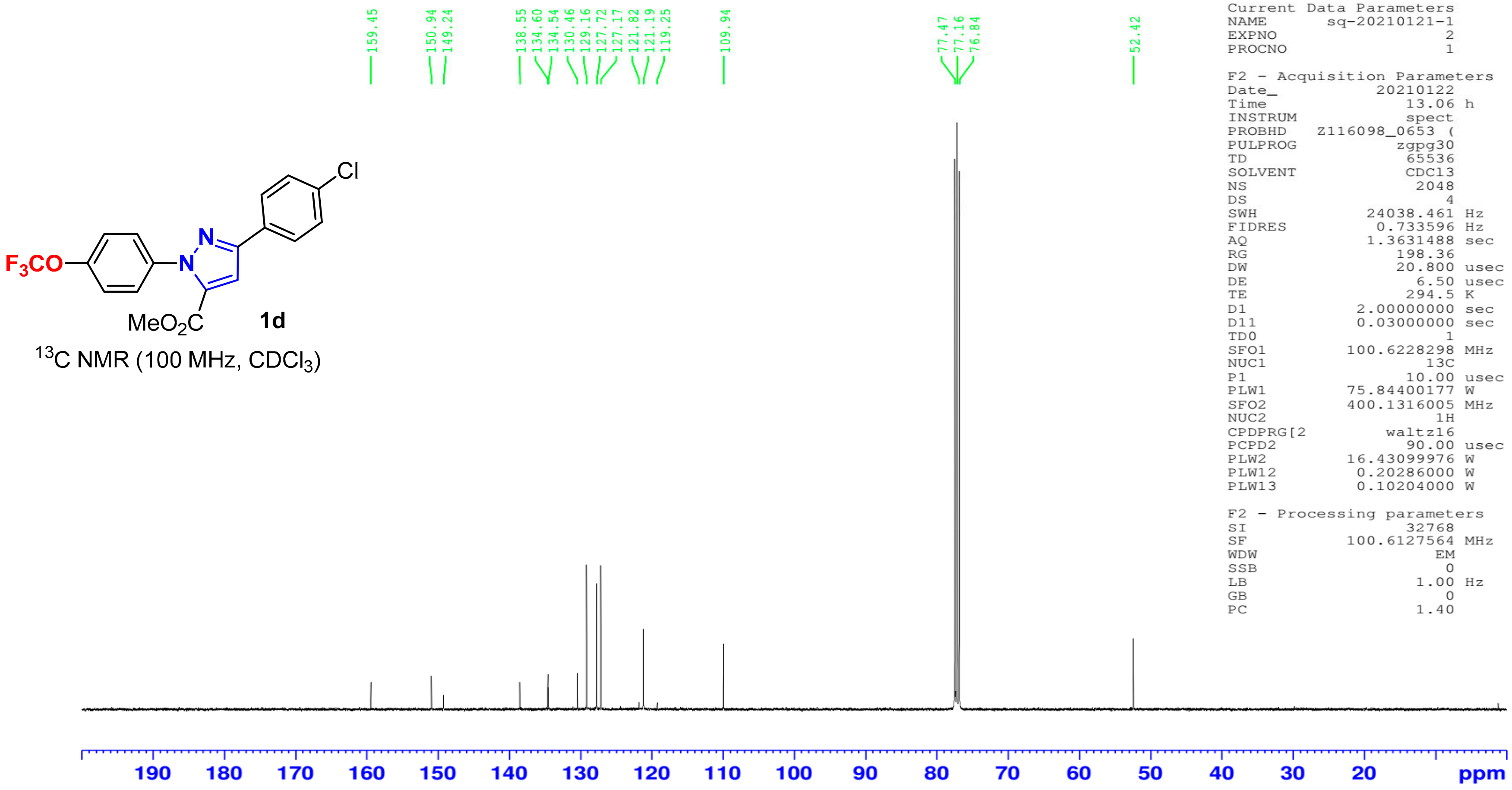
F3CO
N
N
Cl
MeO2C
1d
13C NMR (100 MHz, CDCl3)
159.45
150.94
149.24
138.55
134.60
134.54
130.46
129.16
127.72
127.17
121.82
121.19
119.25
109.94
77.47
77.16
76.84
52.42
Current Data Parameters
NAME sq-20210121-1
EXPNO 2
PROCNO 1
F2 - Acquisition Parameters
Date_ 20210122
Time 13.06 h
INSTRUM spect
PROBHD Z116098_0653 (
PULPROG zgpg30
TD 65536
SOLVENT CDCl3
NS 2048
DS 4
SWH 24038.461 Hz
FIDRES 0.733596 Hz
AQ 1.3631488 sec
RG 198.36
DW 20.800 usec
DE 6.50 usec
TE 294.5 K
D1 2.00000000 sec
D11 0.03000000 sec
TD0 1
SFO1 100.6228298 MHz
NUC1 13C
P1 10.00 usec
PLW1 75.84400177 W
SFO2 400.1316005 MHz
NUC2 1H
CPDPRG[2 waltz16
PCPD2 90.00 usec
PLW2 16.43099976 W
PLW12 0.20286000 W
PLW13 0.10204000 W
F2 - Processing parameters
SI 32768
SF 100.6127564 MHz
WDW EM
SSB 0
LB 1.00 Hz
GB 0
PC 1.40
190 180 170 160 150 140 130 120 110 100 90 80 70 60 50 40 30 20 ppm

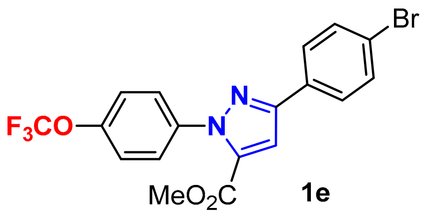

1e

^{1}H NMR (400 MHz, $CDCl_3$)

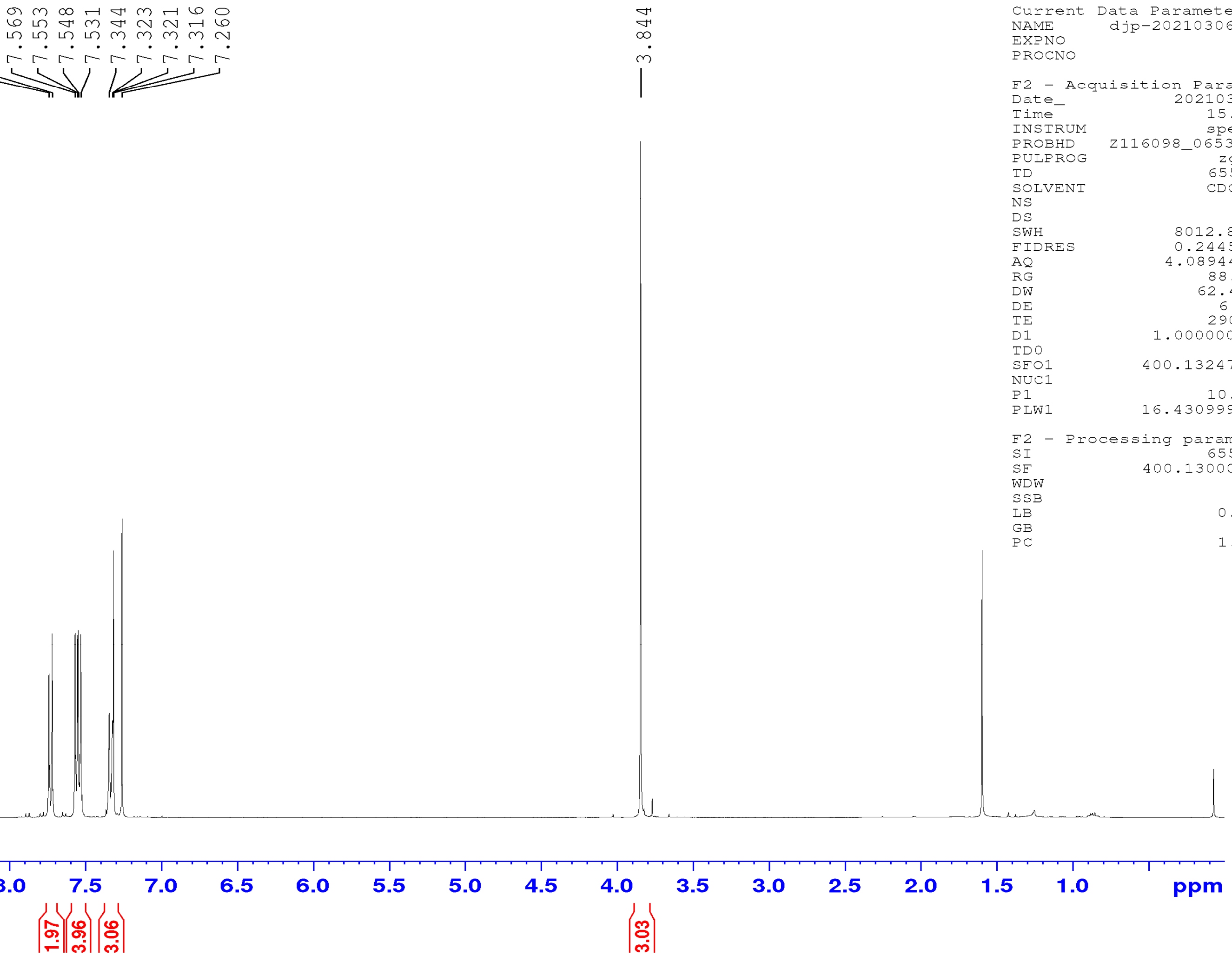


```
Current Data Parameters
NAME       djp-20210306-3
EXPNO                   1
PROCNO                  1

F2 - Acquisition Parameters
Date_           20210306
Time               15.59 h
INSTRUM            spect
PROBHD    Z116098_0653 (
PULPROG             zg30
TD                 65536
SOLVENT            CDCl3
NS                     8
DS                     2
SWH             8012.820 Hz
FIDRES          0.244532 Hz
AQ             4.0894465 sec
RG                 88.22
DW                62.400 usec
DE                  6.50 usec
TE                 290.1 K
D1            1.00000000 sec
TD0                    1
SFO1         400.1324708 MHz
NUC1                  1H
P1                 10.00 usec
PLW1         16.43099976 W

F2 - Processing parameters
SI                 65536
SF           400.1300099 MHz
WDW                   EM
SSB                    0
LB                  0.30 Hz
GB                     0
PC                  1.00
```

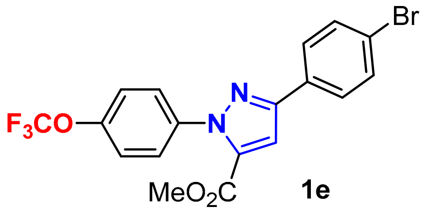

1e

^{13}C NMR (100 MHz, $CDCl_3$)

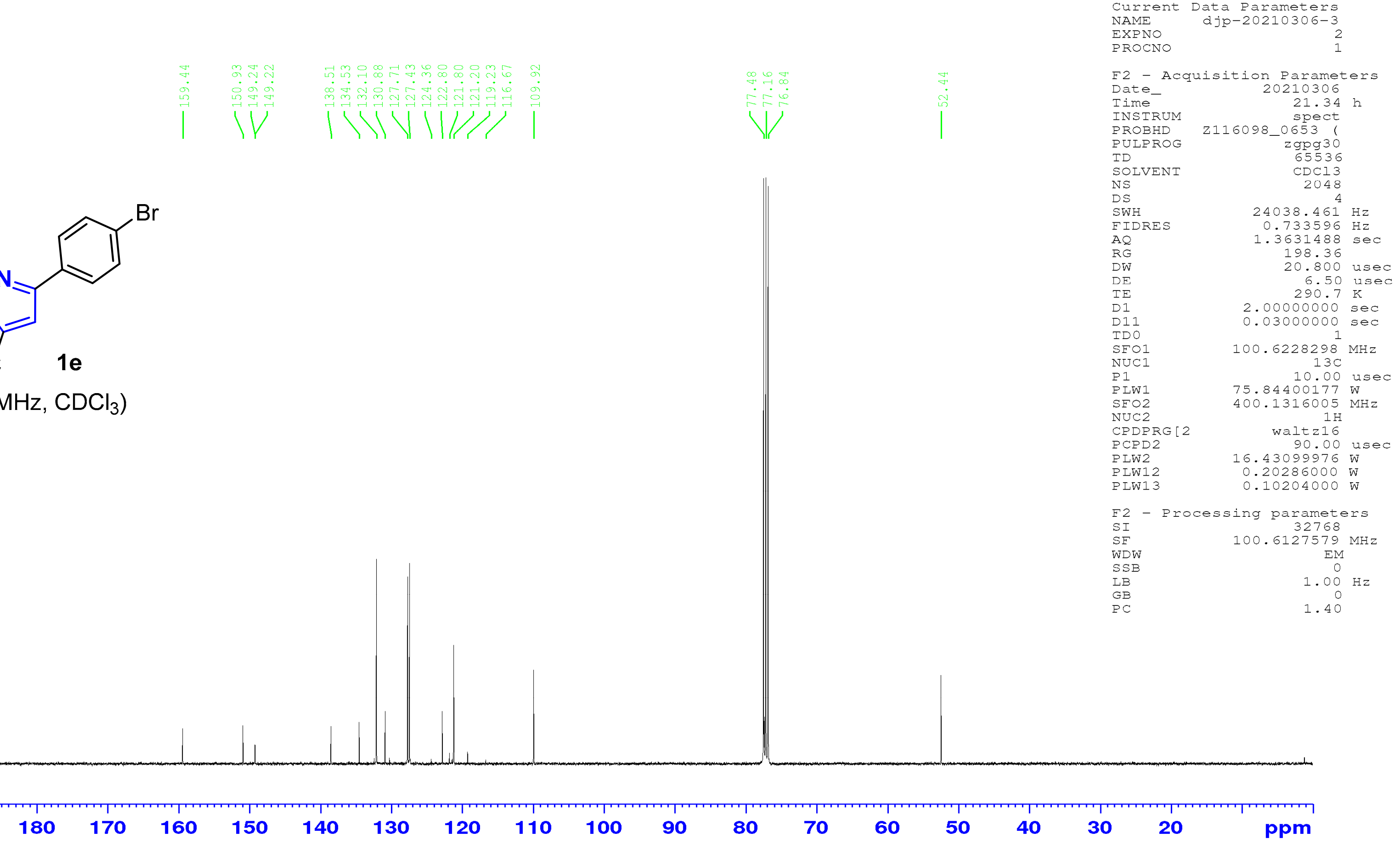

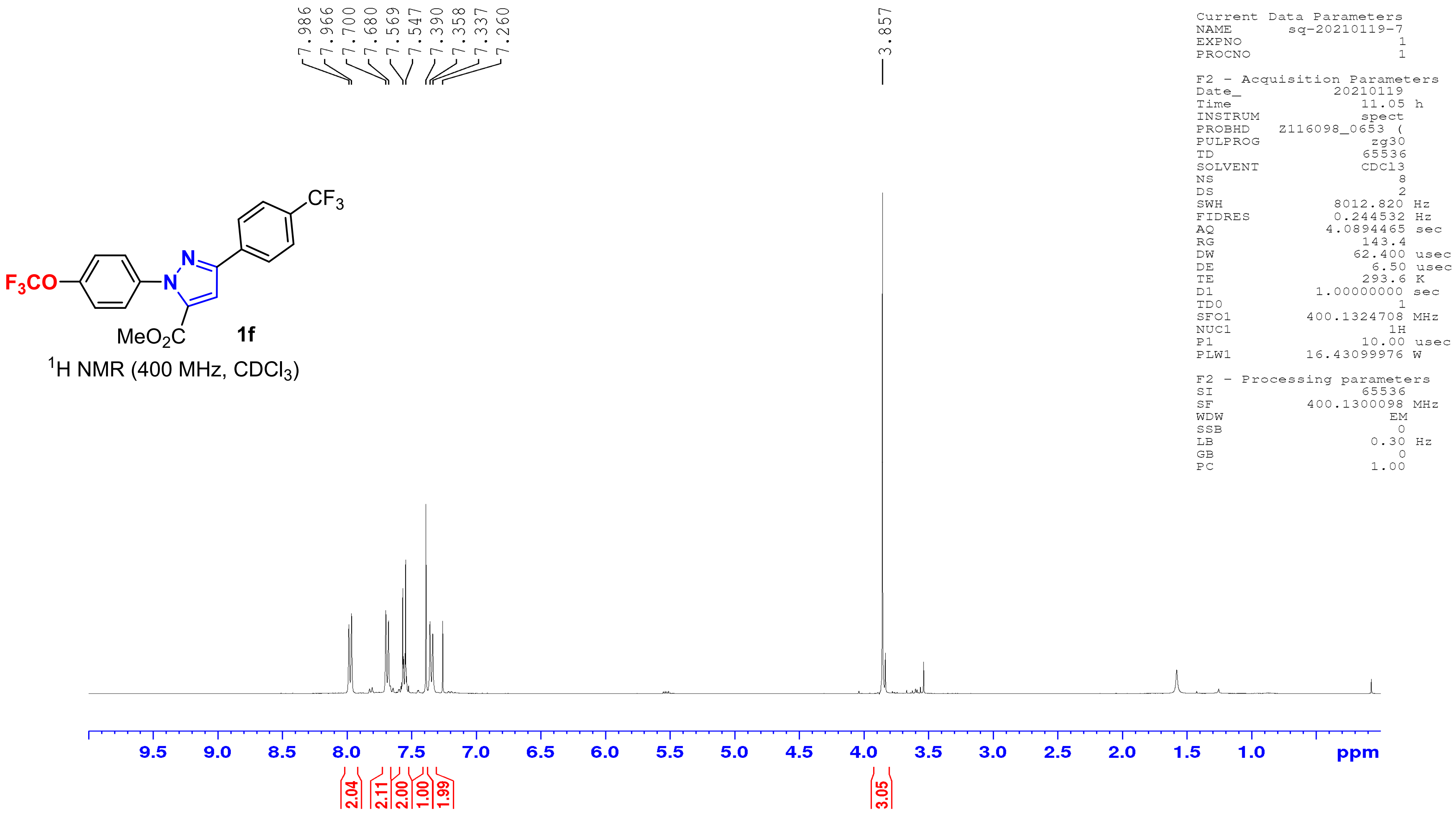
7.986
7.966
7.700
7.680
7.569
7.547
7.390
7.358
7.337
7.260
3.857
CF3
N
F3CO
N
MeO2C
1f
1H NMR (400 MHz, CDCl3)
Current Data Parameters
NAME sq-20210119-7
EXPNO 1
PROCNO 1
F2 - Acquisition Parameters
Date_ 20210119
Time 11.05 h
INSTRUM spect
PROBHD Z116098_0653 (
PULPROG zg30
TD 65536
SOLVENT CDCl3
NS 8
DS 2
SWH 8012.820 Hz
FIDRES 0.244532 Hz
AQ 4.0894465 sec
RG 143.4
DW 62.400 usec
DE 6.50 usec
TE 293.6 K
D1 1.00000000 sec
TD0 1
SFO1 400.1324708 MHz
NUC1 1H
P1 10.00 usec
PLW1 16.43099976 W
F2 - Processing parameters
SI 65536
SF 400.1300098 MHz
WDW EM
SSB 0
LB 0.30 Hz
GB 0
PC 1.00
9.5
9.0
8.5
8.0
7.5
7.0
6.5
6.0
5.5
5.0
4.5
4.0
3.5
3.0
2.5
2.0
1.5
1.0
ppm
2.04
2.11
2.00
1.00
1.99
3.05

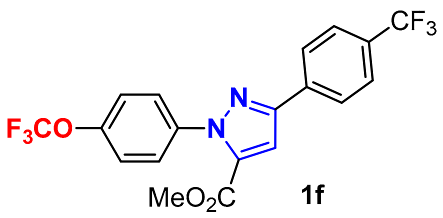

1f

^{13}C NMR (100 MHz, $CDCl_3$)

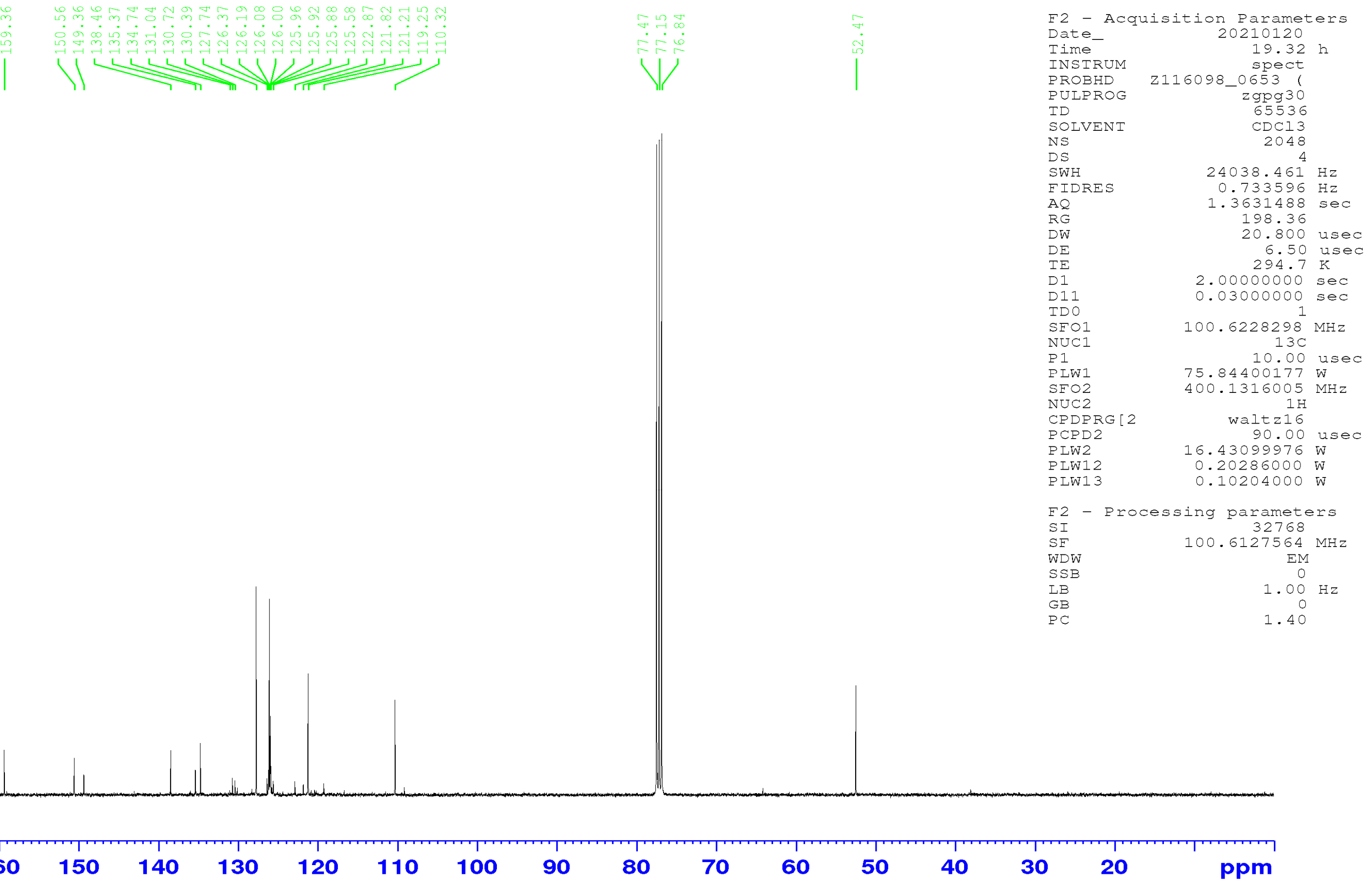

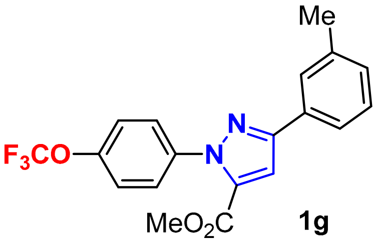

1g

^{1}H NMR (400 MHz, $CDCl_3$)

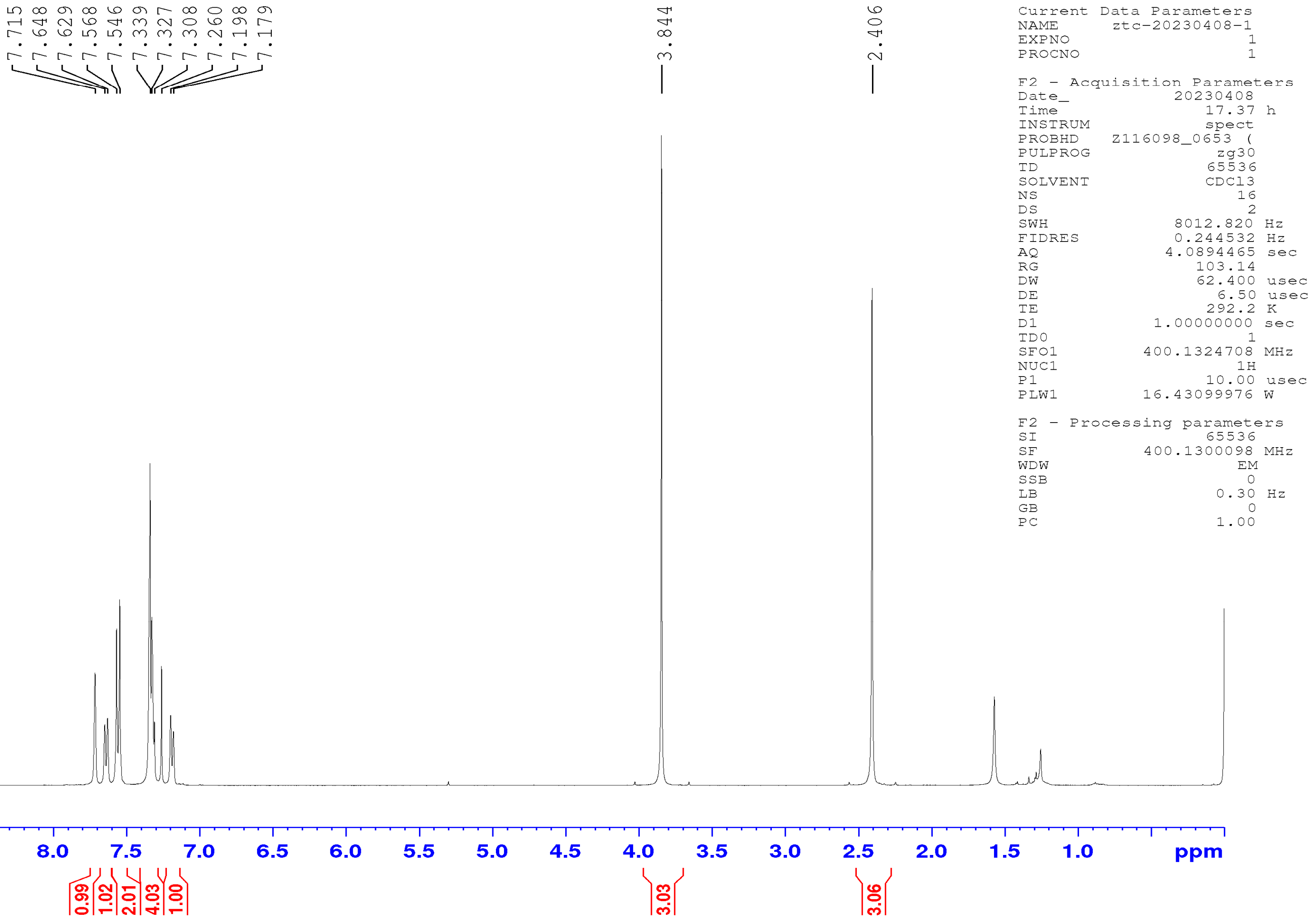

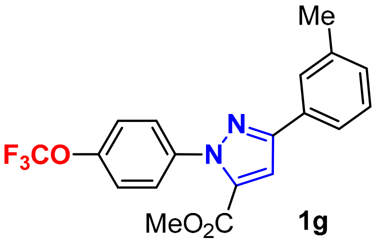

1g

^{13}C NMR (100 MHz, $CDCl_3$)

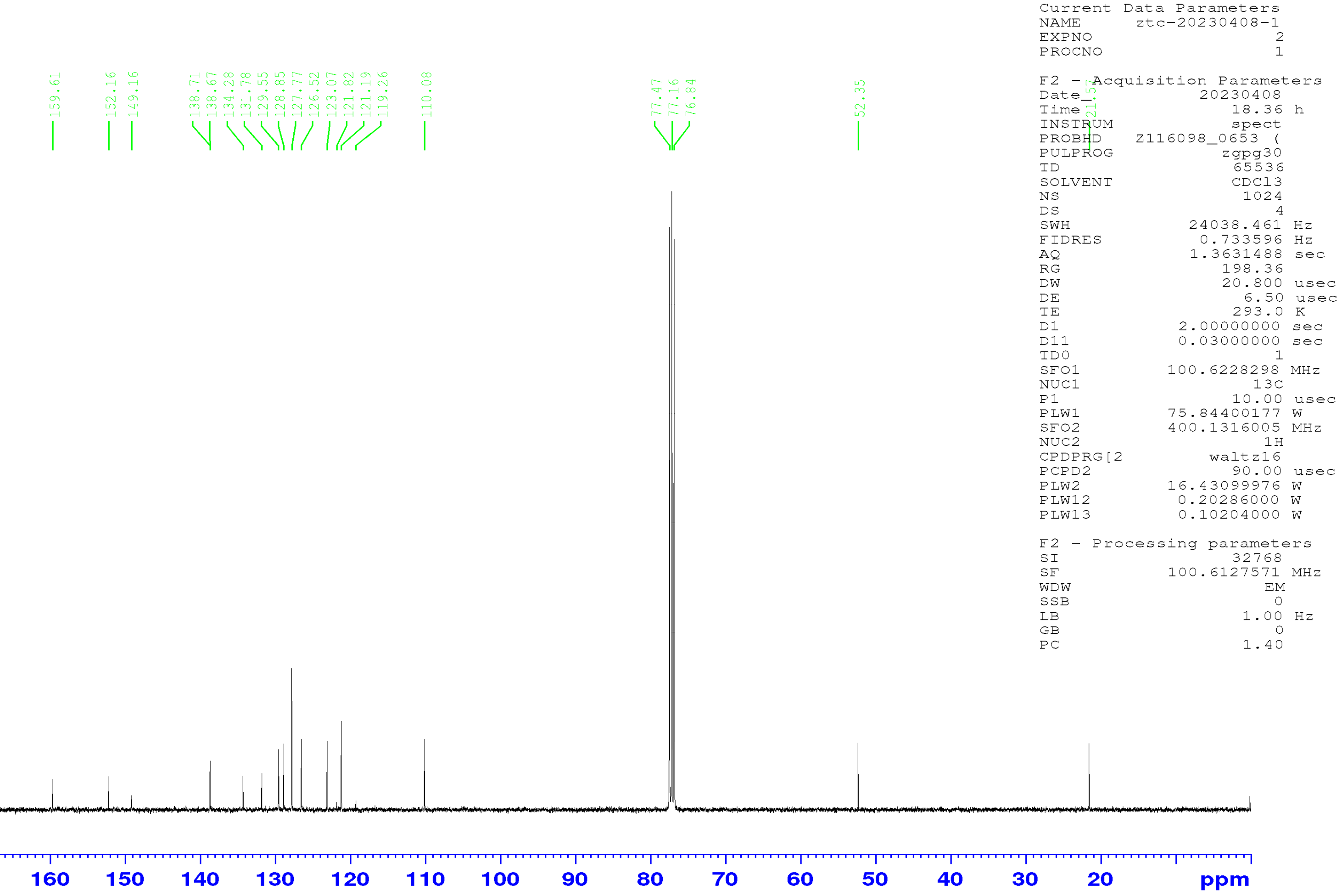

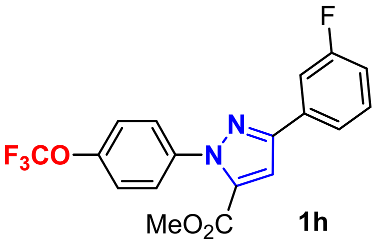

1h

^{1}H NMR (400 MHz, $CDCl_3$)

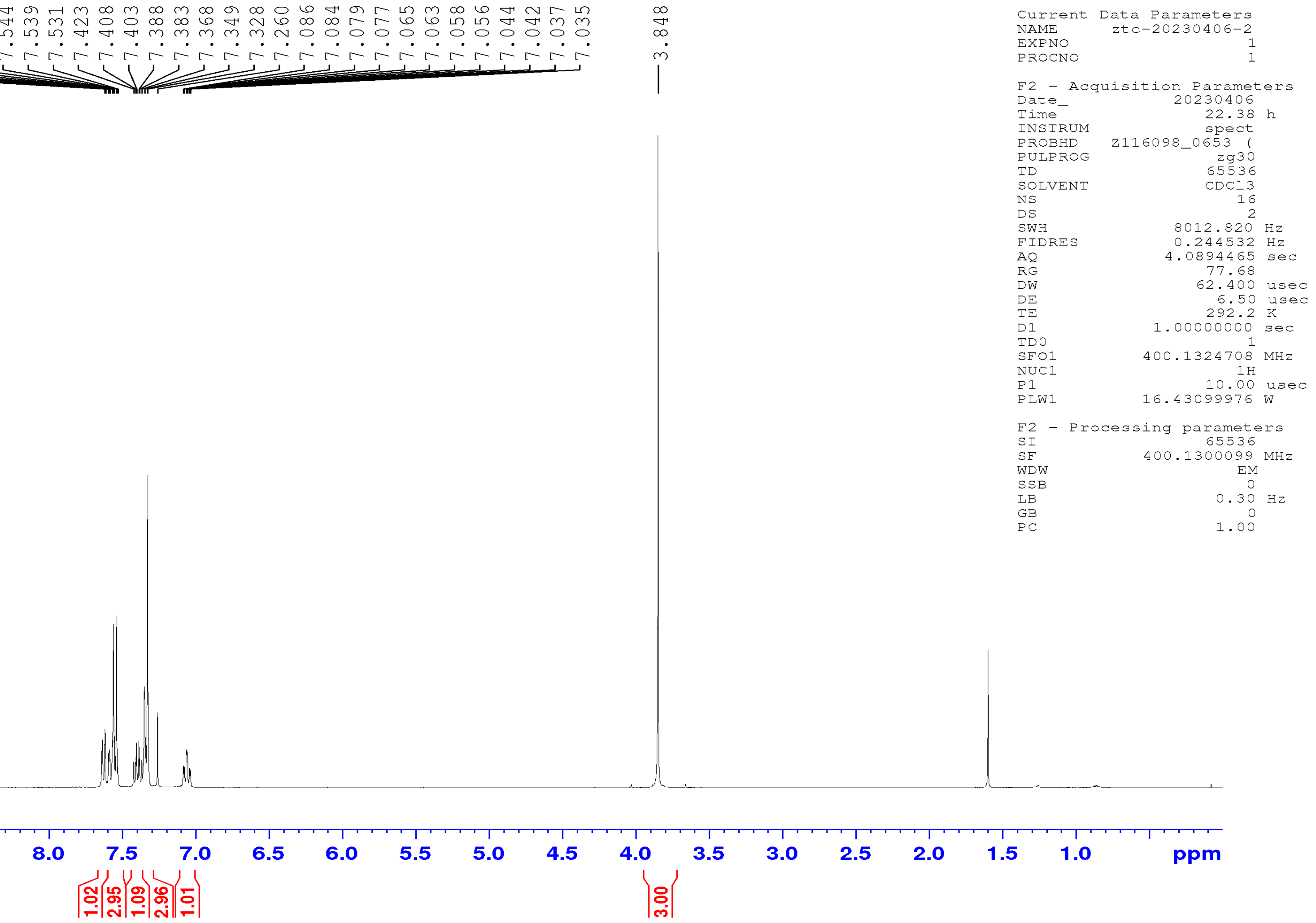

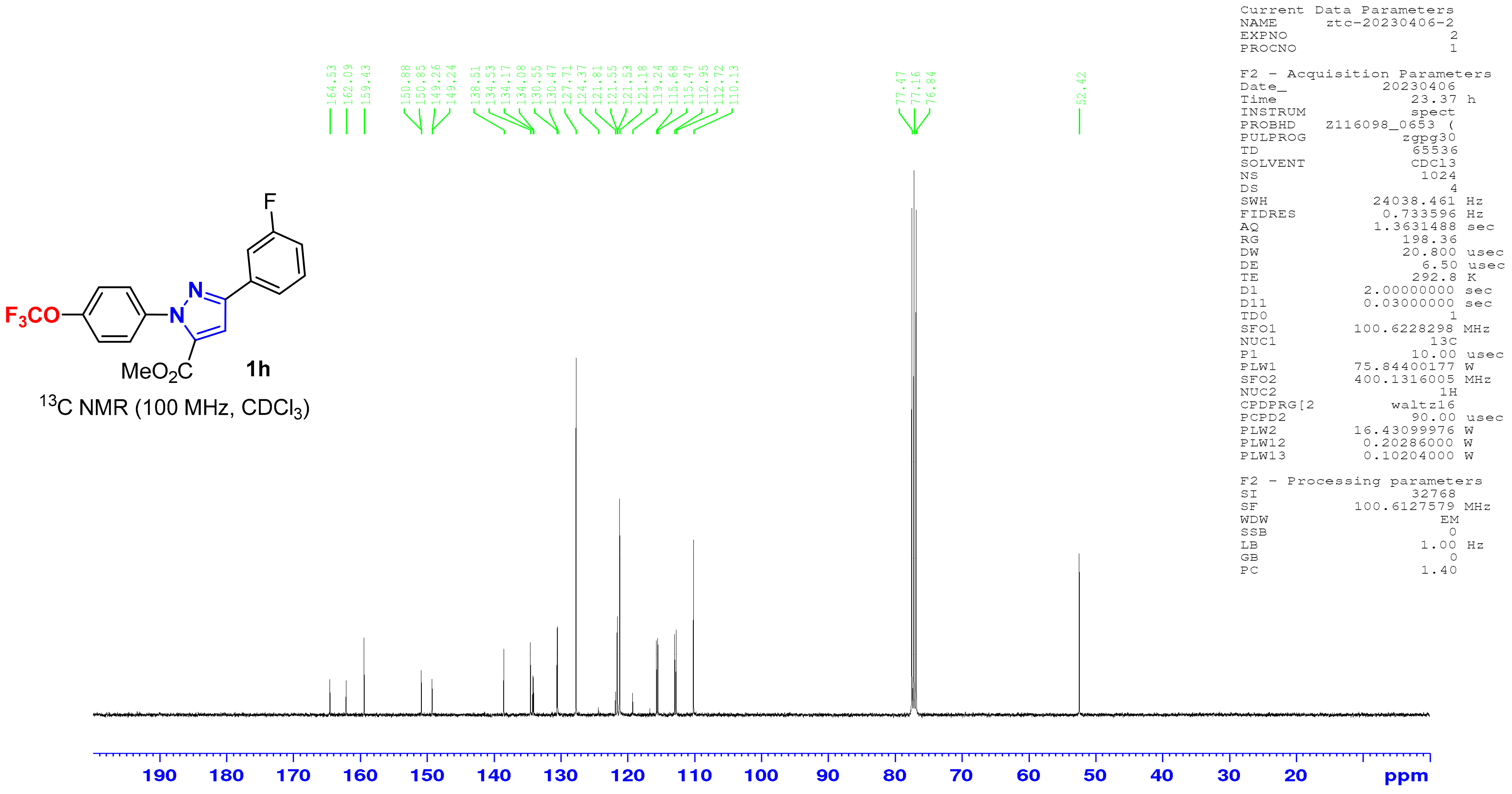
F
F3CO
N
N
MeO2C
1h
13C NMR (100 MHz, CDCl3)
164.53
162.09
159.43
150.88
150.85
149.26
149.24
138.51
134.53
134.17
134.08
130.55
130.47
127.71
124.37
121.81
121.55
121.53
121.18
119.24
115.68
115.47
112.95
112.72
110.13
77.47
77.16
76.84
52.42
Current Data Parameters
NAME ztc-20230406-2
EXPNO 2
PROCNO 1
F2 - Acquisition Parameters
Date_ 20230406
Time 23.37 h
INSTRUM spect
PROBHD Z116098_0653 (
PULPROG zgpg30
TD 65536
SOLVENT CDCl3
NS 1024
DS 4
SWH 24038.461 Hz
FIDRES 0.733596 Hz
AQ 1.3631488 sec
RG 198.36
DW 20.800 usec
DE 6.50 usec
TE 292.8 K
D1 2.00000000 sec
D11 0.03000000 sec
TD0 1
SFO1 100.6228298 MHz
NUC1 13C
P1 10.00 usec
PLW1 75.84400177 W
SFO2 400.1316005 MHz
NUC2 1H
CPDPRG[2 waltz16
PCPD2 90.00 usec
PLW2 16.43099976 W
PLW12 0.20286000 W
PLW13 0.10204000 W
F2 - Processing parameters
SI 32768
SF 100.6127579 MHz
WDW EM
SSB 0
LB 1.00 Hz
GB 0
PC 1.40
190
180
170
160
150
140
130
120
110
100
90
80
70
60
50
40
30
20
ppm

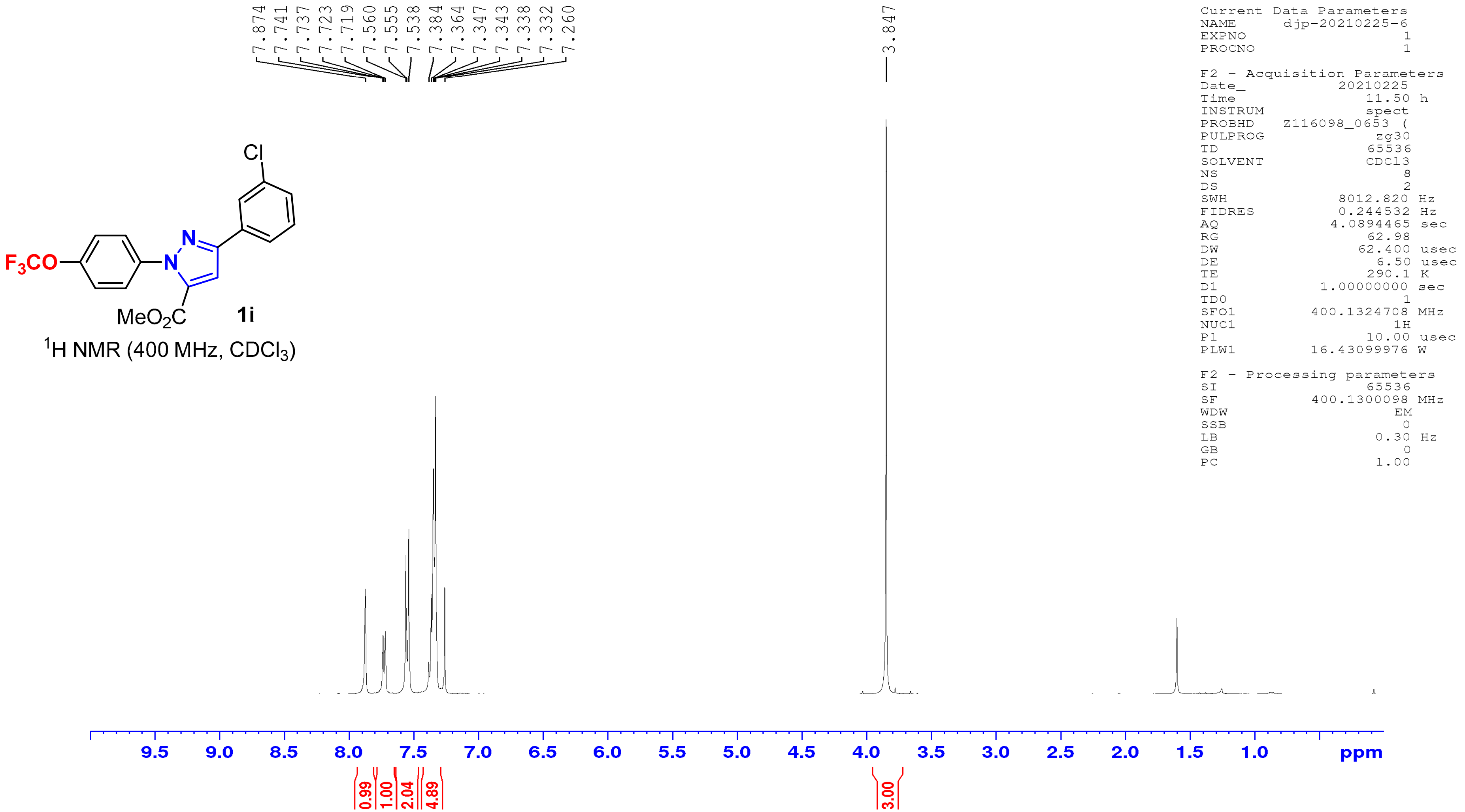

7.874
7.741
7.737
7.723
7.719
7.560
7.555
7.538
7.384
7.364
7.347
7.343
7.338
7.332
7.260
3.847
Cl
N
N
F3CO
MeO2C
1i
1H NMR (400 MHz, CDCl3)
9.5
9.0
8.5
8.0
7.5
7.0
6.5
6.0
5.5
5.0
4.5
4.0
3.5
3.0
2.5
2.0
1.5
1.0
ppm
0.99
1.00
2.04
4.89
3.00
Current Data Parameters
NAME djp-20210225-6
EXPNO 1
PROCNO 1
F2 - Acquisition Parameters
Date_ 20210225
Time 11.50 h
INSTRUM spect
PROBHD Z116098_0653 (
PULPROG zg30
TD 65536
SOLVENT CDCl3
NS 8
DS 2
SWH 8012.820 Hz
FIDRES 0.244532 Hz
AQ 4.0894465 sec
RG 62.98
DW 62.400 usec
DE 6.50 usec
TE 290.1 K
D1 1.00000000 sec
TD0 1
SFO1 400.1324708 MHz
NUC1 1H
P1 10.00 usec
PLW1 16.43099976 W
F2 - Processing parameters
SI 65536
SF 400.1300098 MHz
WDW EM
SSB 0
LB 0.30 Hz
GB 0
PC 1.00

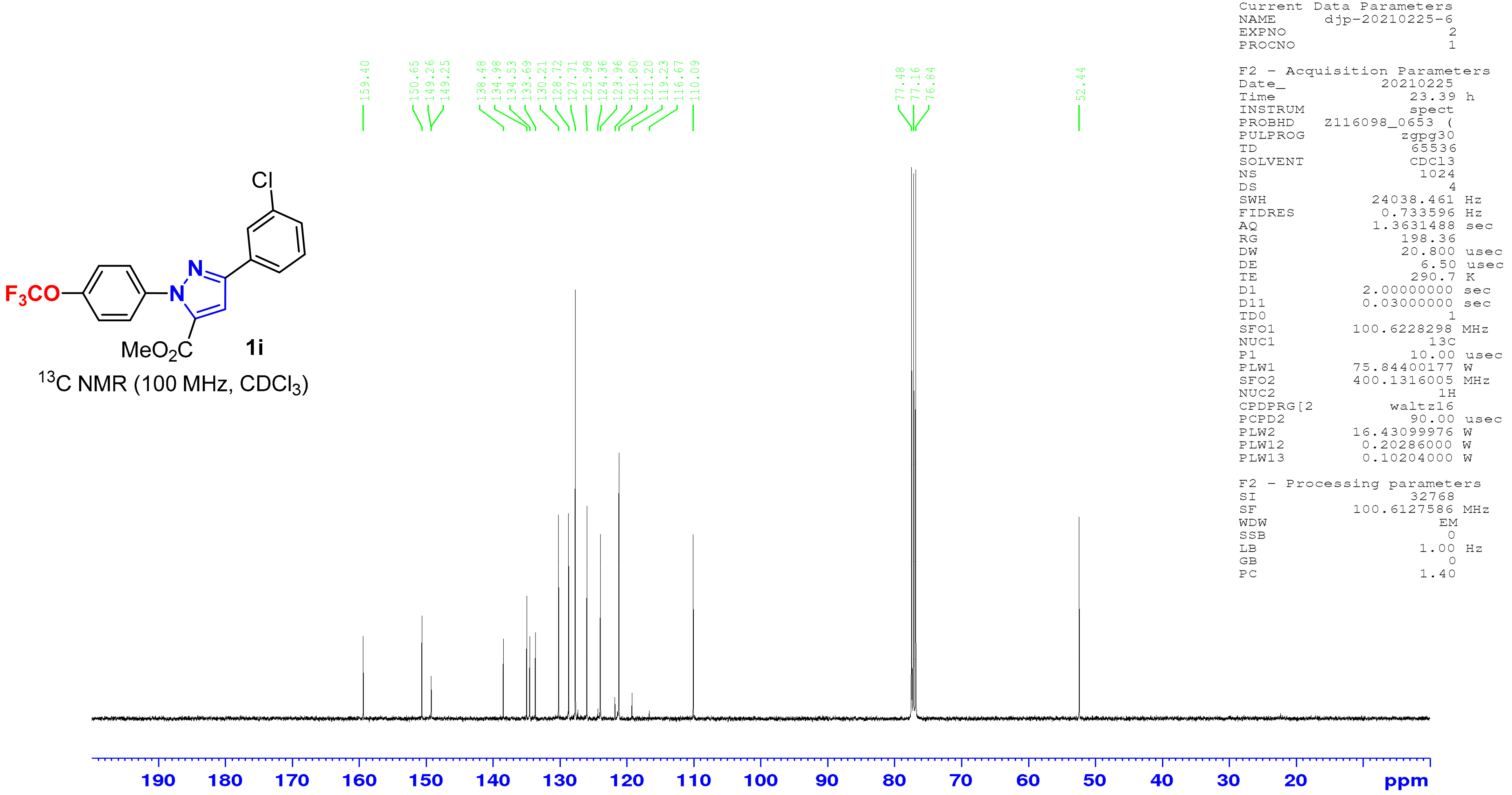
159.40
150.65
149.26
149.25
138.48
134.98
134.53
133.69
130.21
128.72
127.71
125.98
124.36
123.96
121.80
121.20
119.23
116.67
110.09
77.48
77.16
76.84
52.44
Cl
N
N
F_3CO
MeO_2C
1i
^{13}C NMR (100 MHz, $CDCl_3$)
Current Data Parameters
NAME djp-20210225-6
EXPNO 2
PROCNO 1
F2 - Acquisition Parameters
Date_ 20210225
Time 23.39 h
INSTRUM spect
PROBHD Z116098_0653 (
PULPROG zgpg30
TD 65536
SOLVENT CDCl3
NS 1024
DS 4
SWH 24038.461 Hz
FIDRES 0.733596 Hz
AQ 1.3631488 sec
RG 198.36
DW 20.800 usec
DE 6.50 usec
TE 290.7 K
D1 2.00000000 sec
D11 0.03000000 sec
TD0 1
SFO1 100.6228298 MHz
NUC1 13C
P1 10.00 usec
PLW1 75.84400177 W
SFO2 400.1316005 MHz
NUC2 1H
CPDPRG[2 waltz16
PCPD2 90.00 usec
PLW2 16.43099976 W
PLW12 0.20286000 W
PLW13 0.10204000 W
F2 - Processing parameters
SI 32768
SF 100.6127586 MHz
WDW EM
SSB 0
LB 1.00 Hz
GB 0
PC 1.40
190 180 170 160 150 140 130 120 110 100 90 80 70 60 50 40 30 20 ppm

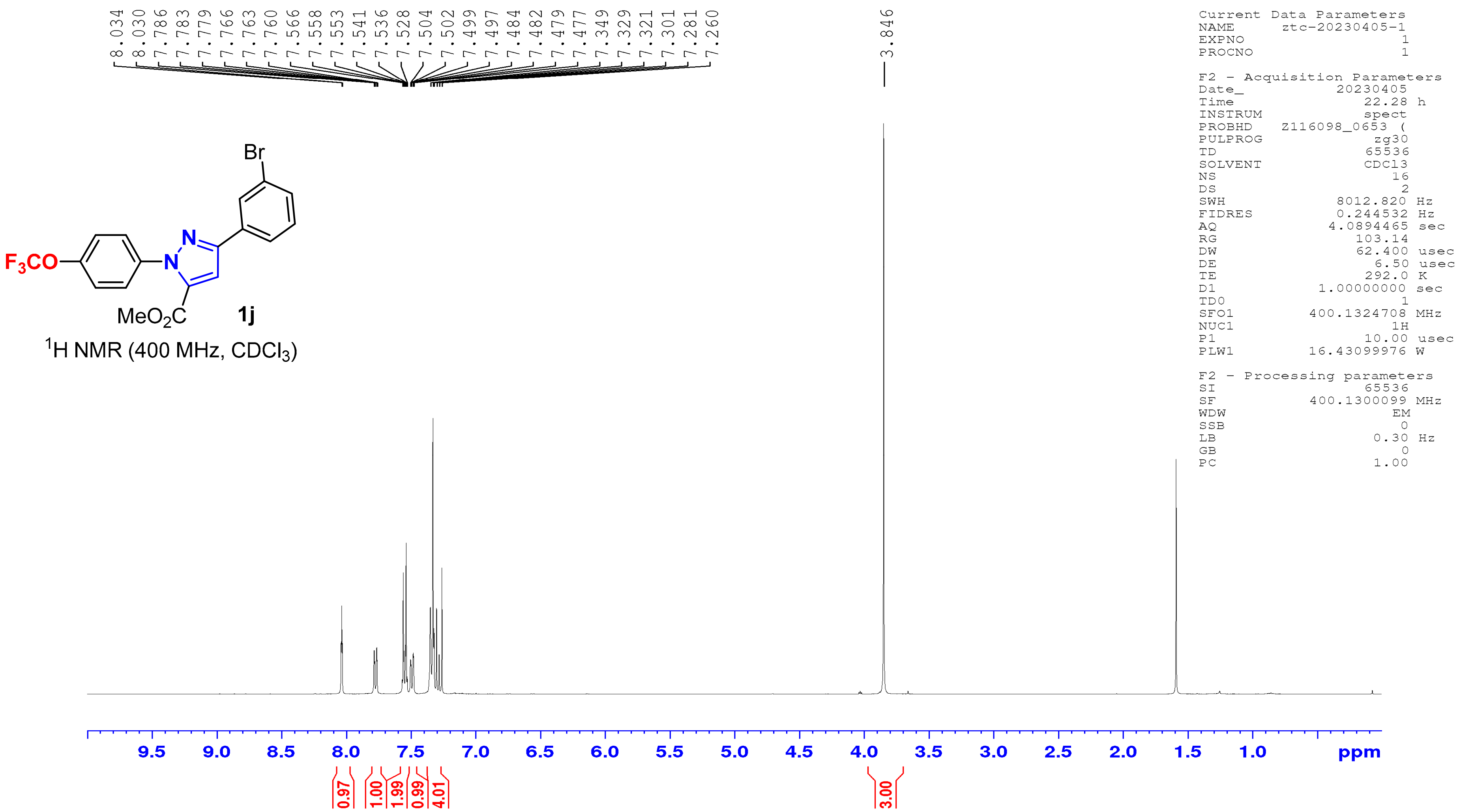
8.034 8.030 7.786 7.783 7.779 7.766 7.763 7.760 7.566 7.558 7.553 7.541 7.536 7.528 7.504 7.502 7.499 7.497 7.484 7.482 7.479 7.477 7.349 7.329 7.321 7.301 7.281 7.260
3.846
Br
N
N
F_3CO
MeO_2C
1j
^{1}H NMR (400 MHz, $CDCl_3$)
Current Data Parameters
NAME ztc-20230405-1
EXPNO 1
PROCNO 1
F2 - Acquisition Parameters
Date_ 20230405
Time 22.28 h
INSTRUM spect
PROBHD Z116098_0653 (
PULPROG zg30
TD 65536
SOLVENT CDCl3
NS 16
DS 2
SWH 8012.820 Hz
FIDRES 0.244532 Hz
AQ 4.0894465 sec
RG 103.14
DW 62.400 usec
DE 6.50 usec
TE 292.0 K
D1 1.00000000 sec
TD0 1
SFO1 400.1324708 MHz
NUC1 1H
P1 10.00 usec
PLW1 16.43099976 W
F2 - Processing parameters
SI 65536
SF 400.1300099 MHz
WDW EM
SSB 0
LB 0.30 Hz
GB 0
PC 1.00
9.5 9.0 8.5 8.0 7.5 7.0 6.5 6.0 5.5 5.0 4.5 4.0 3.5 3.0 2.5 2.0 1.5 1.0 ppm
0.97 1.00 1.99 0.99 4.01 3.00

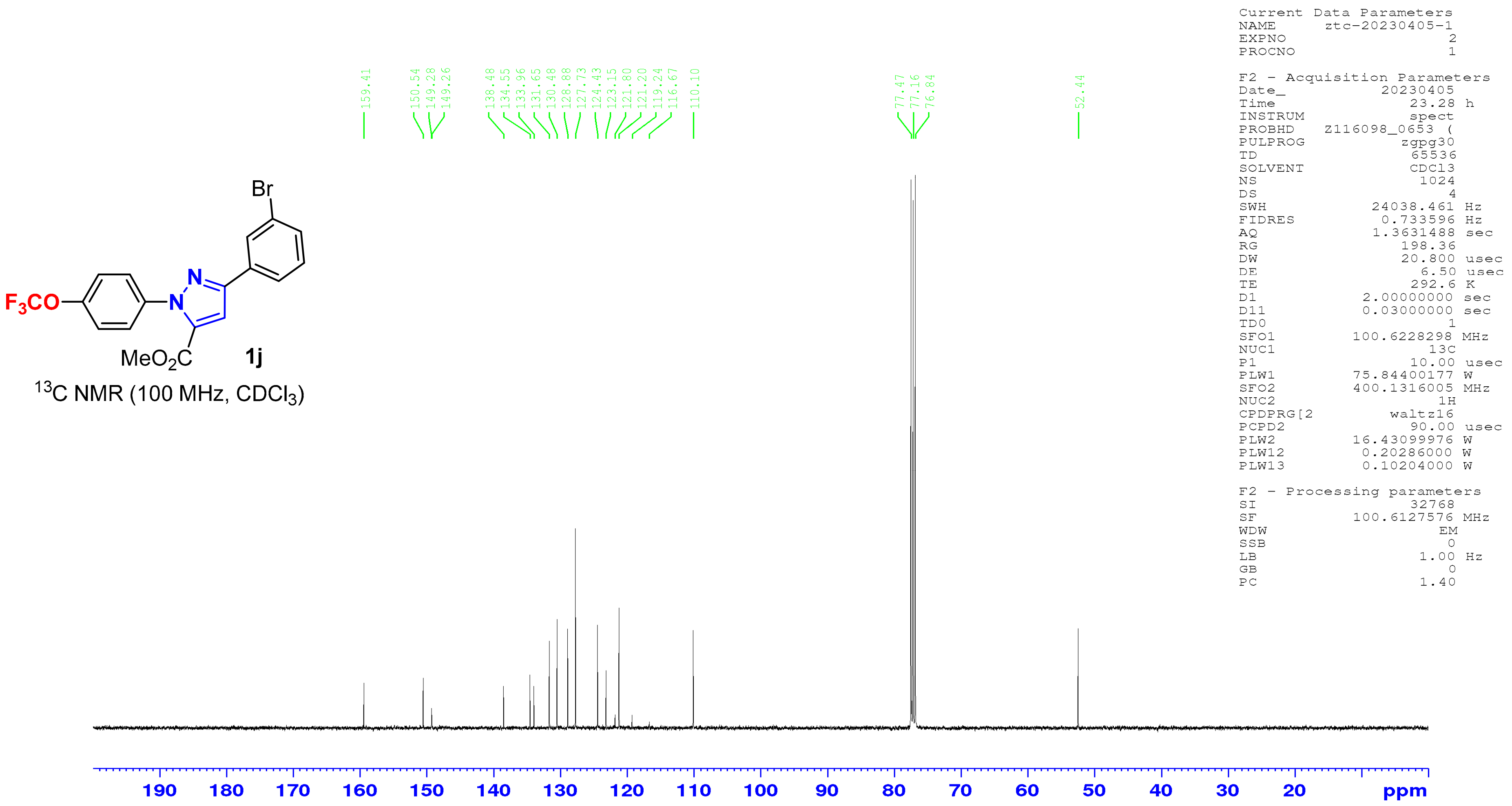
Br
F_3CO
N
N
MeO_2C
1j
^{13}C NMR (100 MHz, $CDCl_3$)
159.41
150.54
149.28
149.26
138.48
134.55
133.96
131.65
130.48
128.88
127.73
124.43
123.15
121.80
121.20
119.24
116.67
110.10
77.47
77.16
76.84
52.44
Current Data Parameters
NAME ztc-20230405-1
EXPNO 2
PROCNO 1
F2 - Acquisition Parameters
Date_ 20230405
Time 23.28 h
INSTRUM spect
PROBHD Z116098_0653 (
PULPROG zgpg30
TD 65536
SOLVENT CDCl3
NS 1024
DS 4
SWH 24038.461 Hz
FIDRES 0.733596 Hz
AQ 1.3631488 sec
RG 198.36
DW 20.800 usec
DE 6.50 usec
TE 292.6 K
D1 2.00000000 sec
D11 0.03000000 sec
TD0 1
SFO1 100.6228298 MHz
NUC1 13C
P1 10.00 usec
PLW1 75.84400177 W
SFO2 400.1316005 MHz
NUC2 1H
CPDPRG[2 waltz16
PCPD2 90.00 usec
PLW2 16.43099976 W
PLW12 0.20286000 W
PLW13 0.10204000 W
F2 - Processing parameters
SI 32768
SF 100.6127576 MHz
WDW EM
SSB 0
LB 1.00 Hz
GB 0
PC 1.40
190
180
170
160
150
140
130
120
110
100
90
80
70
60
50
40
30
20
ppm

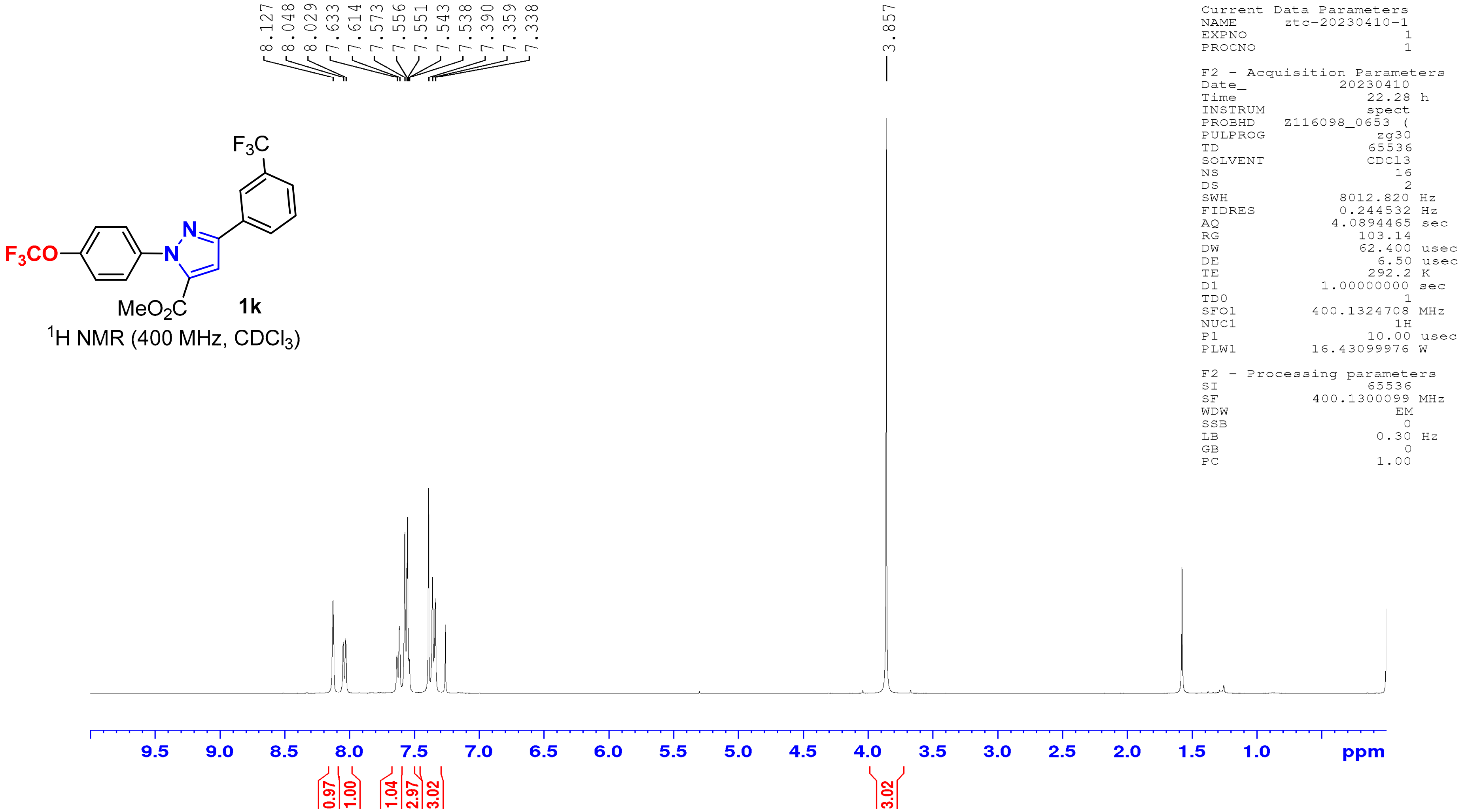
8.127
8.048
8.029
7.633
7.614
7.573
7.556
7.551
7.543
7.538
7.390
7.359
7.338
3.857
F3C
F3CO
N
N
MeO2C
1k
1H NMR (400 MHz, CDCl3)
Current Data Parameters
NAME ztc-20230410-1
EXPNO 1
PROCNO 1
F2 - Acquisition Parameters
Date_ 20230410
Time 22.28 h
INSTRUM spect
PROBHD Z116098_0653 (
PULPROG zg30
TD 65536
SOLVENT CDCl3
NS 16
DS 2
SWH 8012.820 Hz
FIDRES 0.244532 Hz
AQ 4.0894465 sec
RG 103.14
DW 62.400 usec
DE 6.50 usec
TE 292.2 K
D1 1.00000000 sec
TD0 1
SFO1 400.1324708 MHz
NUC1 1H
P1 10.00 usec
PLW1 16.43099976 W
F2 - Processing parameters
SI 65536
SF 400.1300099 MHz
WDW EM
SSB 0
LB 0.30 Hz
GB 0
PC 1.00
9.5
9.0
8.5
8.0
7.5
7.0
6.5
6.0
5.5
5.0
4.5
4.0
3.5
3.0
2.5
2.0
1.5
1.0
ppm
0.97
1.00
1.04
2.97
3.02
3.02

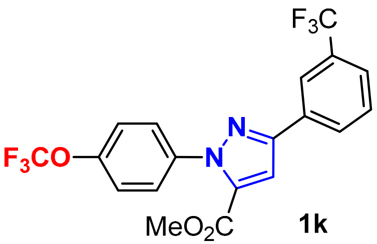

1k

^{13}C NMR (100 MHz, $CDCl_3$)

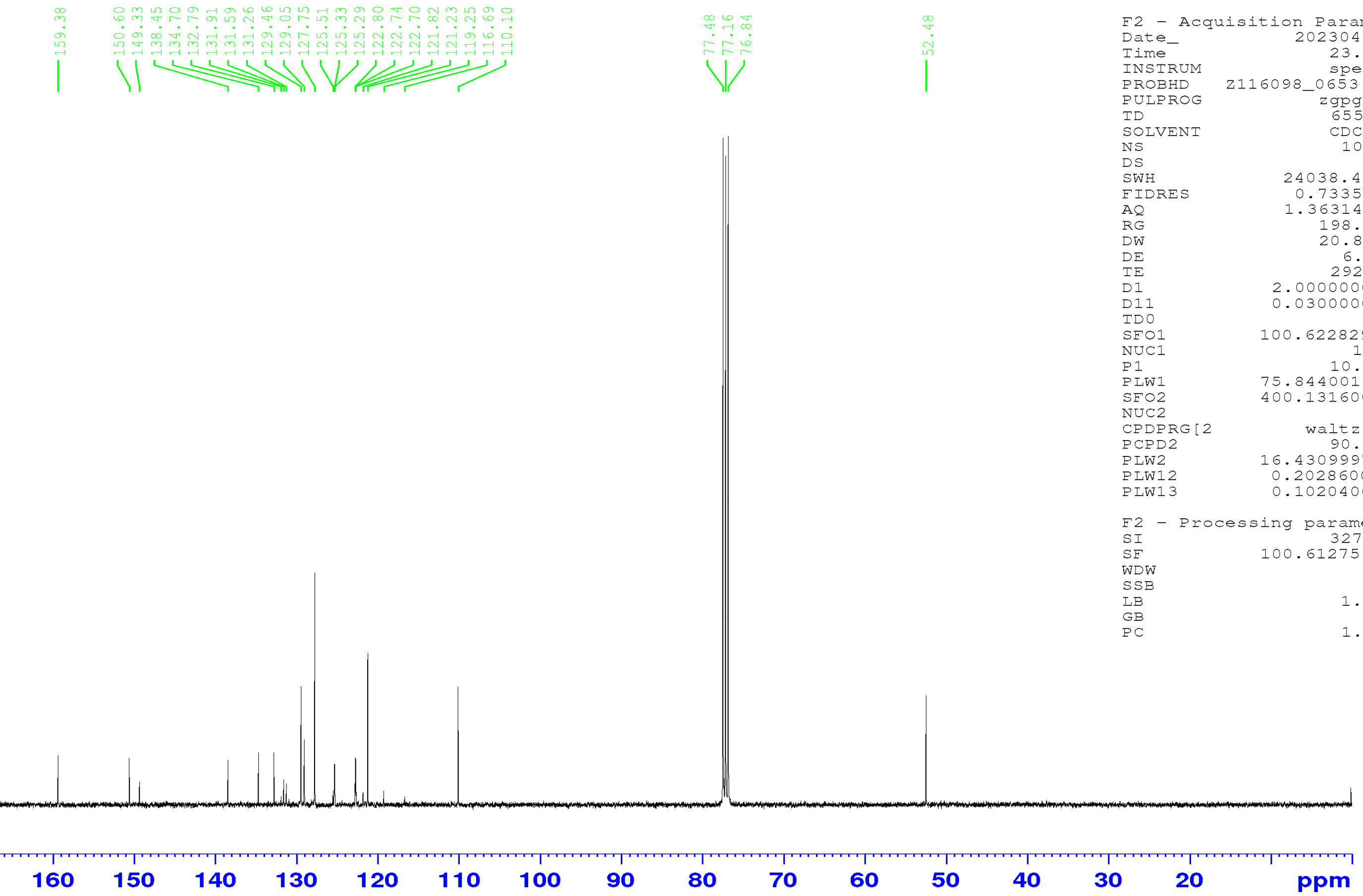


```
Current Data Parameters
NAME       ztc-20230410-1
EXPNO                   2
PROCNO                  1

F2 - Acquisition Parameters
Date_            20230410
Time                23.28 h
INSTRUM             spect
PROBHD    Z116098_0653 (
PULPROG           zgpg30
TD                 65536
SOLVENT            CDCl3
NS                  1024
DS                     4
SWH            24038.461 Hz
FIDRES          0.733596 Hz
AQ             1.3631488 sec
RG                198.36
DW                20.800 usec
DE                  6.50 usec
TE                 292.5 K
D1            2.00000000 sec
D11           0.03000000 sec
TD0                    1
SFO1         100.6228298 MHz
NUC1                 13C
P1                 10.00 usec
PLW1         75.84400177 W
SFO2         400.1316005 MHz
NUC2                  1H
CPDPRG[2          waltz16
PCPD2              90.00 usec
PLW2         16.43099976 W
PLW12         0.20286000 W
PLW13         0.10204000 W

F2 - Processing parameters
SI                 32768
SF           100.6127564 MHz
WDW                   EM
SSB                    0
LB                  1.00 Hz
GB                     0
PC                  1.40
```

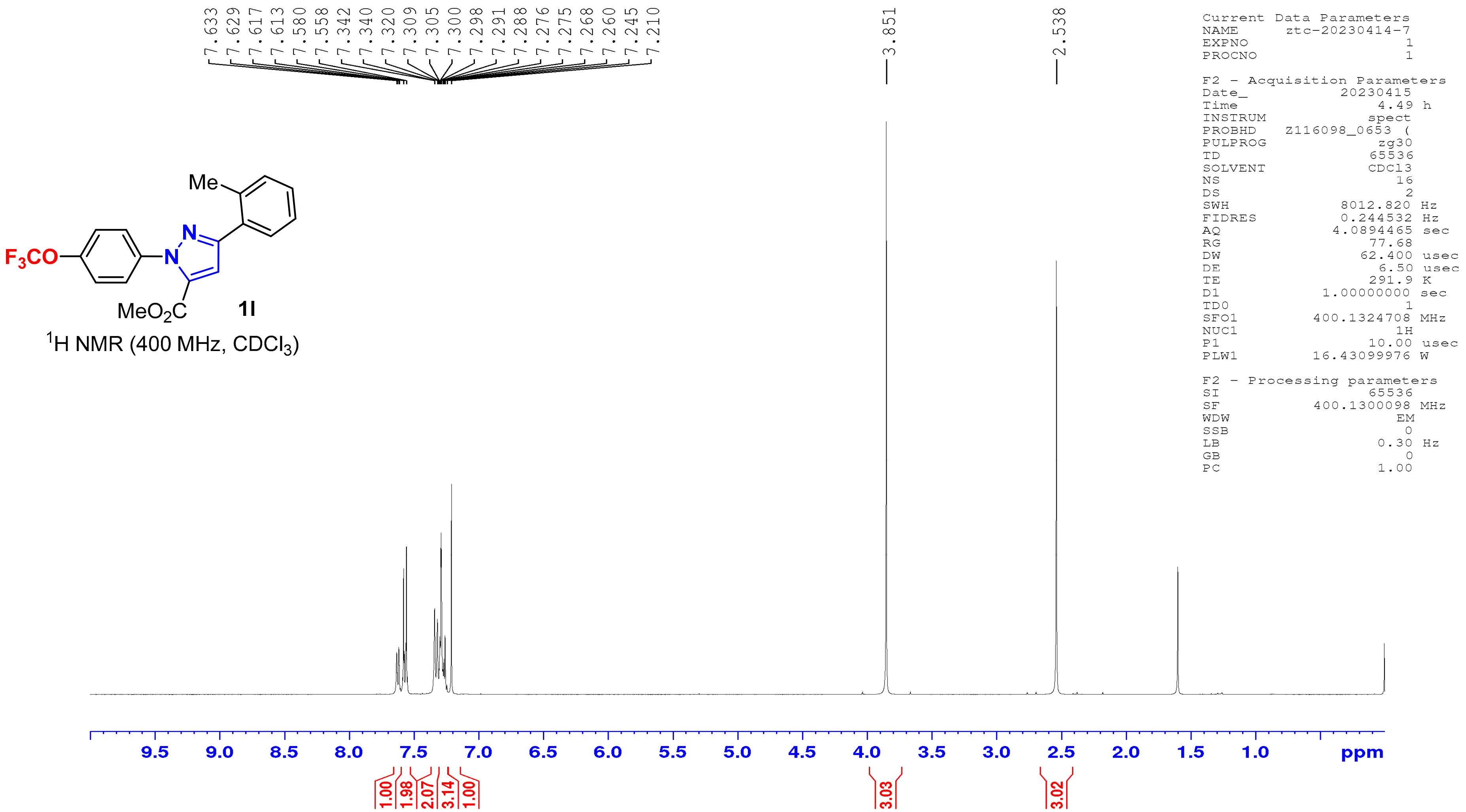
Me
F3CO
N
N
MeO2C
1l
1H NMR (400 MHz, CDCl3)
7.633
7.629
7.617
7.613
7.580
7.558
7.342
7.340
7.320
7.309
7.305
7.300
7.298
7.291
7.288
7.276
7.275
7.268
7.260
7.245
7.210
3.851
2.538
9.5
9.0
8.5
8.0
7.5
7.0
6.5
6.0
5.5
5.0
4.5
4.0
3.5
3.0
2.5
2.0
1.5
1.0
ppm
1.00
1.98
2.07
3.14
1.00
3.03
3.02
Current Data Parameters
NAME ztc-20230414-7
EXPNO 1
PROCNO 1
F2 - Acquisition Parameters
Date_ 20230415
Time 4.49 h
INSTRUM spect
PROBHD Z116098_0653 (
PULPROG zg30
TD 65536
SOLVENT CDCl3
NS 16
DS 2
SWH 8012.820 Hz
FIDRES 0.244532 Hz
AQ 4.0894465 sec
RG 77.68
DW 62.400 usec
DE 6.50 usec
TE 291.9 K
D1 1.00000000 sec
TD0 1
SFO1 400.1324708 MHz
NUC1 1H
P1 10.00 usec
PLW1 16.43099976 W
F2 - Processing parameters
SI 65536
SF 400.1300098 MHz
WDW EM
SSB 0
LB 0.30 Hz
GB 0
PC 1.00

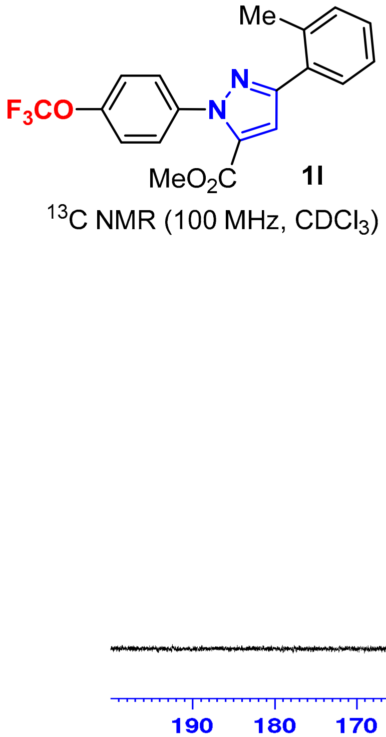

1l

^{13}C NMR (100 MHz, $CDCl_3$)

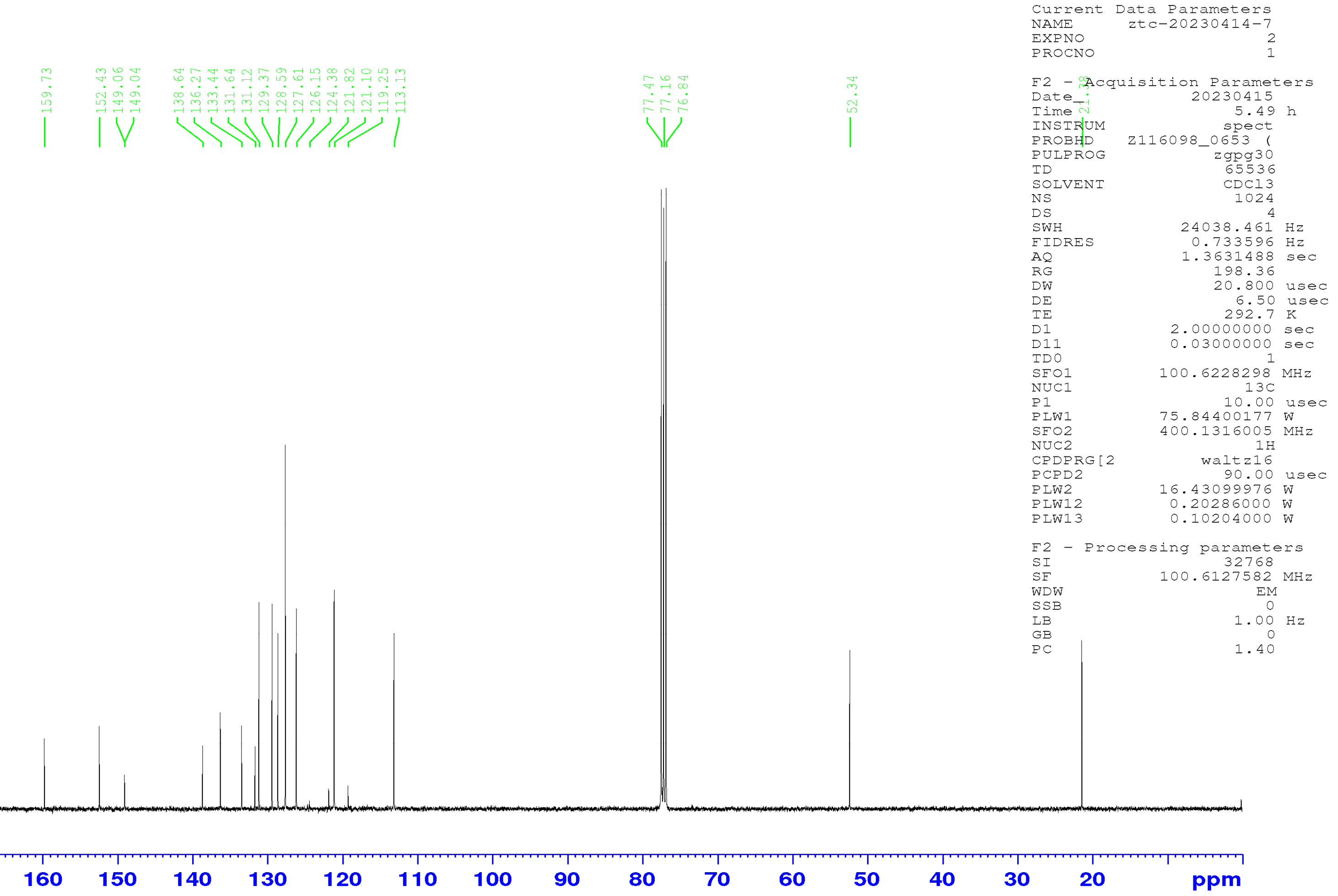

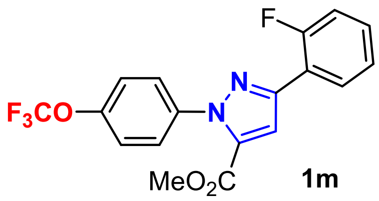

1m

^{1}H NMR (400 MHz, $CDCl_3$)

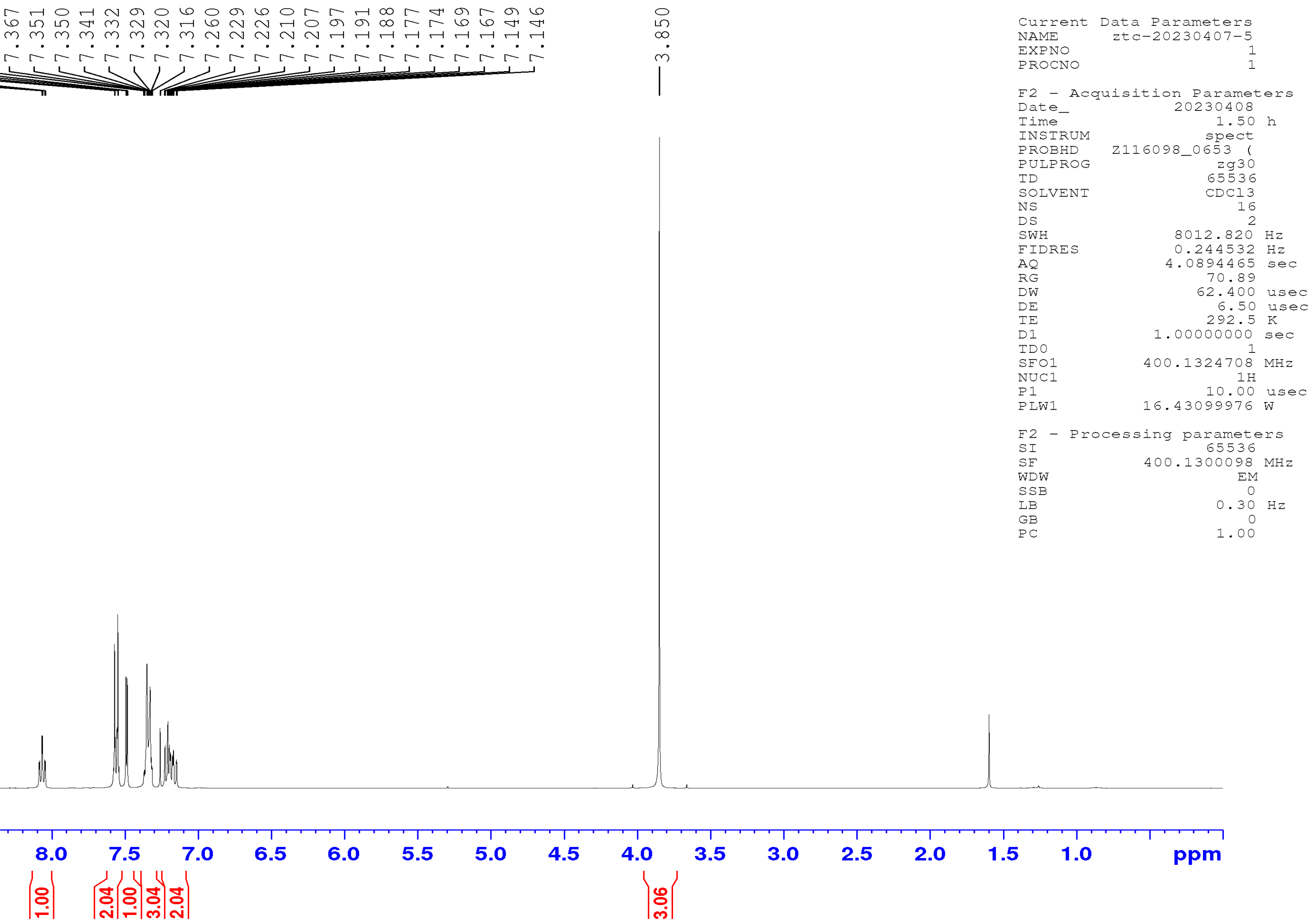

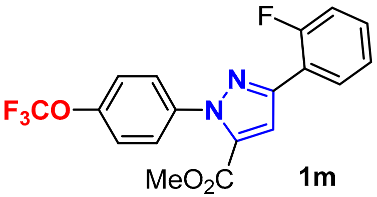

1m

^{13}C NMR (100 MHz, $CDCl_3$)

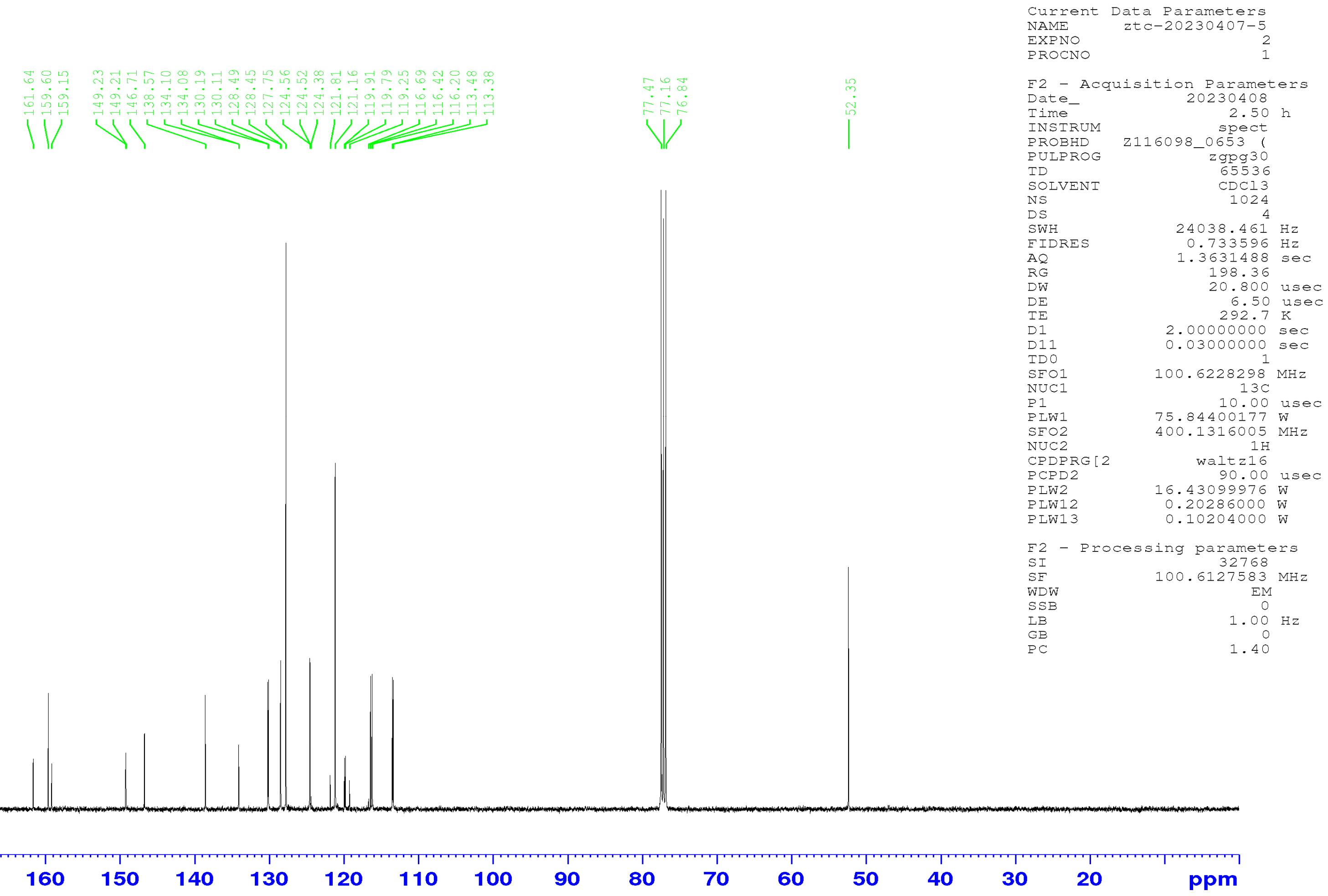

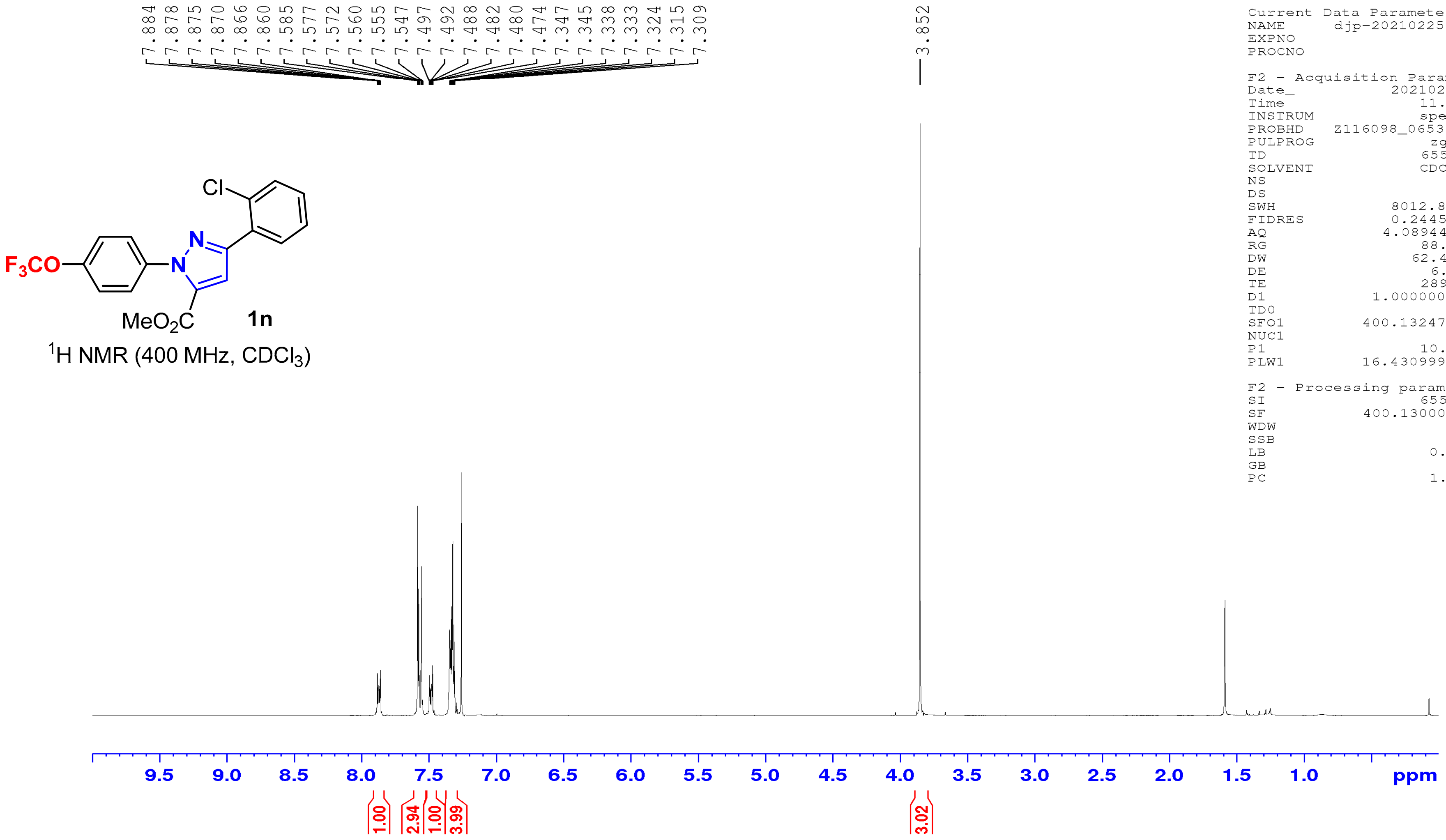

^{1}H NMR (400 MHz, $CDCl_3$)

```
Current Data Parameters
NAME       djp-20210225-4
EXPNO                   1
PROCNO                  1

F2 - Acquisition Parameters
Date_            20210225
Time                11.43 h
INSTRUM             spect
PROBHD     Z116098_0653 (
PULPROG              zg30
TD                  65536
SOLVENT             CDCl3
NS                      8
DS                      2
SWH              8012.820 Hz
FIDRES           0.244532 Hz
AQ              4.0894465 sec
RG                  88.22
DW                 62.400 usec
DE                   6.50 usec
TE                  289.9 K
D1             1.00000000 sec
TD0                     1
SFO1          400.1324708 MHz
NUC1                   1H
P1                  10.00 usec
PLW1          16.43099976 W

F2 - Processing parameters
SI                  65536
SF            400.1300099 MHz
WDW                    EM
SSB                     0
LB                   0.30 Hz
GB                      0
PC                   1.00
```

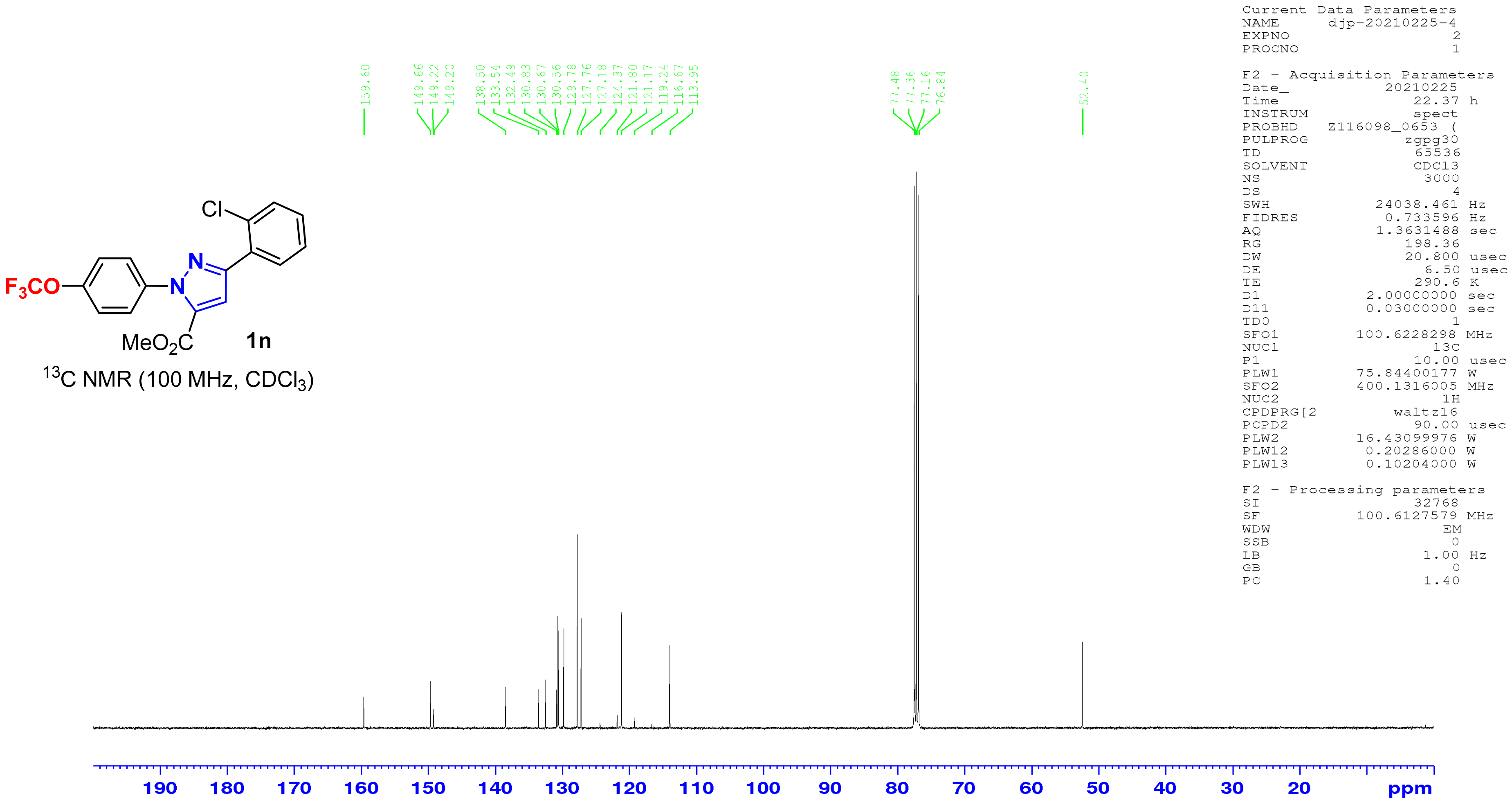
Cl
N
N
F3CO
MeO2C
1n
13C NMR (100 MHz, CDCl3)
159.60
149.66
149.22
149.20
138.50
133.54
132.49
130.83
130.67
130.56
129.78
127.76
127.18
124.37
121.80
121.17
119.24
116.67
113.95
77.48
77.36
77.16
76.84
52.40
190 180 170 160 150 140 130 120 110 100 90 80 70 60 50 40 30 20 ppm
Current Data Parameters
NAME djp-20210225-4
EXPNO 2
PROCNO 1
F2 - Acquisition Parameters
Date_ 20210225
Time 22.37 h
INSTRUM spect
PROBHD Z116098_0653 (
PULPROG zgpg30
TD 65536
SOLVENT CDCl3
NS 3000
DS 4
SWH 24038.461 Hz
FIDRES 0.733596 Hz
AQ 1.3631488 sec
RG 198.36
DW 20.800 usec
DE 6.50 usec
TE 290.6 K
D1 2.00000000 sec
D11 0.03000000 sec
TD0 1
SFO1 100.6228298 MHz
NUC1 13C
P1 10.00 usec
PLW1 75.84400177 W
SFO2 400.1316005 MHz
NUC2 1H
CPDPRG[2 waltz16
PCPD2 90.00 usec
PLW2 16.43099976 W
PLW12 0.20286000 W
PLW13 0.10204000 W
F2 - Processing parameters
SI 32768
SF 100.6127579 MHz
WDW EM
SSB 0
LB 1.00 Hz
GB 0
PC 1.40

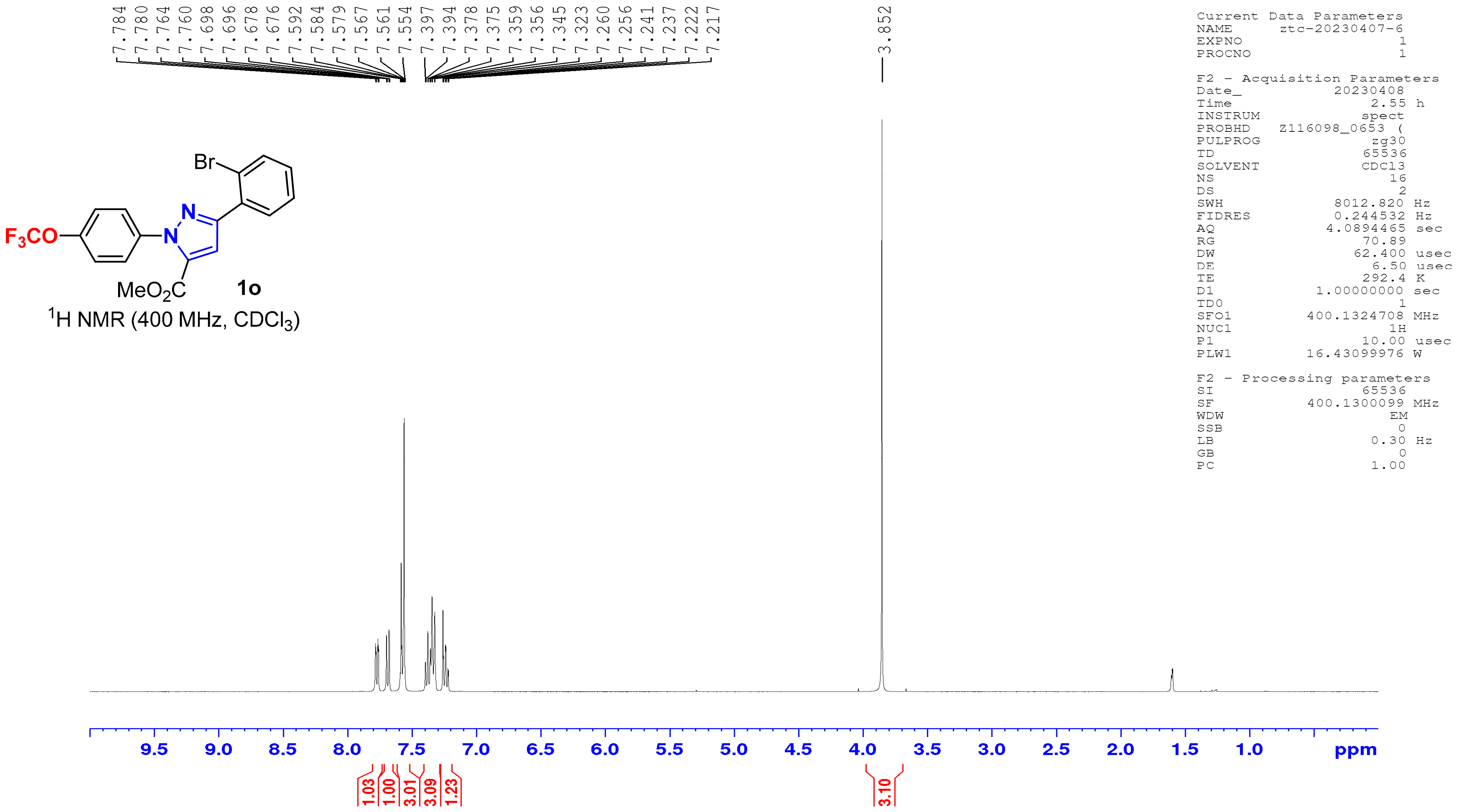

Br
N
N
F3CO
MeO2C
1o
1H NMR (400 MHz, CDCl3)
7.784
7.780
7.764
7.760
7.698
7.696
7.678
7.676
7.592
7.584
7.579
7.567
7.561
7.554
7.397
7.394
7.378
7.375
7.359
7.356
7.345
7.323
7.260
7.256
7.241
7.237
7.222
7.217
3.852
Current Data Parameters
NAME ztc-20230407-6
EXPNO 1
PROCNO 1
F2 - Acquisition Parameters
Date_ 20230408
Time 2.55 h
INSTRUM spect
PROBHD Z116098_0653 (
PULPROG zg30
TD 65536
SOLVENT CDCl3
NS 16
DS 2
SWH 8012.820 Hz
FIDRES 0.244532 Hz
AQ 4.0894465 sec
RG 70.89
DW 62.400 usec
DE 6.50 usec
TE 292.4 K
D1 1.00000000 sec
TD0 1
SFO1 400.1324708 MHz
NUC1 1H
P1 10.00 usec
PLW1 16.43099976 W
F2 - Processing parameters
SI 65536
SF 400.1300099 MHz
WDW EM
SSB 0
LB 0.30 Hz
GB 0
PC 1.00
9.5
9.0
8.5
8.0
7.5
7.0
6.5
6.0
5.5
5.0
4.5
4.0
3.5
3.0
2.5
2.0
1.5
1.0
ppm
1.03
1.00
3.01
3.09
1.23
3.10

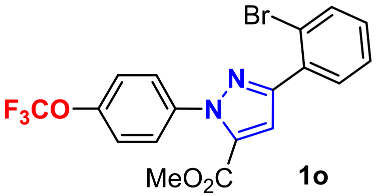

1o

^{13}C NMR (100 MHz, $CDCl_3$)

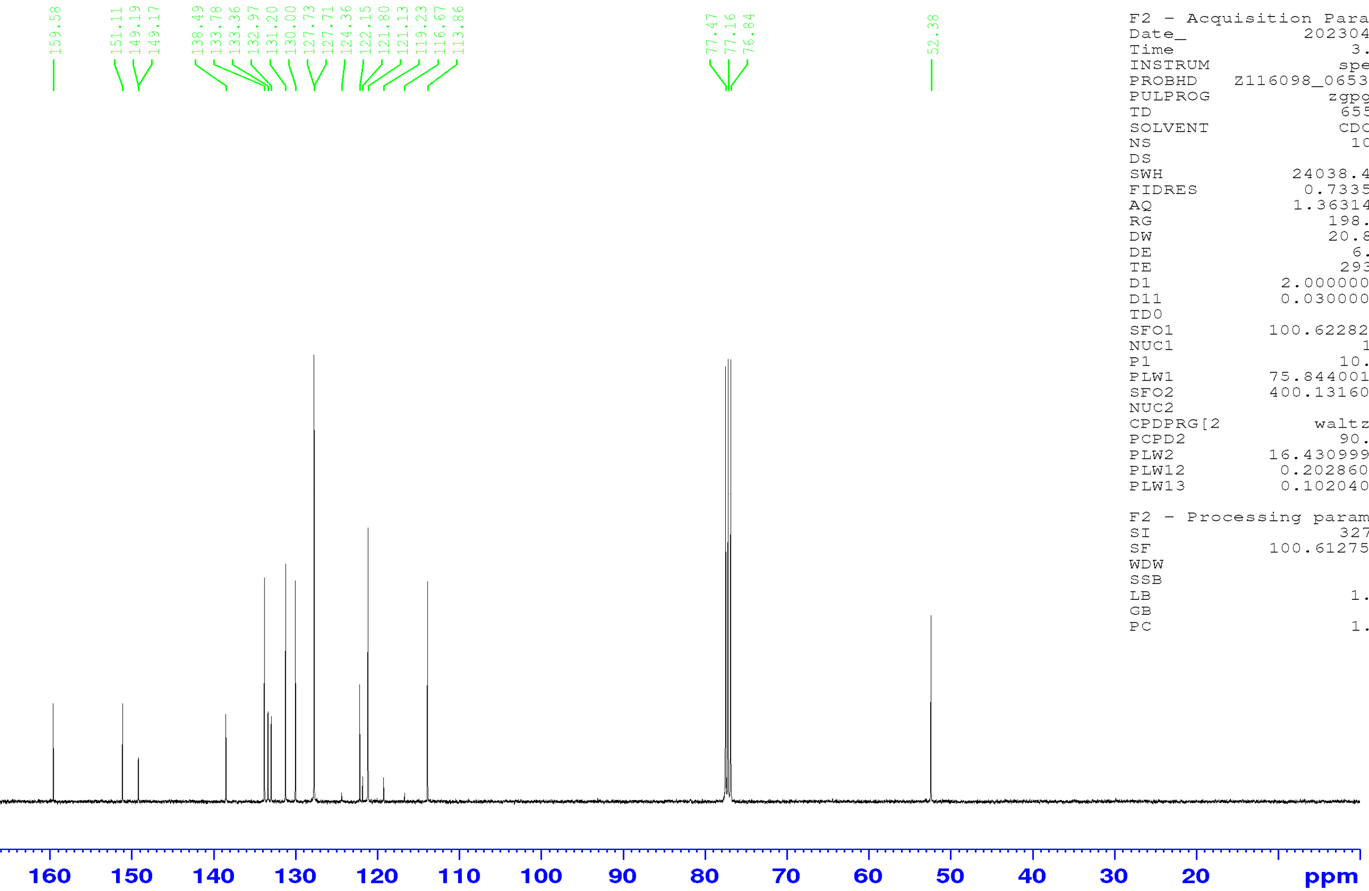


```
Current Data Parameters
NAME        ztc-20230407-6
EXPNO                    2
PROCNO                   1

F2 - Acquisition Parameters
Date_             20230408
Time                  3.54 h
INSTRUM              spect
PROBHD      Z116098_0653 (
PULPROG            zgpg30
TD                  65536
SOLVENT             CDCl3
NS                   1024
DS                      4
SWH             24038.461 Hz
FIDRES           0.733596 Hz
AQ              1.3631488 sec
RG                 198.36
DW                 20.800 usec
DE                   6.50 usec
TE                  293.1 K
D1             2.00000000 sec
D11            0.03000000 sec
TD0                     1
SFO1          100.6228298 MHz
NUC1                  13C
P1                  10.00 usec
PLW1          75.84400177 W
SFO2          400.1316005 MHz
NUC2                   1H
CPDPRG[2          waltz16
PCPD2               90.00 usec
PLW2          16.43099976 W
PLW12          0.20286000 W
PLW13          0.10204000 W

F2 - Processing parameters
SI                  32768
SF            100.6127592 MHz
WDW                    EM
SSB                     0
LB                   1.00 Hz
GB                      0
PC                   1.40
```

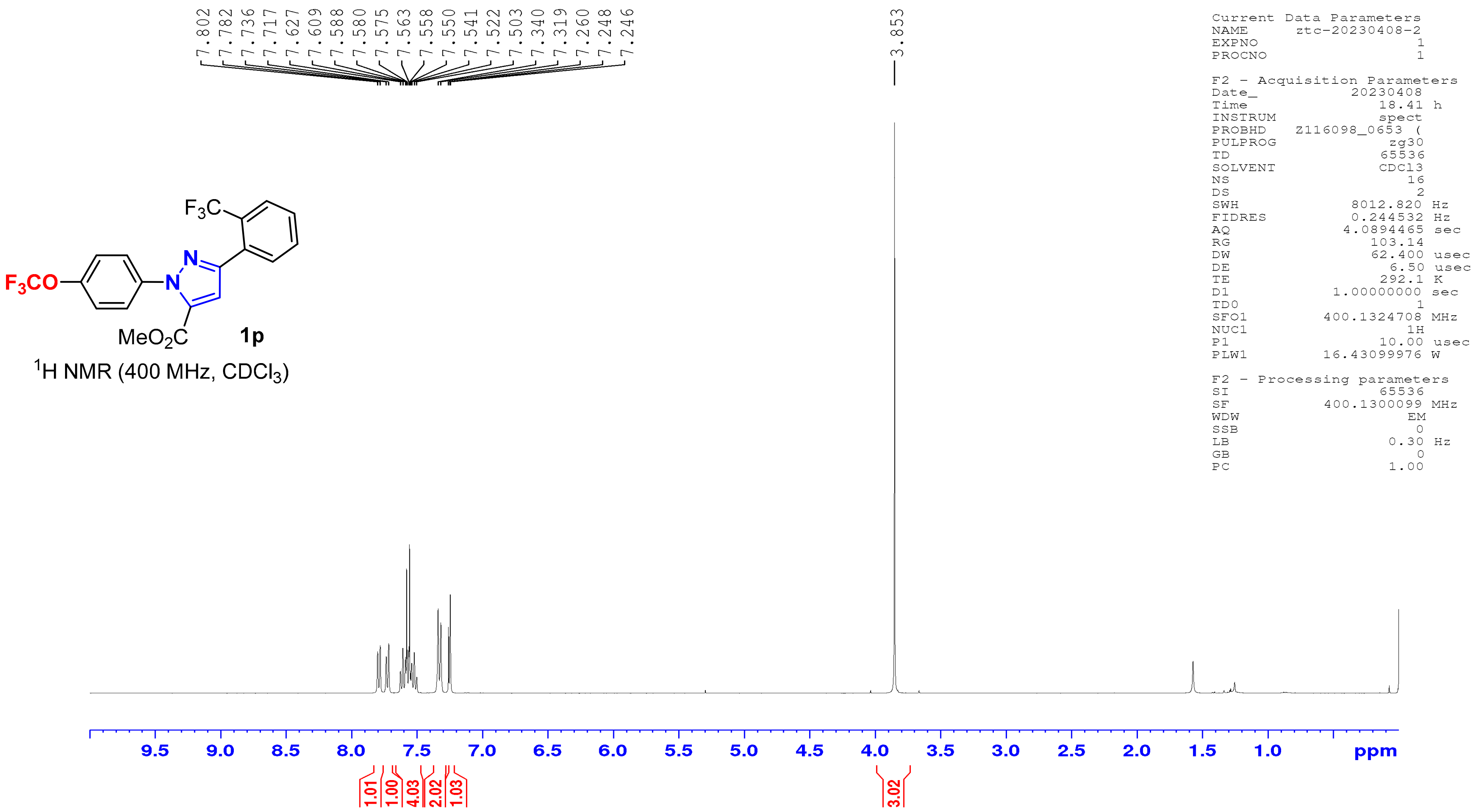

7.802
7.782
7.736
7.717
7.627
7.609
7.588
7.580
7.575
7.563
7.558
7.550
7.541
7.522
7.503
7.340
7.319
7.260
7.248
7.246
3.853
F3C
N
N
F3CO
MeO2C
1p
1H NMR (400 MHz, CDCl3)
Current Data Parameters
NAME ztc-20230408-2
EXPNO 1
PROCNO 1
F2 - Acquisition Parameters
Date_ 20230408
Time 18.41 h
INSTRUM spect
PROBHD Z116098_0653 (
PULPROG zg30
TD 65536
SOLVENT CDCl3
NS 16
DS 2
SWH 8012.820 Hz
FIDRES 0.244532 Hz
AQ 4.0894465 sec
RG 103.14
DW 62.400 usec
DE 6.50 usec
TE 292.1 K
D1 1.00000000 sec
TD0 1
SFO1 400.1324708 MHz
NUC1 1H
P1 10.00 usec
PLW1 16.43099976 W
F2 - Processing parameters
SI 65536
SF 400.1300099 MHz
WDW EM
SSB 0
LB 0.30 Hz
GB 0
PC 1.00
9.5
9.0
8.5
8.0
7.5
7.0
6.5
6.0
5.5
5.0
4.5
4.0
3.5
3.0
2.5
2.0
1.5
1.0
ppm
1.01
1.00
4.03
2.02
1.03
3.02

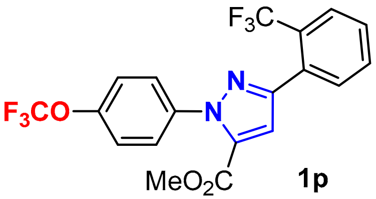

1p

^{13}C NMR (100 MHz, $CDCl_3$)

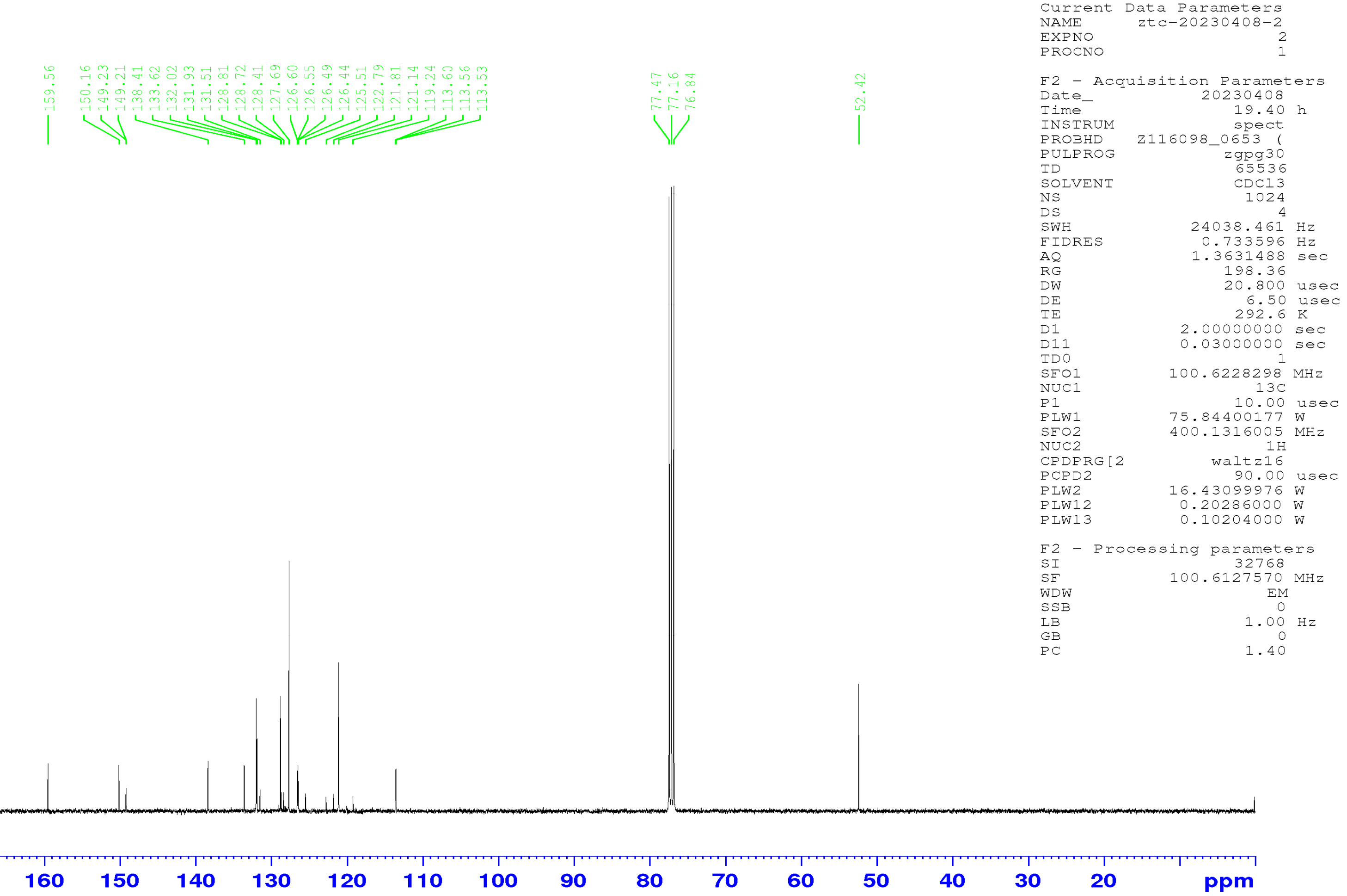

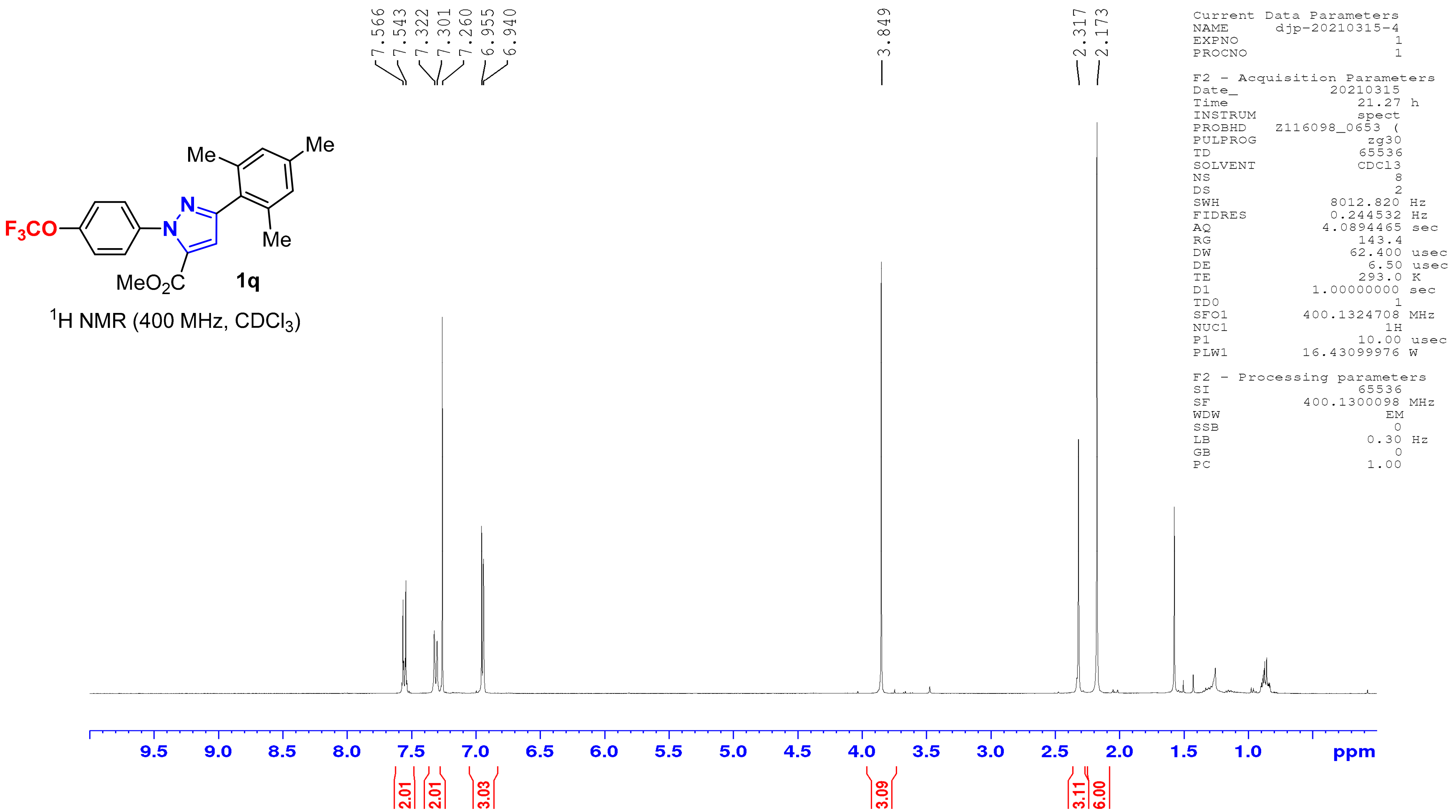
F3CO
N
N
Me
Me
Me
MeO2C
1q
1H NMR (400 MHz, CDCl3)
7.566
7.543
7.322
7.301
7.260
6.955
6.940
3.849
2.317
2.173
Current Data Parameters
NAME djp-20210315-4
EXPNO 1
PROCNO 1
F2 - Acquisition Parameters
Date_ 20210315
Time 21.27 h
INSTRUM spect
PROBHD Z116098_0653 (
PULPROG zg30
TD 65536
SOLVENT CDCl3
NS 8
DS 2
SWH 8012.820 Hz
FIDRES 0.244532 Hz
AQ 4.0894465 sec
RG 143.4
DW 62.400 usec
DE 6.50 usec
TE 293.0 K
D1 1.00000000 sec
TD0 1
SFO1 400.1324708 MHz
NUC1 1H
P1 10.00 usec
PLW1 16.43099976 W
F2 - Processing parameters
SI 65536
SF 400.1300098 MHz
WDW EM
SSB 0
LB 0.30 Hz
GB 0
PC 1.00
9.5
9.0
8.5
8.0
7.5
7.0
6.5
6.0
5.5
5.0
4.5
4.0
3.5
3.0
2.5
2.0
1.5
1.0
ppm
2.01
2.01
3.03
3.09
3.11
6.00

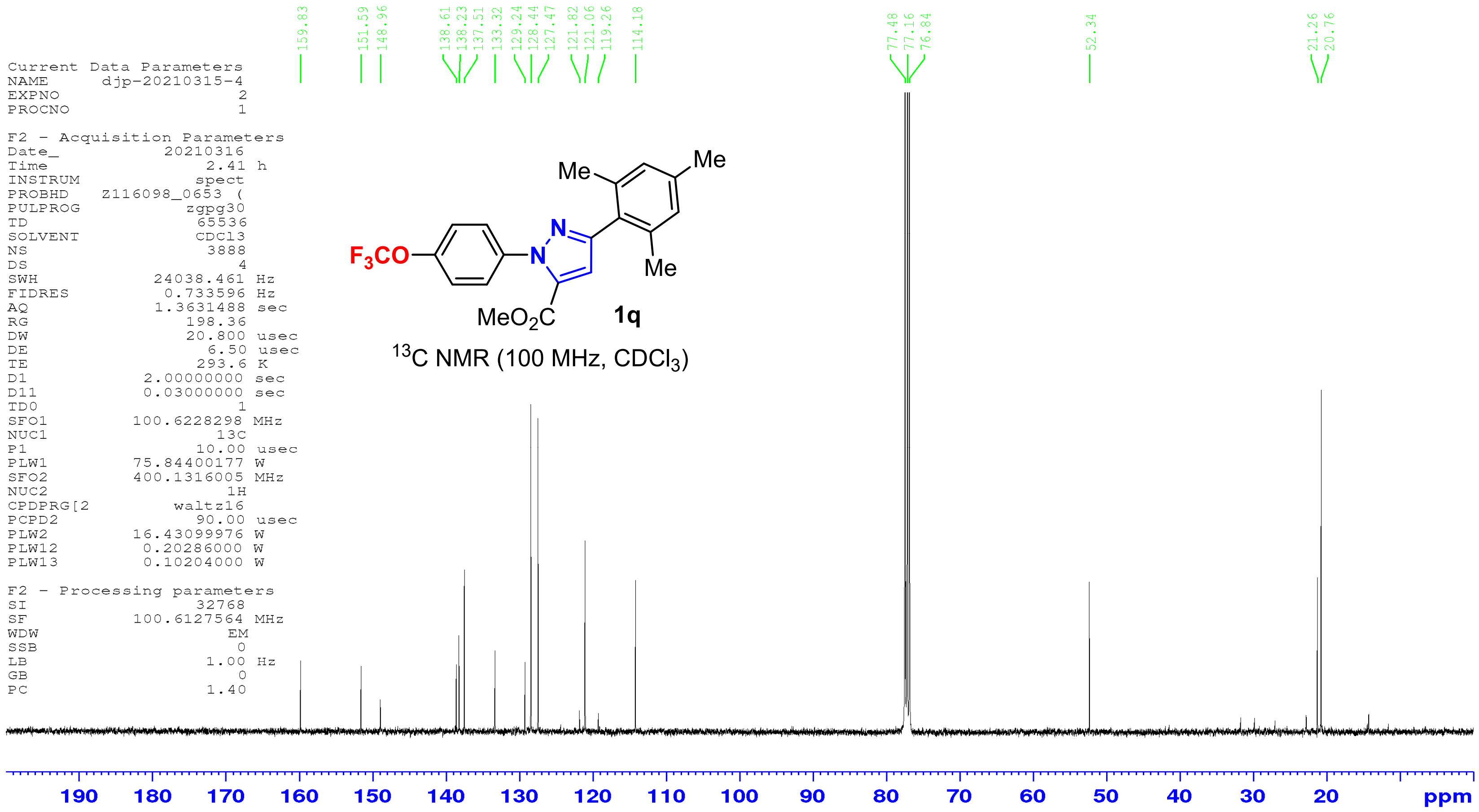
Current Data Parameters
NAME djp-20210315-4
EXPNO 2
PROCNO 1
F2 - Acquisition Parameters
Date_ 20210316
Time 2.41 h
INSTRUM spect
PROBHD Z116098_0653 (
PULPROG zgpg30
TD 65536
SOLVENT CDCl3
NS 3888
DS 4
SWH 24038.461 Hz
FIDRES 0.733596 Hz
AQ 1.3631488 sec
RG 198.36
DW 20.800 usec
DE 6.50 usec
TE 293.6 K
D1 2.00000000 sec
D11 0.03000000 sec
TD0 1
SFO1 100.6228298 MHz
NUC1 13C
P1 10.00 usec
PLW1 75.84400177 W
SFO2 400.1316005 MHz
NUC2 1H
CPDPRG[2 waltz16
PCPD2 90.00 usec
PLW2 16.43099976 W
PLW12 0.20286000 W
PLW13 0.10204000 W
F2 - Processing parameters
SI 32768
SF 100.6127564 MHz
WDW EM
SSB 0
LB 1.00 Hz
GB 0
PC 1.40
159.83
151.59
148.96
138.61
138.23
137.51
133.32
129.24
128.44
127.47
121.82
121.06
119.26
114.18
77.48
77.16
76.84
52.34
21.26
20.76
Me
Me
Me
F3CO
N
N
MeO2C
1q
13C NMR (100 MHz, CDCl3)
190
180
170
160
150
140
130
120
110
100
90
80
70
60
50
40
30
20
ppm

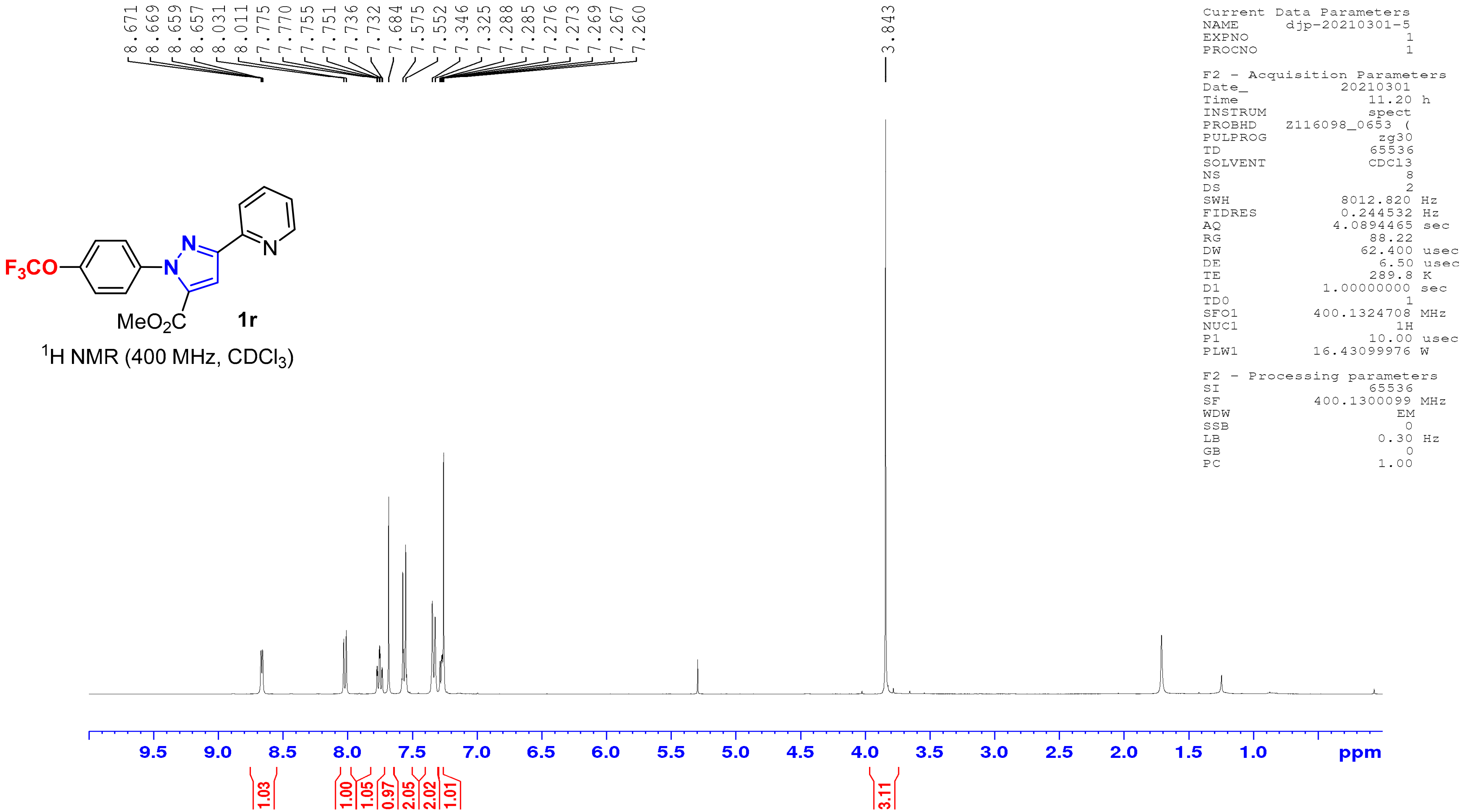

8.671
8.669
8.659
8.657
8.031
8.011
7.775
7.770
7.755
7.751
7.736
7.732
7.684
7.575
7.552
7.346
7.325
7.288
7.285
7.276
7.273
7.269
7.267
7.260
3.843
F3CO
N
N
N
MeO2C
1r
1H NMR (400 MHz, CDCl3)
Current Data Parameters
NAME djp-20210301-5
EXPNO 1
PROCNO 1
F2 - Acquisition Parameters
Date_ 20210301
Time 11.20 h
INSTRUM spect
PROBHD Z116098_0653 (
PULPROG zg30
TD 65536
SOLVENT CDCl3
NS 8
DS 2
SWH 8012.820 Hz
FIDRES 0.244532 Hz
AQ 4.0894465 sec
RG 88.22
DW 62.400 usec
DE 6.50 usec
TE 289.8 K
D1 1.00000000 sec
TD0 1
SFO1 400.1324708 MHz
NUC1 1H
P1 10.00 usec
PLW1 16.43099976 W
F2 - Processing parameters
SI 65536
SF 400.1300099 MHz
WDW EM
SSB 0
LB 0.30 Hz
GB 0
PC 1.00
9.5
9.0
8.5
8.0
7.5
7.0
6.5
6.0
5.5
5.0
4.5
4.0
3.5
3.0
2.5
2.0
1.5
1.0
ppm
1.03
1.00
1.05
0.97
2.05
2.02
1.01
3.11

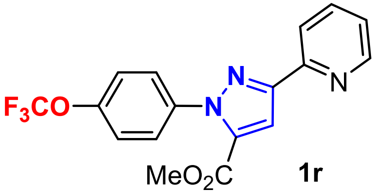

1r

^{13}C NMR (100 MHz, $CDCl_3$)

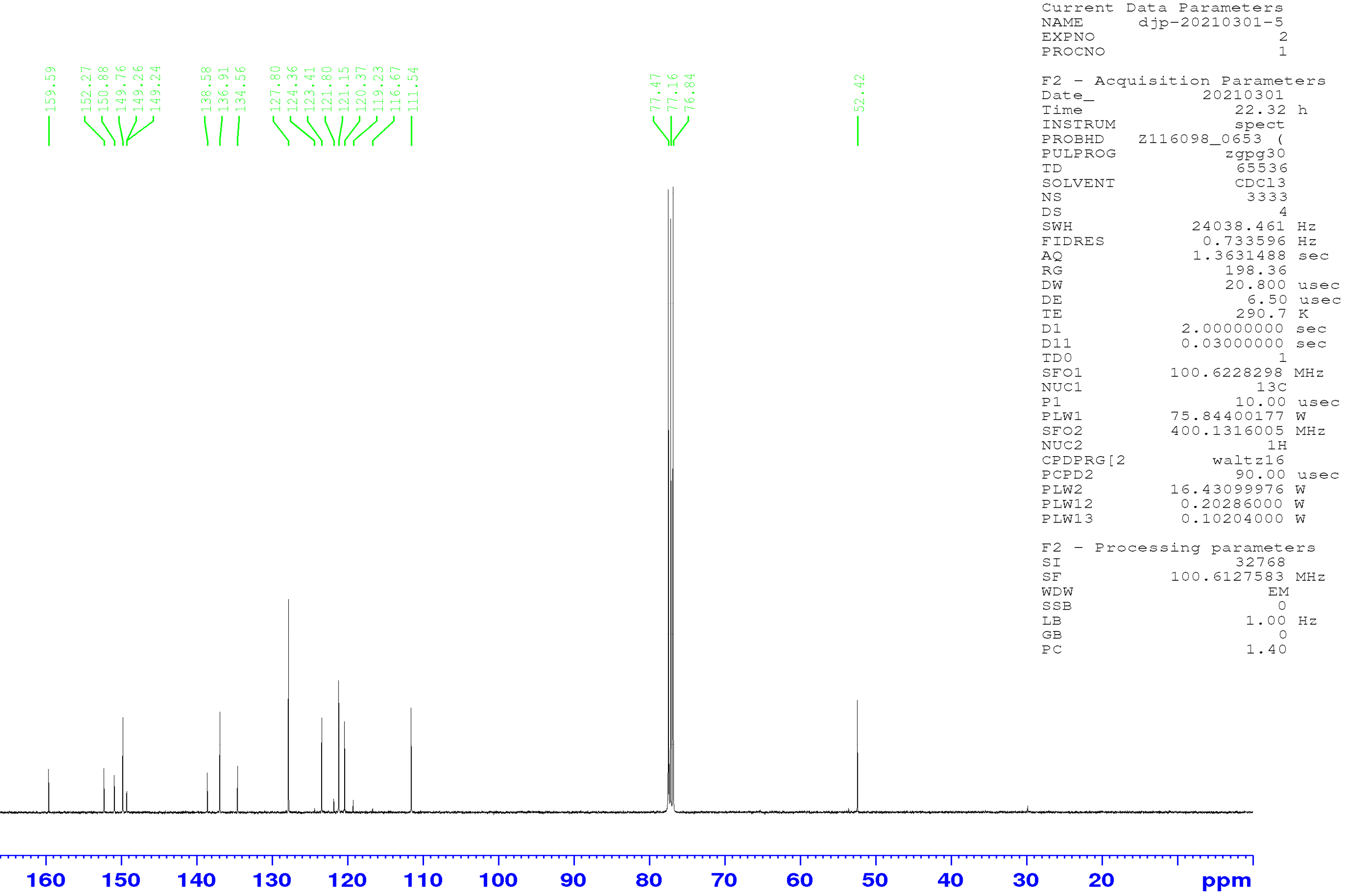

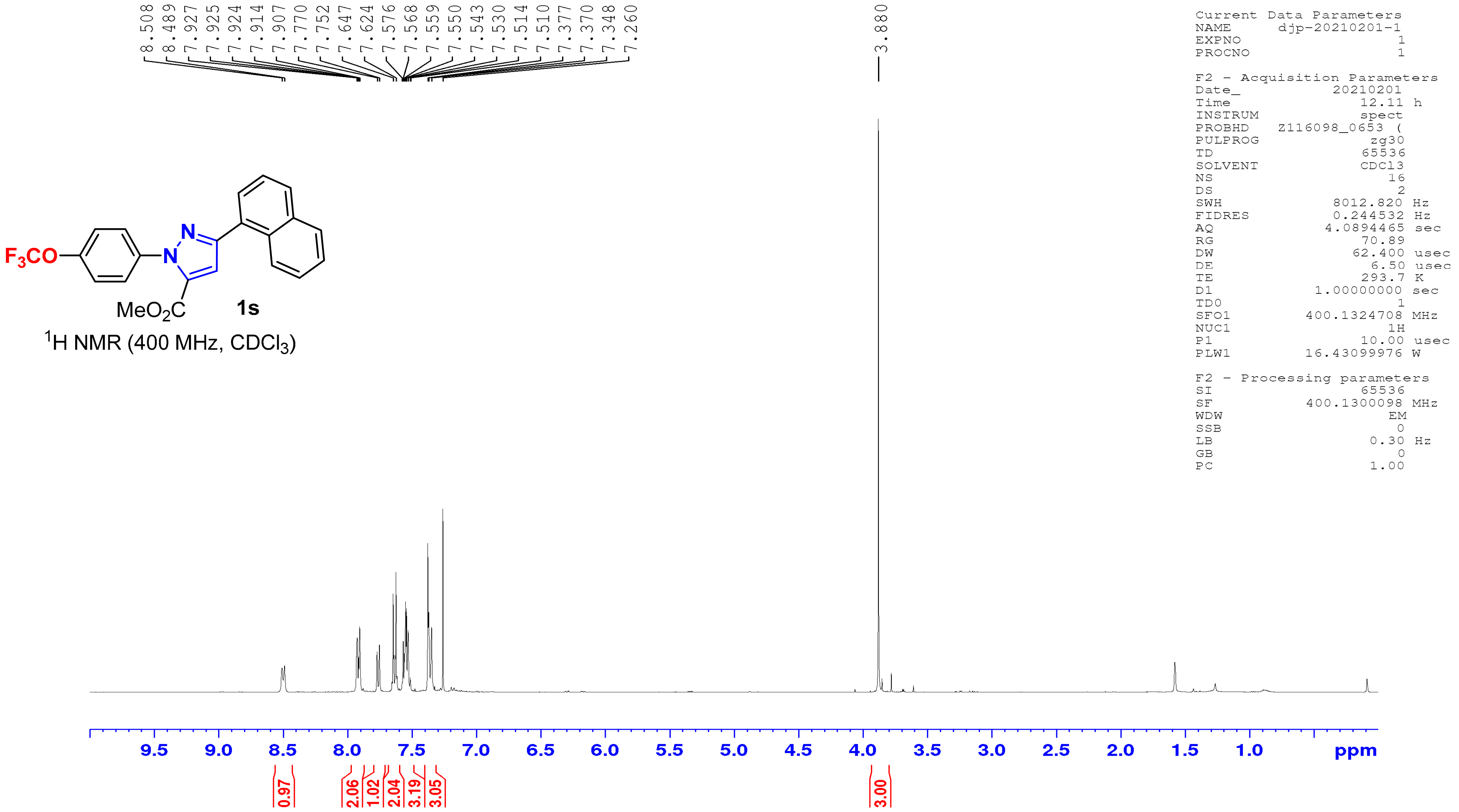

1s

^{1}H NMR (400 MHz, $CDCl_3$)

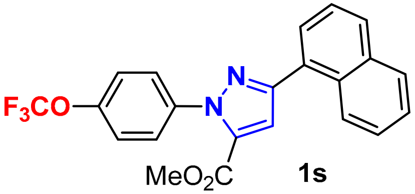

1s

^{13}C NMR (100 MHz, $CDCl_3$)

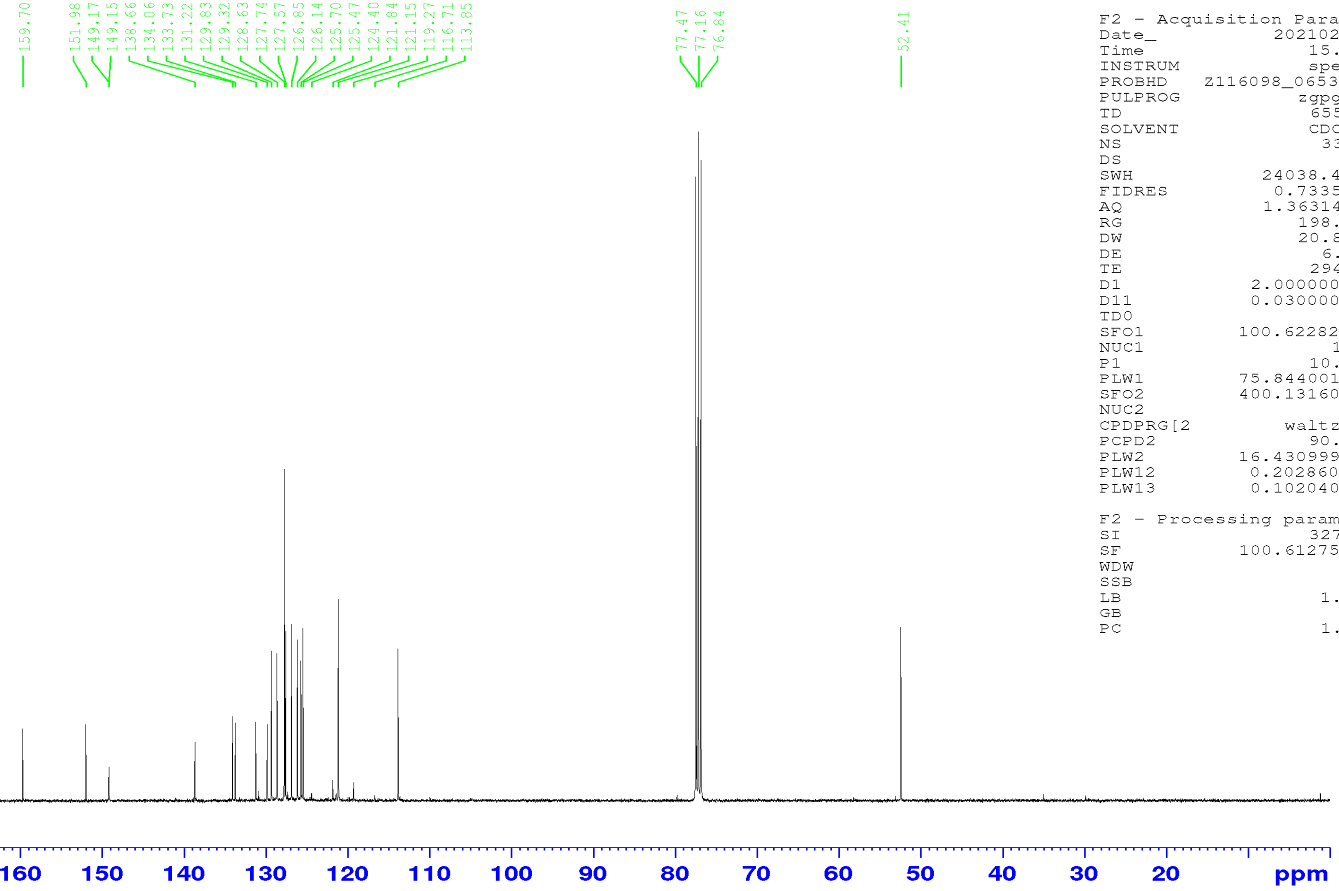


```
Current Data Parameters
NAME         djp-20210201-1
EXPNO                     2
PROCNO                    1

F2 - Acquisition Parameters
Date_              20210201
Time                 15.45 h
INSTRUM               spect
PROBHD     Z116098_0653 (
PULPROG             zgpg30
TD                   65536
SOLVENT              CDCl3
NS                    3333
DS                       4
SWH              24038.461 Hz
FIDRES            0.733596 Hz
AQ               1.3631488 sec
RG                  198.36
DW                  20.800 usec
DE                    6.50 usec
TE                   294.6 K
D1              2.00000000 sec
D11             0.03000000 sec
TD0                      1
SFO1           100.6228298 MHz
NUC1                   13C
P1                   10.00 usec
PLW1           75.84400177 W
SFO2           400.1316005 MHz
NUC2                    1H
CPDPRG[2           waltz16
PCPD2                90.00 usec
PLW2           16.43099976 W
PLW12           0.20286000 W
PLW13           0.10204000 W

F2 - Processing parameters
SI                   32768
SF             100.6127579 MHz
WDW                     EM
SSB                      0
LB                    1.00 Hz
GB                       0
PC                    1.40
```

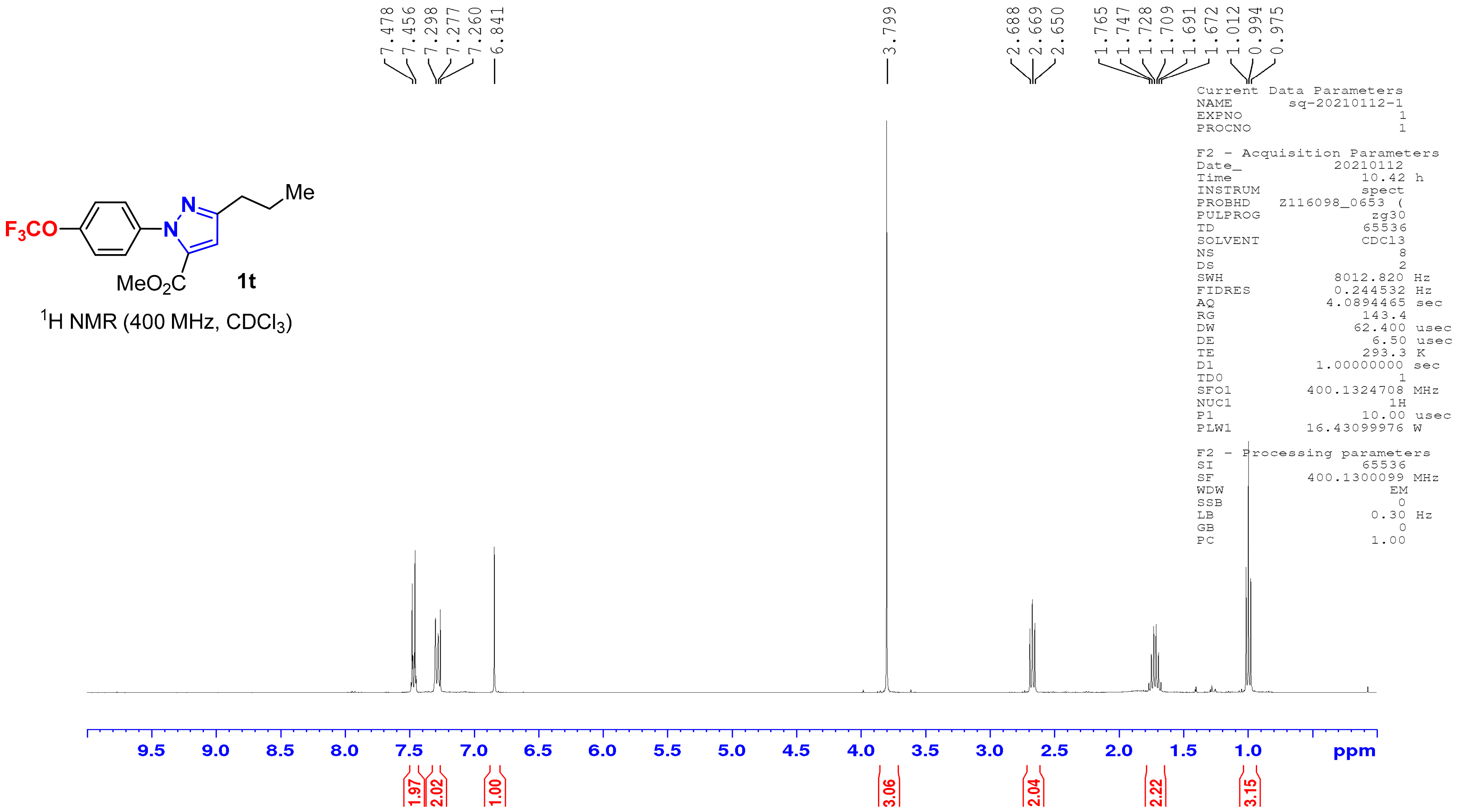

1t

^{1}H NMR (400 MHz, $CDCl_3$)

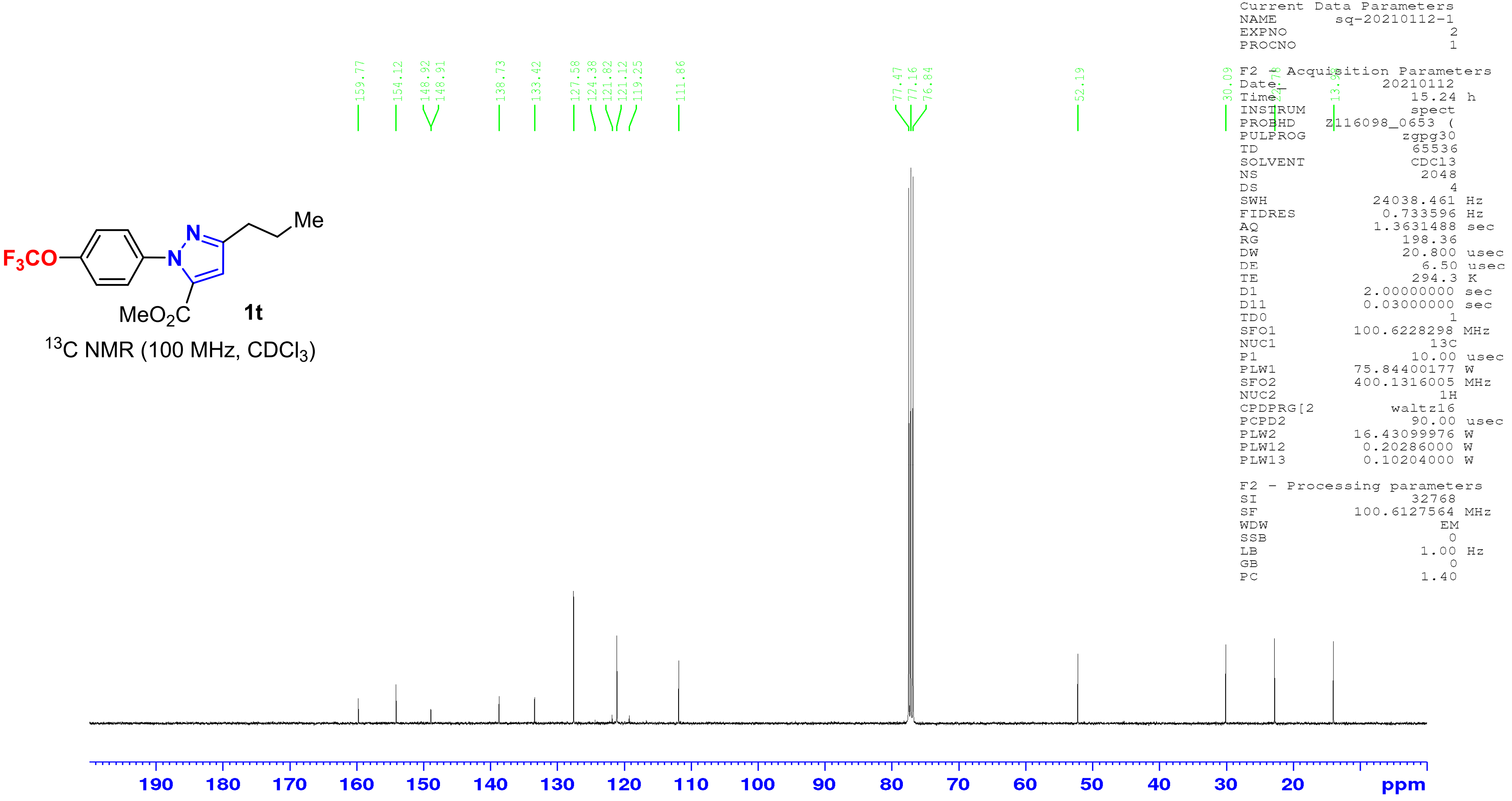

1t

^{13}C NMR (100 MHz, $CDCl_3$)

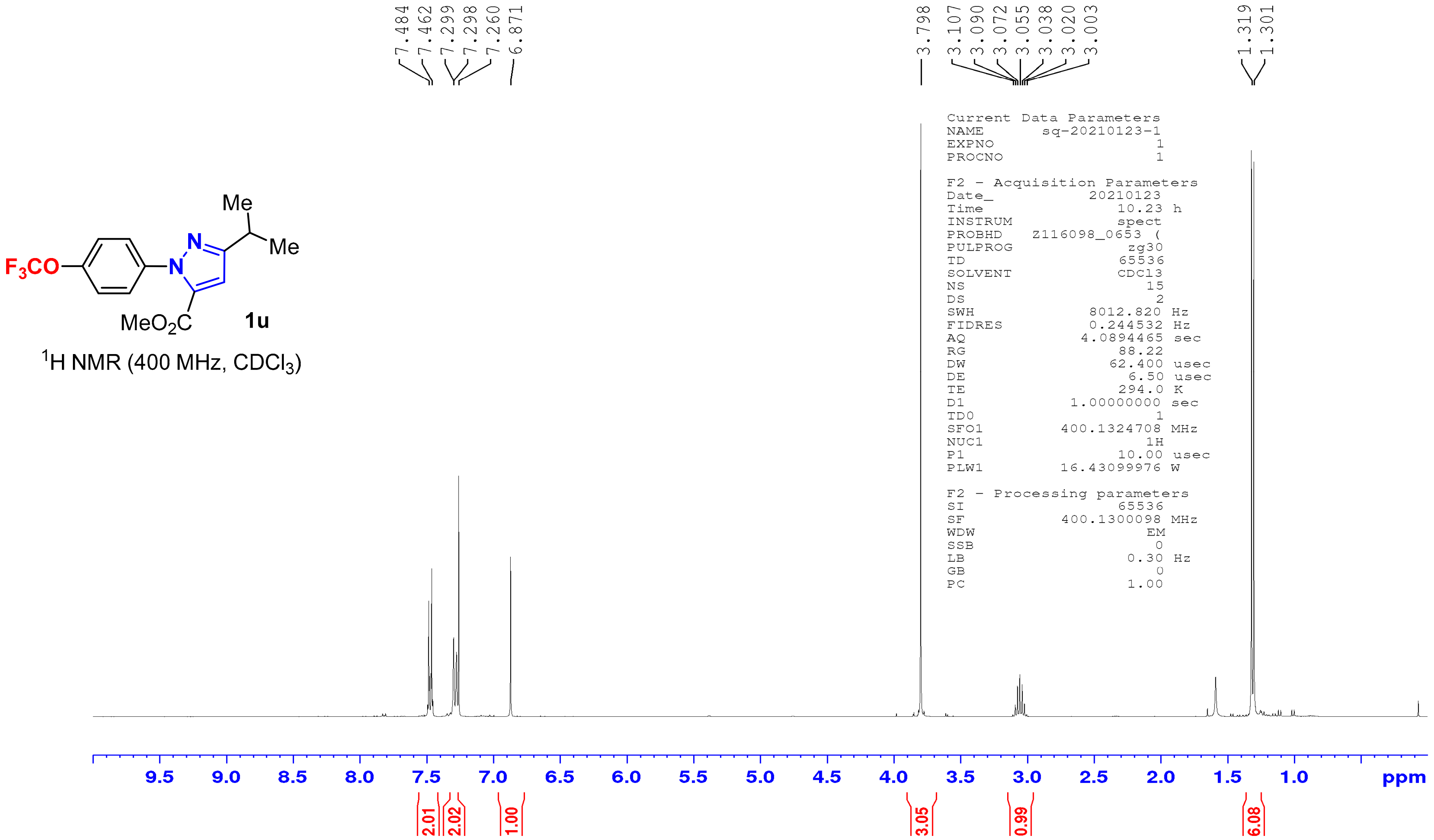

1u

^{1}H NMR (400 MHz, $CDCl_3$)

Current Data Parameters
NAME djp-20210729-6
EXPNO 2
PROCNO 1

F2 - Acquisition Parameters
Date_ 20210730
Time 0.39 h
INSTRUM spect
PROBHD Z116098_0653 (
PULPROG zgpg30
TD 65536
SOLVENT CDCl3
NS 2048
DS 4
SWH 24038.461 Hz
FIDRES 0.733596 Hz
AQ 1.3631488 sec
RG 198.36
DW 20.800 usec
DE 6.50 usec
TE 294.0 K
D1 2.00000000 sec
D11 0.03000000 sec
TD0 1
SFO1 100.6228298 MHz
NUC1 13C
P1 10.00 usec
PLW1 75.84400177 W
SFO2 400.1316005 MHz
NUC2 1H
CPDPRG[2 waltz16
PCPD2 90.00 usec
PLW2 16.43099976 W
PLW12 0.20286000 W
PLW13 0.10204000 W

F2 - Processing parameters
SI 32768
SF 100.6127564 MHz
WDW EM
SSB 0
LB 1.00 Hz
GB 0
PC 1.40

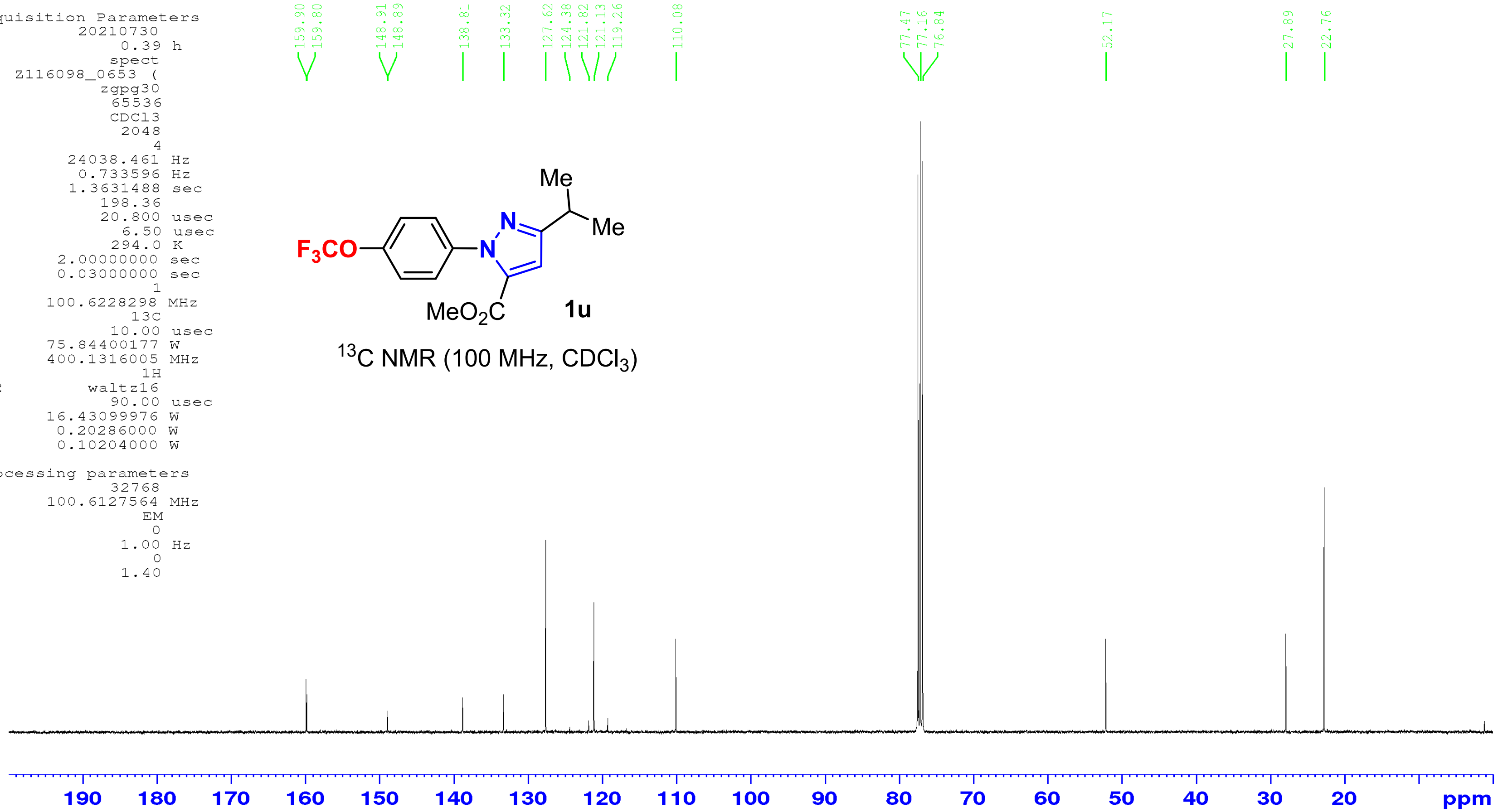

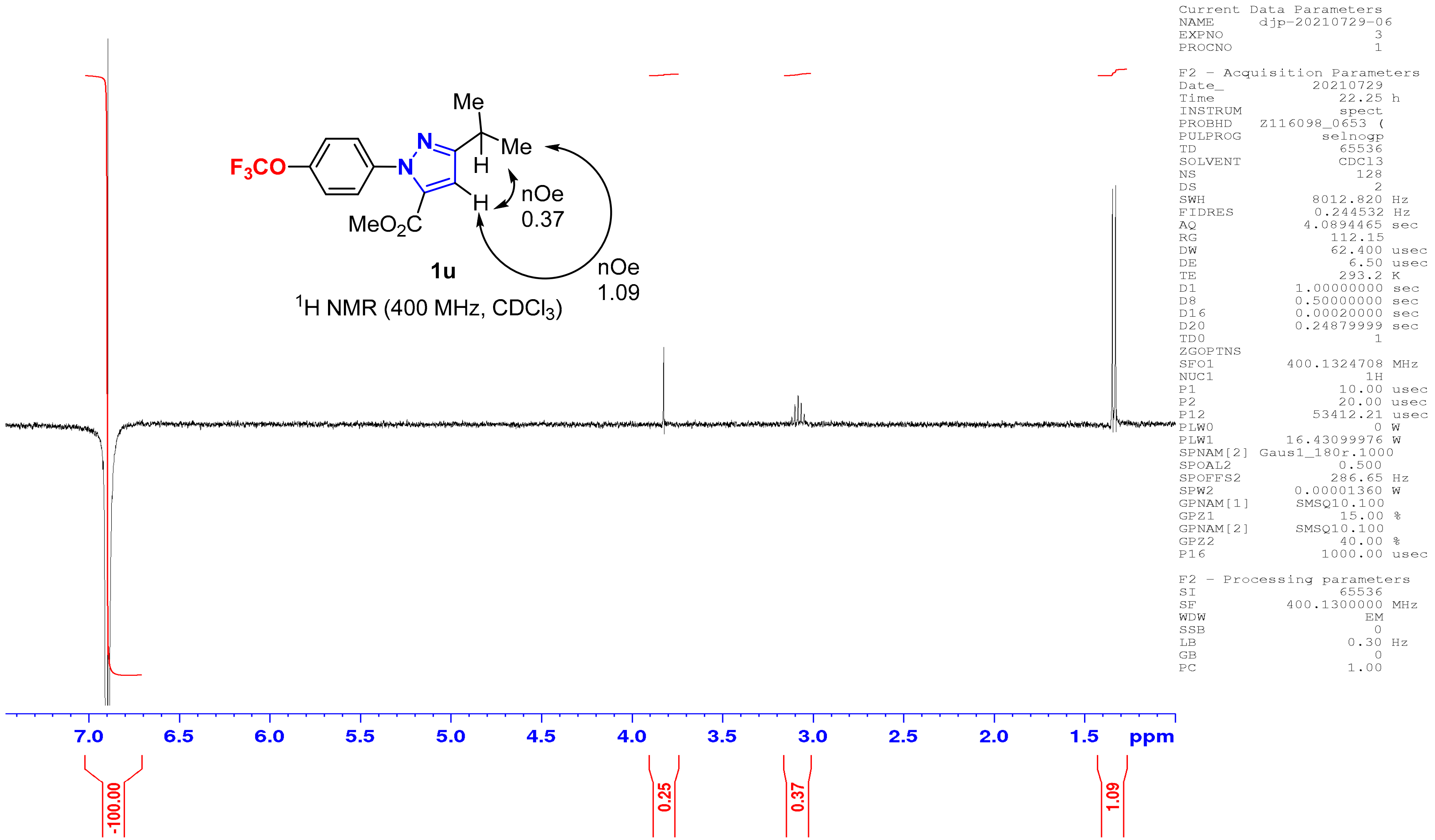
F3CO
N
N
Me
Me
H
H
nOe
0.37
nOe
1.09
MeO2C
1u
1H NMR (400 MHz, CDCl3)
7.0
6.5
6.0
5.5
5.0
4.5
4.0
3.5
3.0
2.5
2.0
1.5
ppm
-100.00
0.25
0.37
1.09
Current Data Parameters
NAME djp-20210729-06
EXPNO 3
PROCNO 1
F2 - Acquisition Parameters
Date_ 20210729
Time 22.25 h
INSTRUM spect
PROBHD Z116098_0653 (
PULPROG selnogp
TD 65536
SOLVENT CDCl3
NS 128
DS 2
SWH 8012.820 Hz
FIDRES 0.244532 Hz
AQ 4.0894465 sec
RG 112.15
DW 62.400 usec
DE 6.50 usec
TE 293.2 K
D1 1.00000000 sec
D8 0.50000000 sec
D16 0.00020000 sec
D20 0.24879999 sec
TD0 1
ZGOPTNS
SFO1 400.1324708 MHz
NUC1 1H
P1 10.00 usec
P2 20.00 usec
P12 53412.21 usec
PLW0 0 W
PLW1 16.43099976 W
SPNAM[2] Gaus1_180r.1000
SPOAL2 0.500
SPOFFS2 286.65 Hz
SPW2 0.00001360 W
GPNAM[1] SMSQ10.100
GPZ1 15.00 %
GPNAM[2] SMSQ10.100
GPZ2 40.00 %
P16 1000.00 usec
F2 - Processing parameters
SI 65536
SF 400.1300000 MHz
WDW EM
SSB 0
LB 0.30 Hz
GB 0
PC 1.00

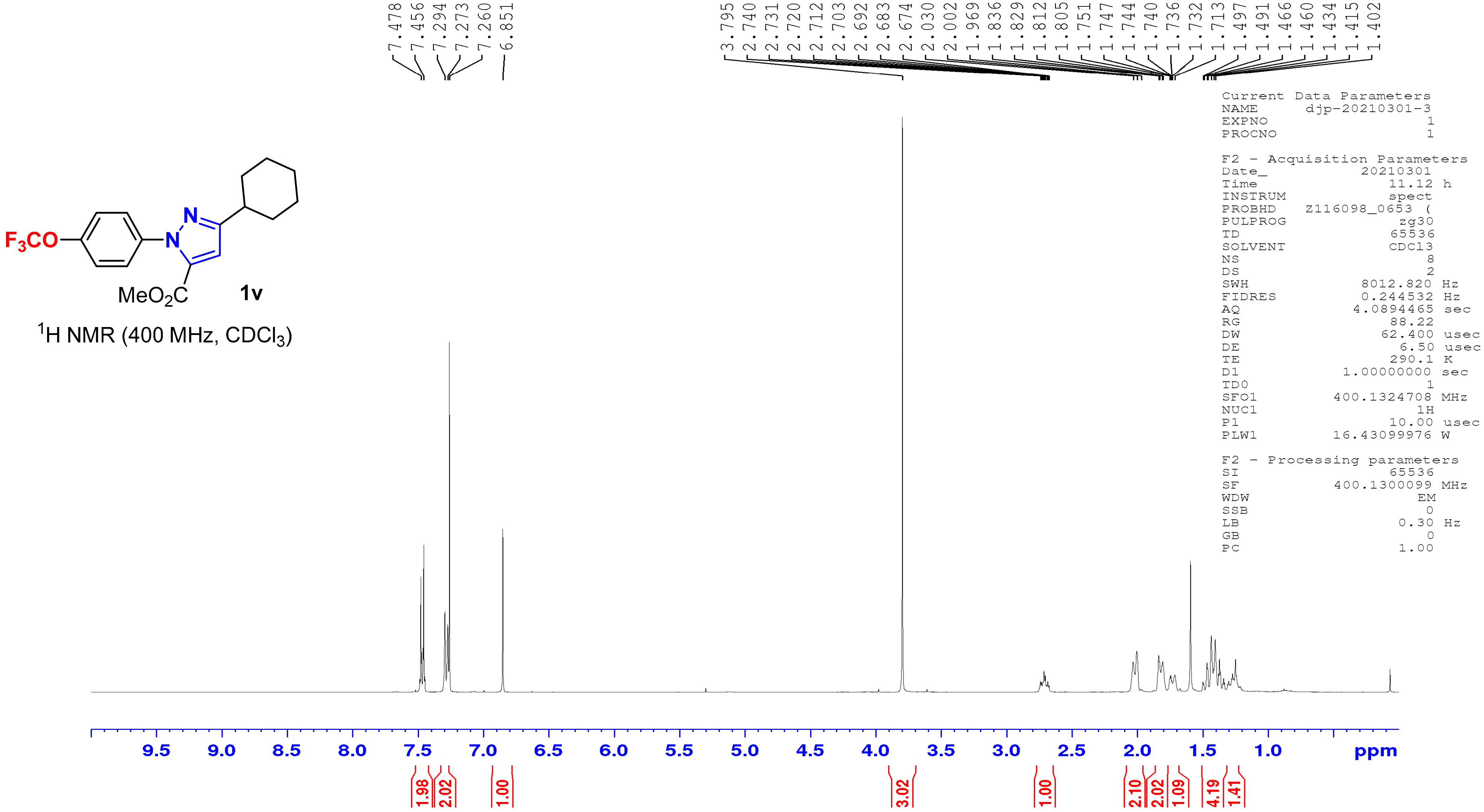
7.478
7.456
7.294
7.273
7.260
6.851
3.795
2.740
2.731
2.720
2.712
2.703
2.692
2.683
2.674
2.030
2.002
1.969
1.836
1.829
1.812
1.805
1.751
1.747
1.744
1.740
1.736
1.732
1.713
1.497
1.491
1.466
1.460
1.434
1.415
1.402
F3CO
N
N
MeO2C
1v
1H NMR (400 MHz, CDCl3)
Current Data Parameters
NAME djp-20210301-3
EXPNO 1
PROCNO 1
F2 - Acquisition Parameters
Date_ 20210301
Time 11.12 h
INSTRUM spect
PROBHD Z116098_0653 (
PULPROG zg30
TD 65536
SOLVENT CDCl3
NS 8
DS 2
SWH 8012.820 Hz
FIDRES 0.244532 Hz
AQ 4.0894465 sec
RG 88.22
DW 62.400 usec
DE 6.50 usec
TE 290.1 K
D1 1.00000000 sec
TD0 1
SFO1 400.1324708 MHz
NUC1 1H
P1 10.00 usec
PLW1 16.43099976 W
F2 - Processing parameters
SI 65536
SF 400.1300099 MHz
WDW EM
SSB 0
LB 0.30 Hz
GB 0
PC 1.00
9.5
9.0
8.5
8.0
7.5
7.0
6.5
6.0
5.5
5.0
4.5
4.0
3.5
3.0
2.5
2.0
1.5
1.0
ppm
1.98
2.02
1.00
3.02
1.00
2.10
2.02
1.09
4.19
1.41

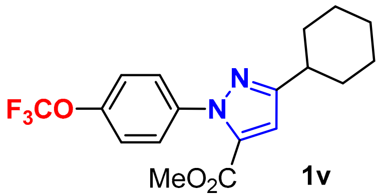

1v

^{13}C NMR (100 MHz, $CDCl_3$)

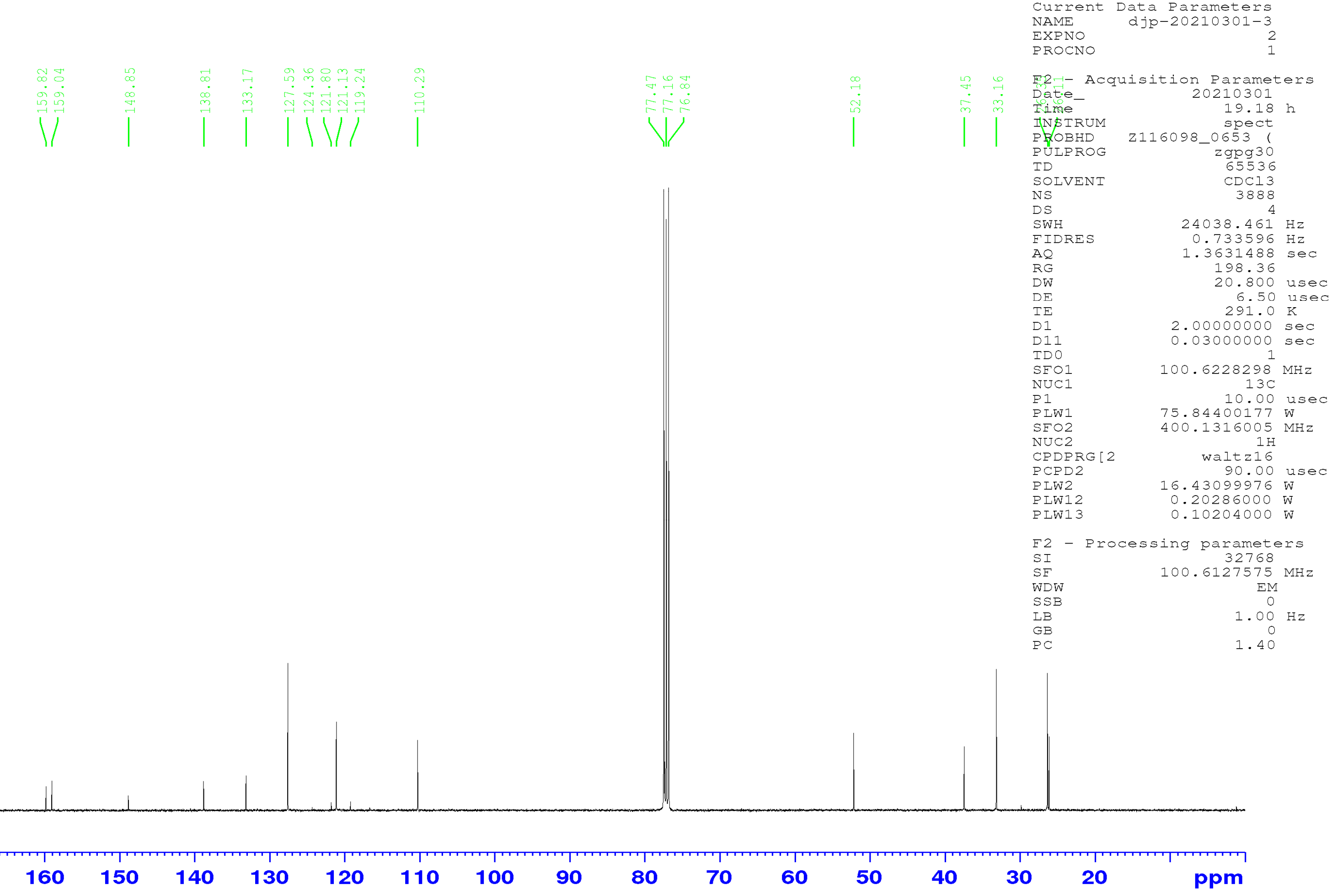

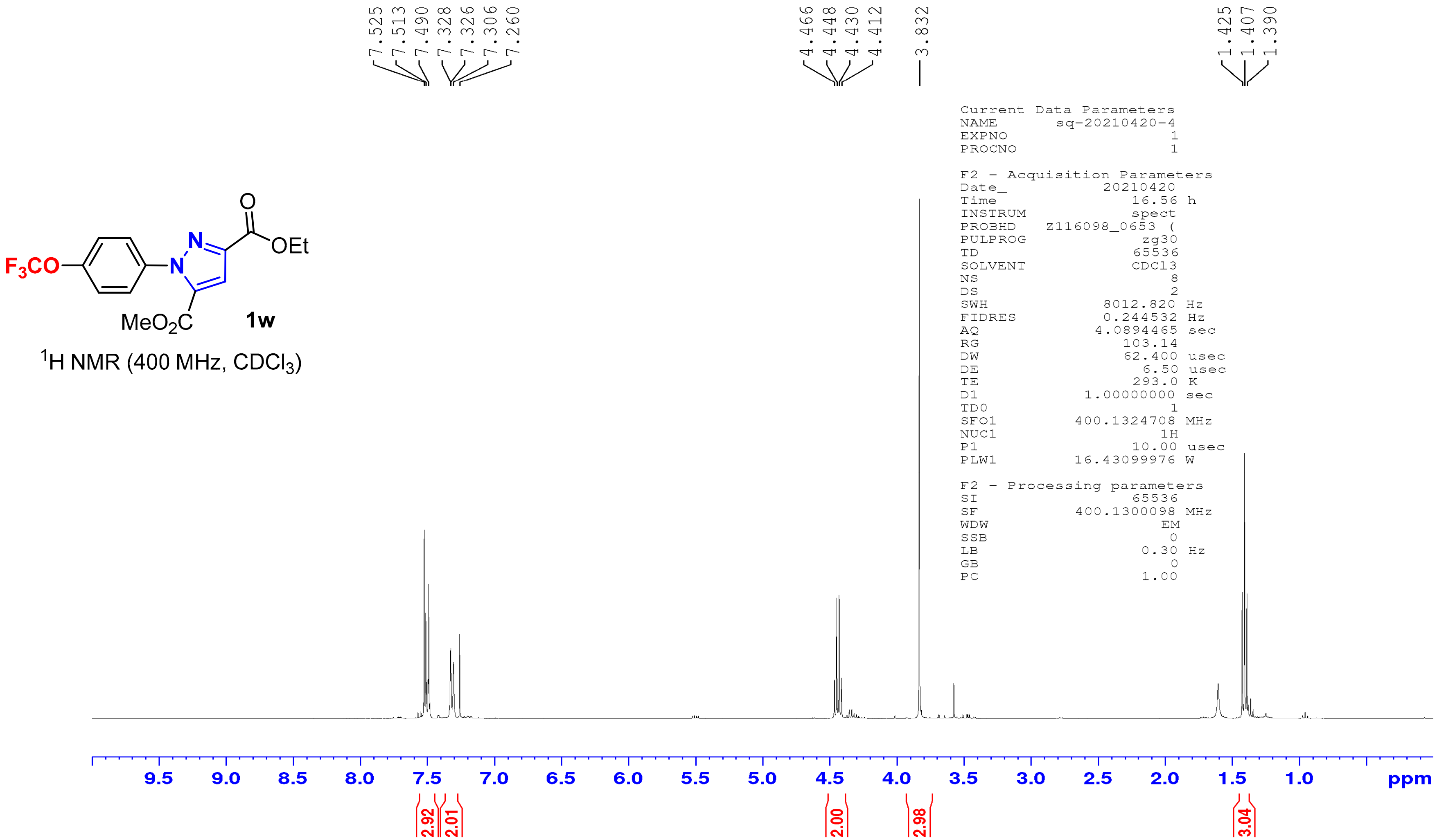

7.525
7.513
7.490
7.328
7.326
7.306
7.260
4.466
4.448
4.430
4.412
3.832
1.425
1.407
1.390
F3CO
N
N
O
OEt
MeO2C
1w
1H NMR (400 MHz, CDCl3)
Current Data Parameters
NAME sq-20210420-4
EXPNO 1
PROCNO 1
F2 - Acquisition Parameters
Date_ 20210420
Time 16.56 h
INSTRUM spect
PROBHD Z116098_0653 (
PULPROG zg30
TD 65536
SOLVENT CDCl3
NS 8
DS 2
SWH 8012.820 Hz
FIDRES 0.244532 Hz
AQ 4.0894465 sec
RG 103.14
DW 62.400 usec
DE 6.50 usec
TE 293.0 K
D1 1.00000000 sec
TD0 1
SFO1 400.1324708 MHz
NUC1 1H
P1 10.00 usec
PLW1 16.43099976 W
F2 - Processing parameters
SI 65536
SF 400.1300098 MHz
WDW EM
SSB 0
LB 0.30 Hz
GB 0
PC 1.00
9.5 9.0 8.5 8.0 7.5 7.0 6.5 6.0 5.5 5.0 4.5 4.0 3.5 3.0 2.5 2.0 1.5 1.0 ppm
2.92
2.01
2.00
2.98
3.04

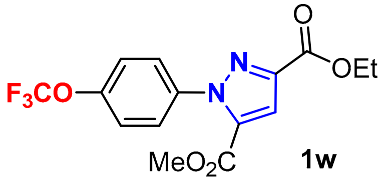

1w

^{13}C NMR (100 MHz, $CDCl_3$)

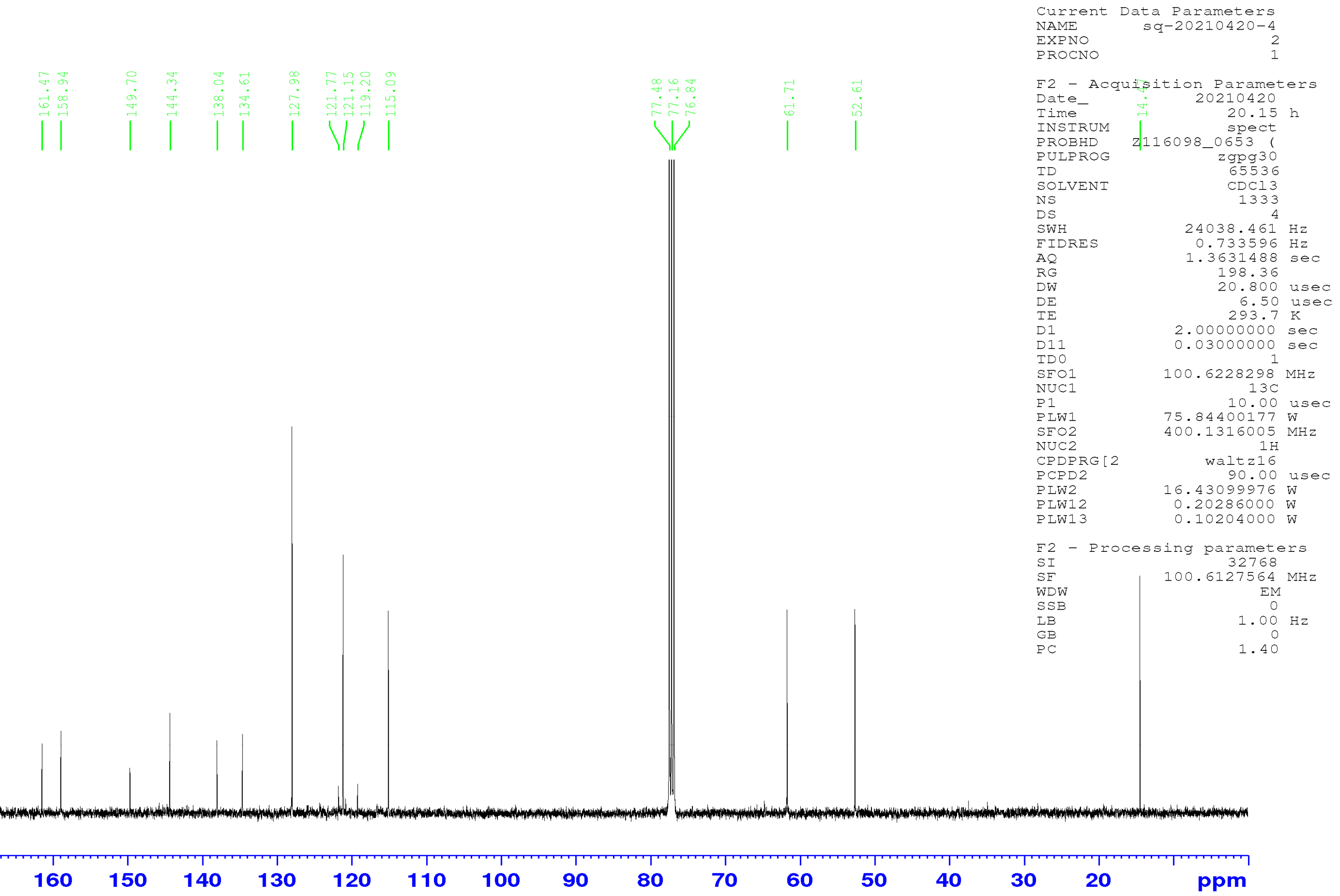

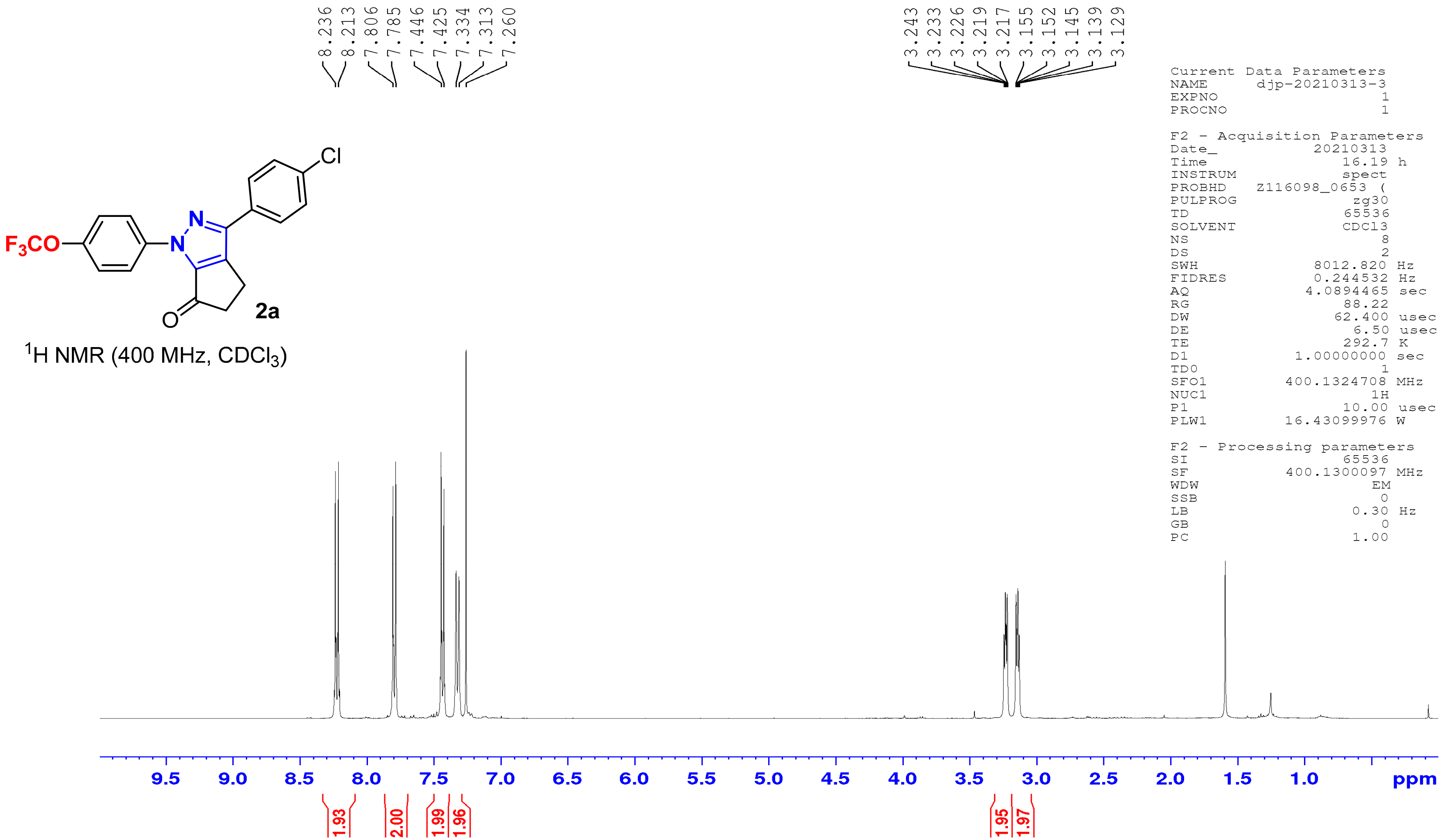

F3CO
N
N
Cl
O
2a
1H NMR (400 MHz, CDCl3)
8.236
8.213
7.806
7.785
7.446
7.425
7.334
7.313
7.260
3.243
3.233
3.226
3.219
3.217
3.155
3.152
3.145
3.139
3.129
Current Data Parameters
NAME djp-20210313-3
EXPNO 1
PROCNO 1
F2 - Acquisition Parameters
Date_ 20210313
Time 16.19 h
INSTRUM spect
PROBHD Z116098_0653 (
PULPROG zg30
TD 65536
SOLVENT CDCl3
NS 8
DS 2
SWH 8012.820 Hz
FIDRES 0.244532 Hz
AQ 4.0894465 sec
RG 88.22
DW 62.400 usec
DE 6.50 usec
TE 292.7 K
D1 1.00000000 sec
TD0 1
SFO1 400.1324708 MHz
NUC1 1H
P1 10.00 usec
PLW1 16.43099976 W
F2 - Processing parameters
SI 65536
SF 400.1300097 MHz
WDW EM
SSB 0
LB 0.30 Hz
GB 0
PC 1.00
9.5
9.0
8.5
8.0
7.5
7.0
6.5
6.0
5.5
5.0
4.5
4.0
3.5
3.0
2.5
2.0
1.5
1.0
ppm
1.93
2.00
1.99
1.96
1.95
1.97

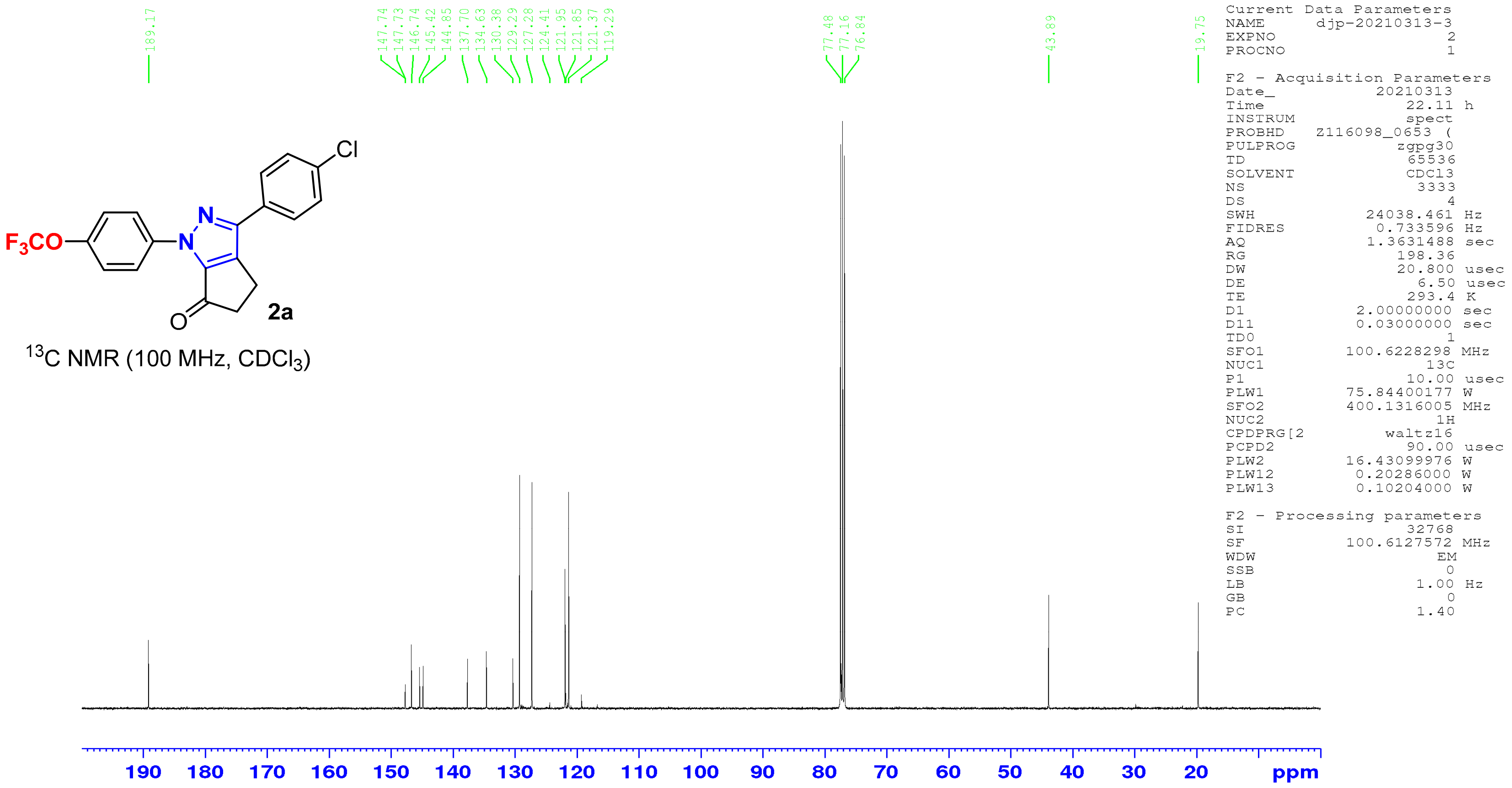

^{13}C NMR (100 MHz, $CDCl_3$)

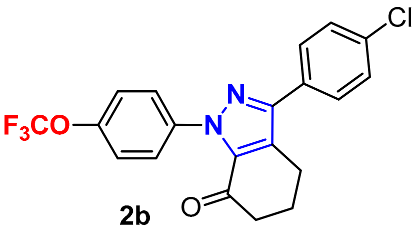

2b

^{1}H NMR (400 MHz, $CDCl_3$)

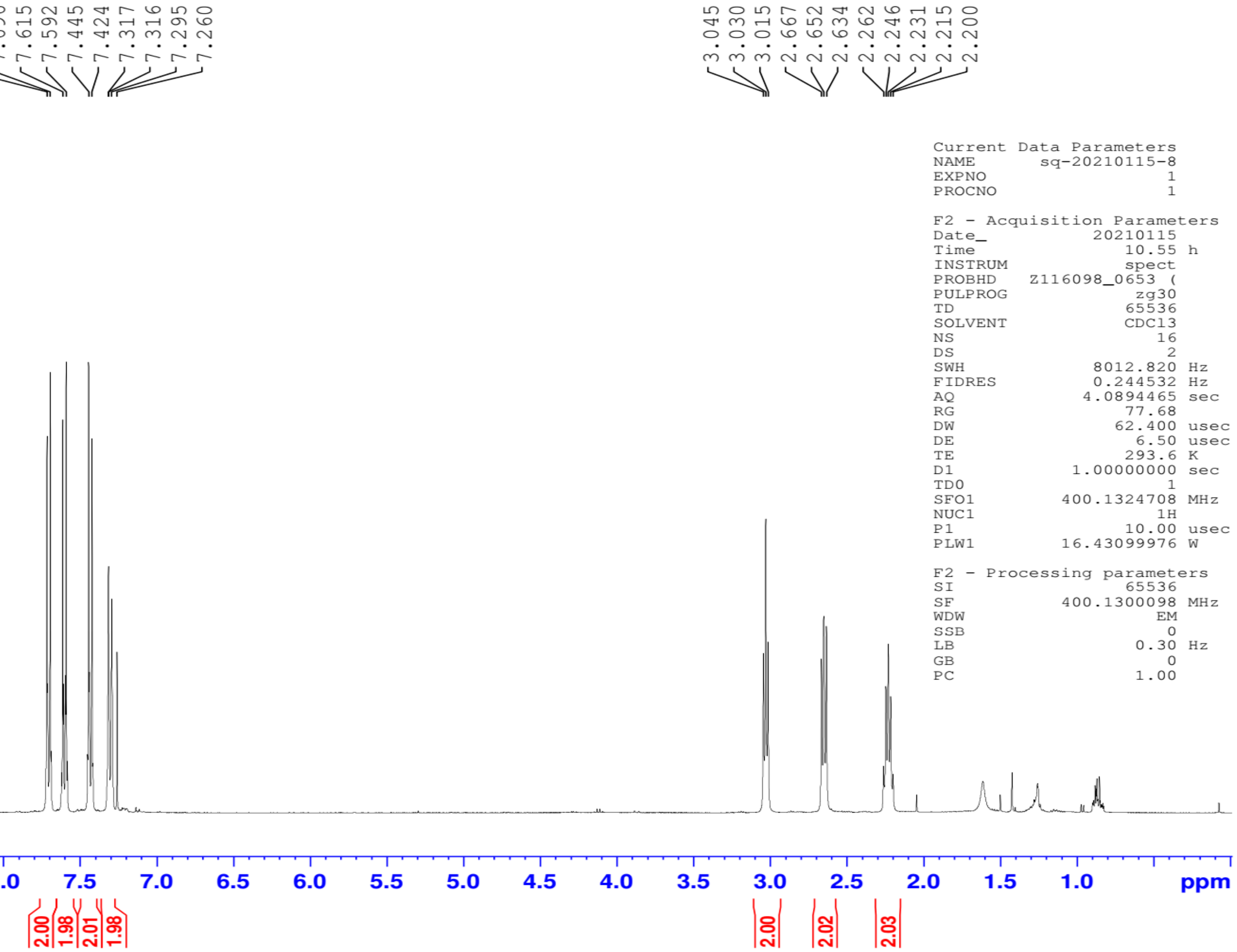

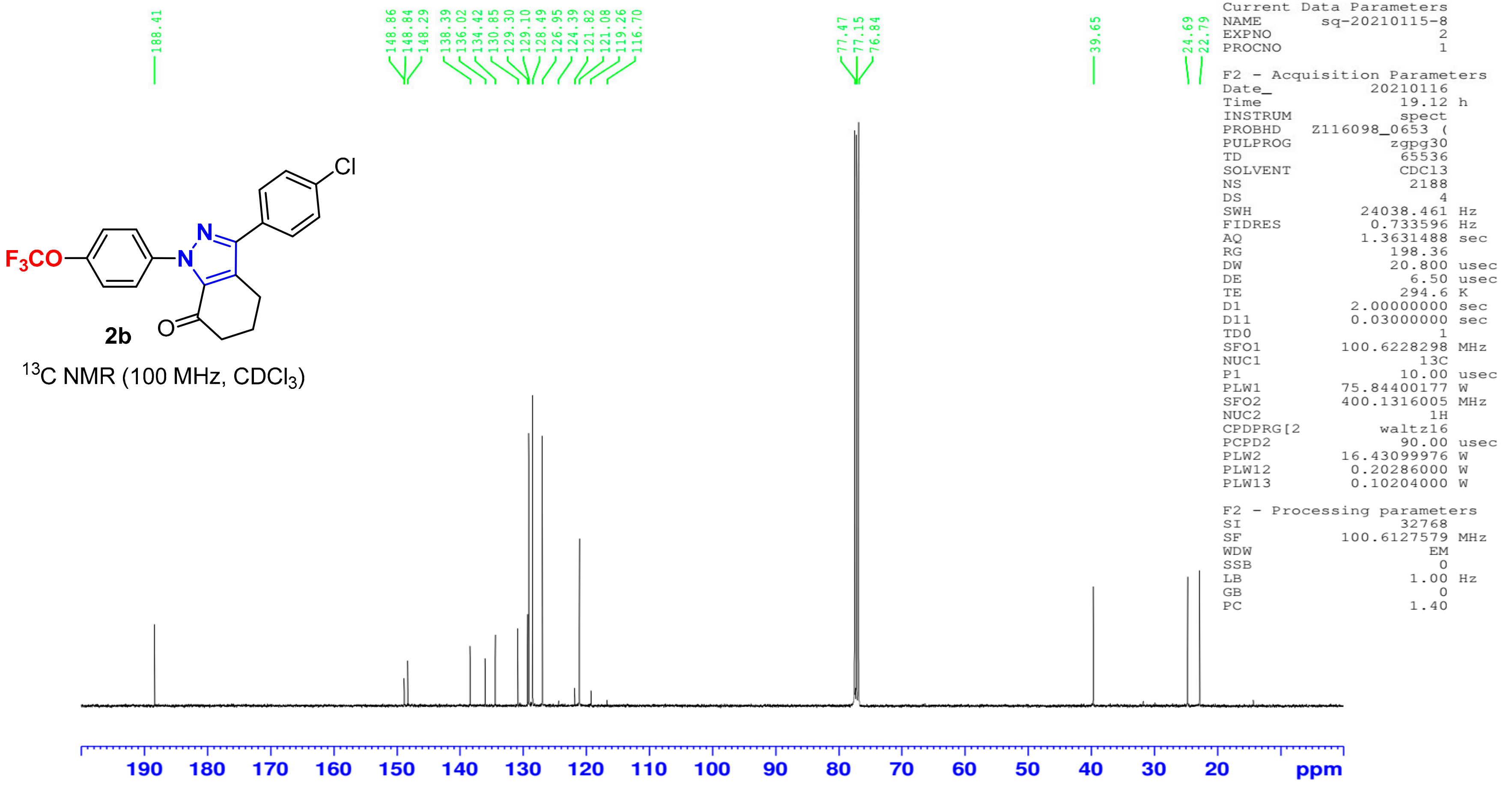

188.41
148.86
148.84
148.29
138.39
136.02
134.42
130.85
129.30
129.10
128.49
126.95
124.39
121.82
121.08
119.26
116.70
77.47
77.15
76.84
39.65
24.69
22.79
Cl
N
F_3CO
N
O
2b
^{13}C NMR (100 MHz, $CDCl_3$)
Current Data Parameters
NAME sq-20210115-8
EXPNO 2
PROCNO 1
F2 - Acquisition Parameters
Date_ 20210116
Time 19.12 h
INSTRUM spect
PROBHD Z116098_0653 (
PULPROG zgpg30
TD 65536
SOLVENT CDCl3
NS 2188
DS 4
SWH 24038.461 Hz
FIDRES 0.733596 Hz
AQ 1.3631488 sec
RG 198.36
DW 20.800 usec
DE 6.50 usec
TE 294.6 K
D1 2.00000000 sec
D11 0.03000000 sec
TD0 1
SFO1 100.6228298 MHz
NUC1 13C
P1 10.00 usec
PLW1 75.84400177 W
SFO2 400.1316005 MHz
NUC2 1H
CPDPRG[2 waltz16
PCPD2 90.00 usec
PLW2 16.43099976 W
PLW12 0.20286000 W
PLW13 0.10204000 W
F2 - Processing parameters
SI 32768
SF 100.6127579 MHz
WDW EM
SSB 0
LB 1.00 Hz
GB 0
PC 1.40
190 180 170 160 150 140 130 120 110 100 90 80 70 60 50 40 30 20 ppm

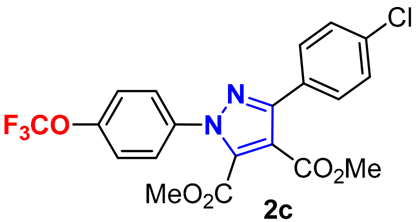

2c

^{1}H NMR (400 MHz, $CDCl_3$)

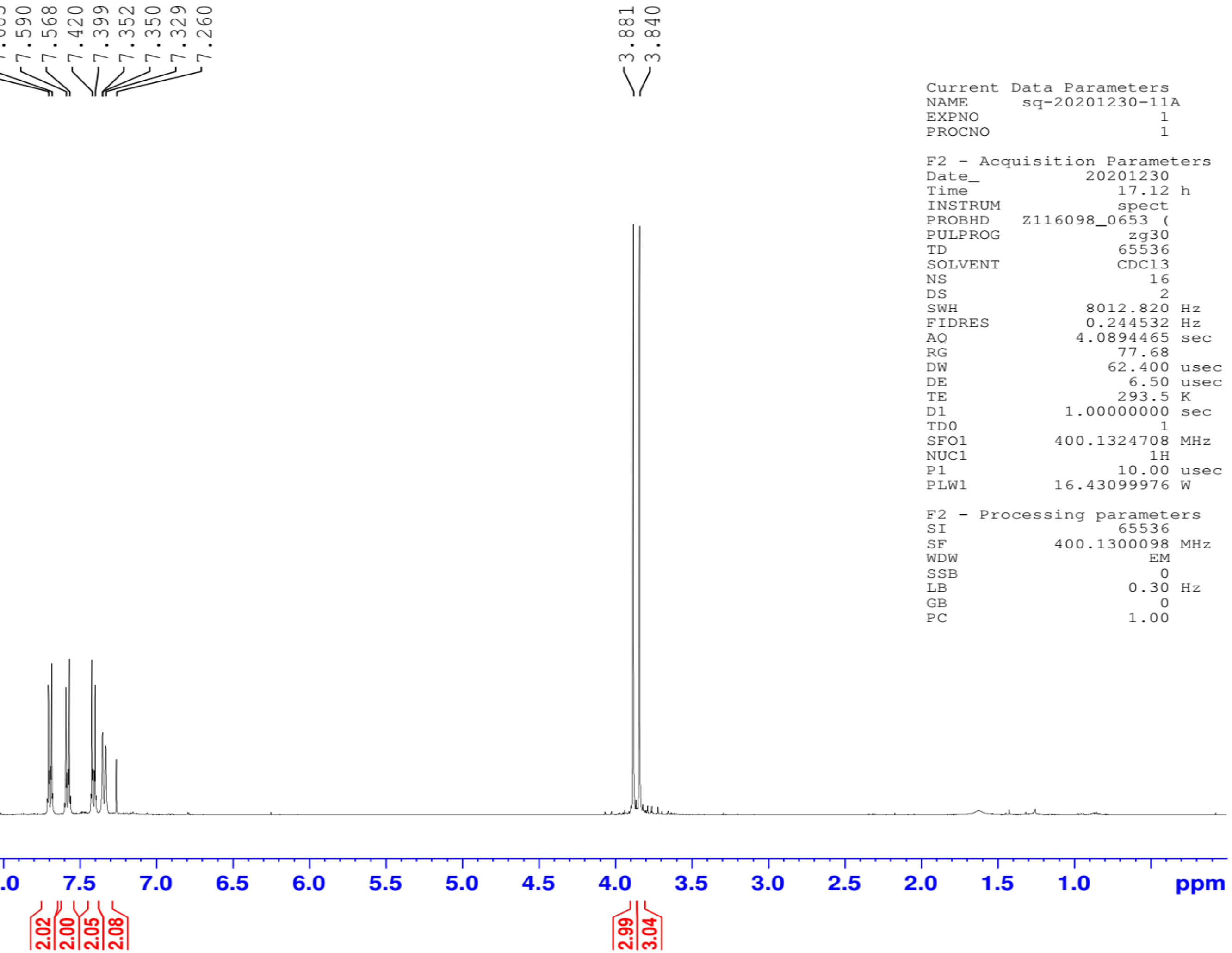

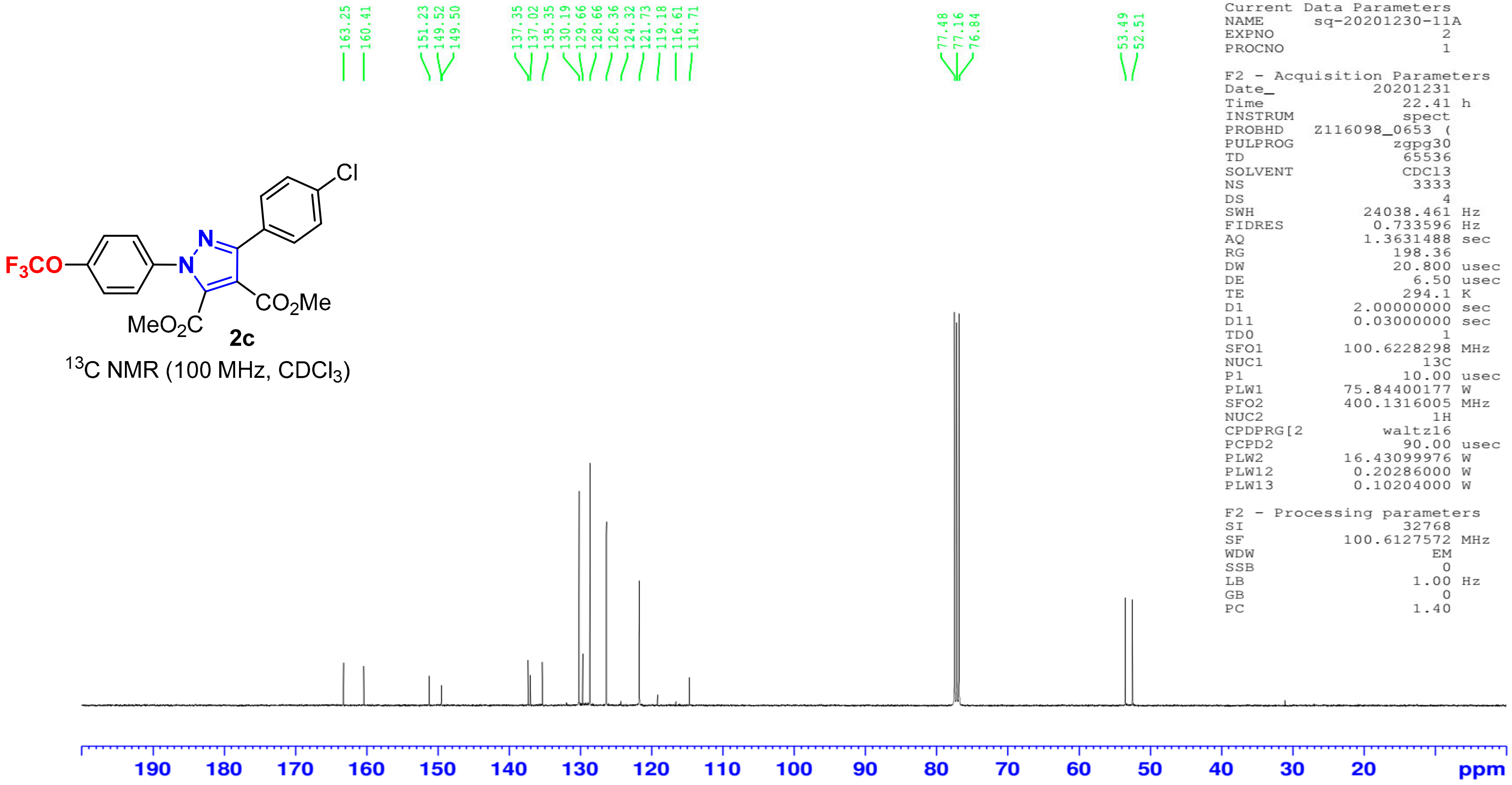
163.25
160.41
151.23
149.52
149.50
137.35
137.02
135.35
130.19
129.66
128.66
126.36
124.32
121.73
119.18
116.61
114.71
77.48
77.16
76.84
53.49
52.51
Cl
N
F3CO
N
CO2Me
MeO2C
2c
13C NMR (100 MHz, CDCl3)
Current Data Parameters
NAME sq-20201230-11A
EXPNO 2
PROCNO 1
F2 - Acquisition Parameters
Date_ 20201231
Time 22.41 h
INSTRUM spect
PROBHD Z116098_0653 (
PULPROG zgpg30
TD 65536
SOLVENT CDCl3
NS 3333
DS 4
SWH 24038.461 Hz
FIDRES 0.733596 Hz
AQ 1.3631488 sec
RG 198.36
DW 20.800 usec
DE 6.50 usec
TE 294.1 K
D1 2.00000000 sec
D11 0.03000000 sec
TD0 1
SFO1 100.6228298 MHz
NUC1 13C
P1 10.00 usec
PLW1 75.84400177 W
SFO2 400.1316005 MHz
NUC2 1H
CPDPRG[2 waltz16
PCPD2 90.00 usec
PLW2 16.43099976 W
PLW12 0.20286000 W
PLW13 0.10204000 W
F2 - Processing parameters
SI 32768
SF 100.6127572 MHz
WDW EM
SSB 0
LB 1.00 Hz
GB 0
PC 1.40
190
180
170
160
150
140
130
120
110
100
90
80
70
60
50
40
30
20
ppm
